# Targeting NAD Metabolism: Rational Design, Synthesis and In Vitro Evaluation of NAMPT/PARP1 Dual-Target Inhibitors as Anti-Breast Cancer Agents

**DOI:** 10.3390/molecules29122836

**Published:** 2024-06-14

**Authors:** Yingpeng Li, Xianxiu Kong, Xinhong Chu, Hui Fu, Xinchi Feng, Chengcheng Zhao, Yanru Deng, Jun Ge

**Affiliations:** 1 College of Chinese Materia Medica, Tianjin University of Traditional Chinese Medicine, Tianjin 301617, China; 2 Tianjin Key Laboratory of Therapeutic Substance of Traditional Chinese Medicine, Tianjin 301617, China; 3 College of Integrative Medicine, Tianjin University of Traditional Chinese Medicine, Tianjin 301617, China; 4 Experimental Teaching and Practical Training Center, Heilongjiang University of Chinese Medicine, Harbin 150040, China

**Keywords:** NAMPT, PARP1, NAD, dual-target inhibitors, breast cancer

## Abstract

The malignancy of breast cancer poses a global challenge, with existing treatments often falling short of desired efficacy. Extensive research has underscored the effectiveness of targeting the metabolism of nicotinamide adenine dinucleotide (NAD), a pivotal molecule crucial for cancer cell survival and growth, as a promising anticancer strategy. Within mammalian cells, sustaining optimal NAD concentrations relies on two key enzymes, namely nicotinamide phosphoribosyltransferase (NAMPT) and poly(ADP-ribose) polymer 1 (PARP1). Recent studies have accentuated the potential benefits of combining NAMPT inhibitors and PARP1 inhibitors to enhance therapeutic outcomes, particularly in breast cancer. In this study, we designed and synthesized eleven novel NAMPT/PARP1 dual-target inhibitors. Among them, compound DDY02 exhibited acceptable inhibitory activities against both NAMPT and PARP1, with IC_50_ values of 0.01 and 0.05 µM, respectively. Moreover, in vitro evaluations revealed that treatment with DDY02 resulted in proliferation inhibition, NAD depletion, DNA damage, apoptosis, and migration inhibition in MDA-MB-468 cells. These results posit DDY02, by targeting NAD metabolism through inhibiting both NAMPT and PARP1, as a promising lead compound for the development of breast cancer therapy.

## 1. Introduction

Nicotinamide adenine dinucleotide (NAD), a crucial coenzyme facilitating hydrogen and electron transfer in redox reactions, plays a pivotal role not only in fundamental cellular processes such as glycolysis, the tricarboxylic acid cycle, and oxidative phosphorylation but also in serine biosynthesis [1]. This involvement ensures that the high energy and building block demands of cancer cells are met. In response to malignant transformation, NAD-dependent enzymes, including poly(ADP-ribose) polymers (PARPs), mono(ADP-ribosyl) transfers (Marts), sirtuins (Sirt1-7), and cyclic ADP-ribose (cADPR) synthases (CD38 and CD157), become increasingly active [2]. This activation aids cancer cells in DNA repair, oncogene expression, and immune evasion by consuming increased amounts of NAD [3]. Consequently, cancer cells exhibit a heightened demand for NAD, rendering them particularly susceptible to interventions targeting NAD metabolism, an approach that has proven effective in cancer treatment.

NAD can be synthesized de novo from L-tryptophan through a series of enzymatic reactions. However, this pathway often cannot meet the urgent cellular demand for NAD promptly. Nicotinic acid (NA) and nicotinamide (NAM), both forms of vitamin B3, are vital precursors in NAD synthesis. While the Preiss–Handler pathway, utilizing NA as a precursor, is conserved in mammals, mammals prefer the salvage pathway, utilizing NAM as a precursor, to synthesize NAD due to its economy and time efficiency [4].

**Figure 1:**
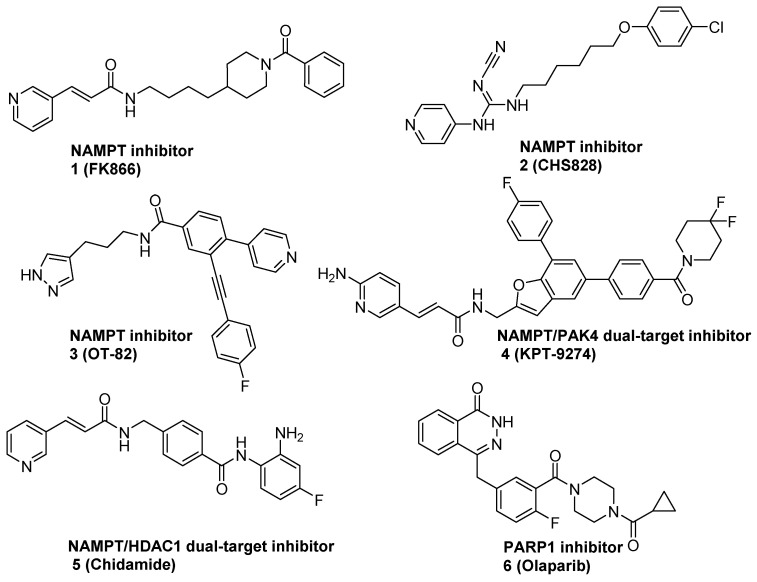
The structures of NAMPT inhibitors and PARP1 inhibitors.

Nicotinamide phosphoribosyltransferase (NAMPT), the rate-limiting enzyme in the salvage pathway, catalyzes the conversion of NAM and PRPP to produce nicotinamide mononucleotide (NMN) [5]. Since the discovery of FK866 (see compound **1** of Figure 1) as a potent nanomolar NAMPT inhibitor in 2002, both academia and industry have developed various NAMPT inhibitors [6,7,8]. However, initial inhibitors such as FK866 and CHS828 (see compound **2** of Figure 1) were discontinued in phase II clinical trials due to inadequate efficacy, severe dose-limiting toxicity, and adverse pharmacokinetic properties. More recently, OT-82 (see compound **3** of Figure 1) was introduced in 2019 with a novel skeletal structure and is currently in phase I clinical trials for lymphoma treatment [9].

Given the high heterogeneity of tumors, targeting a single pathway often yields limited efficacy. Dual-target drugs, simultaneously addressing two targets, can produce synergistic effects and have emerged as promising cancer treatment strategies [10]. Recent research has highlighted the potential of dual-target NAMPT inhibitors in improving cancer treatment outcomes [6]. For example, KPT-9274 (see compound **4** of Figure 1), a dual-target inhibitor of NAMPT and p21-associated kinase 4 (PAK4), has entered clinical trials for cancer treatment. Notably, Chidamide (see compound **5** of Figure 1), initially approved by CFDA in 2014 as an HDAC inhibitor for lymphoma treatment, was later found to be an NAMPT/HDAC dual-target inhibitor [11]. Dual-target NAMPT inhibitors may hold better application prospects in cancer treatment compared to single-target NAMPT inhibitors.

PARP1 utilizes NAD as a co-substrate to catalyze the poly(ADP-ribosyl)ation of proteins, a key mechanism in repairing DNA single-strand breaks [12]. Due to their high proliferation rates, cancer cells face greater DNA damage pressure than normal cells, making them heavily reliant on DNA damage repair mechanisms. PARP1 inhibitors capitalize on the concept of synthetic lethality, specifically targeting cancer cells deficient in homologous recombination repair [13,14]. Since the discovery of Olaparib (see compound **6** of Figure 1), the first approved PARP1 inhibitor in 2014 for ovarian cancer treatment, six PARP1 inhibitors have been launched. However, clinical applications have revealed issues such as low efficacy, significant adverse reactions, and drug resistance. Medicinal chemists have developed various dual-target PARP1 inhibitors as an effective strategy to address these shortcomings. These dual target inhibitors are thought to achieve a synergistic effect with PAPR1 inhibition by inducing homologous recombination defects or additional DNA damage [15].

Notably, the interconnectedness of NAMPT, PARP1, and NAD metabolism provides a rationale for developing dual-target inhibitors targeting both pathways. The activity of PARP1 results in the consumption of NAD, thereby linking DNA repair mechanisms directly to NAD metabolism. Consequently, NAMPT and PARP1 are interconnected through their roles in sustaining NAD levels and the cellular response to DNA damage. Disruption in the function of either enzyme can lead to altered NAD availability, affecting cell viability, energy metabolism, and the capacity to respond to DNA damage stress. This interplay underscores their significance as potential therapeutic targets in the treatment of cancer. Clinically applied PARP1 inhibitors typically function as competitive inhibitors of the substrate NAD. The reduction in NAD levels by NAMPT inhibitors enhances the inhibitory capacity of PARP1 inhibitors, introducing a novel synergistic mechanism [16]. The combined use of NAMPT and PARP1 inhibitors has demonstrated synergistic effects in treating various cancers, including breast cancer, Ewing sarcoma, and ovarian cancer [17,18,19]. This promising approach holds potential for addressing the limitations associated with traditional PARP1 inhibitors and improving the efficacy of cancer treatment.

**Figure 2:**
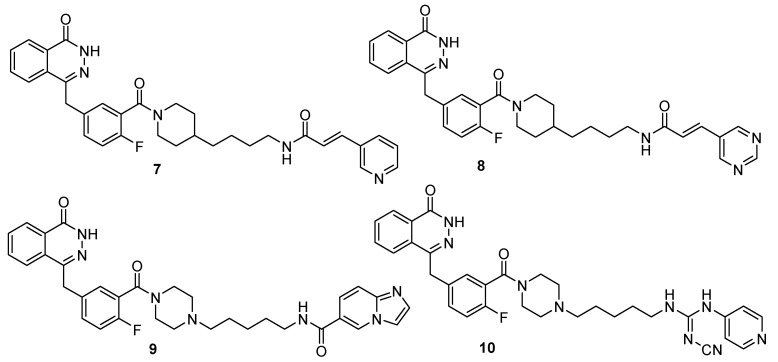
The structures of NAMPT/PARP1 dual-target inhibitors from patents.

In 2019, a patent disclosed a class of compounds containing phthalazinone as PARP1 inhibitors (see compounds **7**~**8** of Figure 2), exhibiting excellent proliferation inhibition against Olaparib-resistant MDA-MB-468 cells [20]. Although this patent did not specify whether these compounds inhibit NAMPT, pharmacophore analysis suggests they may act as NAMPT/PARP1 dual-target inhibitors. We hypothesize that their notable effectiveness in overcoming drug resistance results from simultaneously inhibiting NAMPT and PARP1. Subsequently, in 2021, another patent revealed compounds with the same core structure as potent NAMPT/PARP1 dual-target inhibitors (see compounds **9**~**10** of Figure 2), demonstrating strong proliferation inhibition across three breast cancer cell lines [21].

To date, no scholarly articles have explored the design of NAMPT/PARP1 dual-target inhibitors, and the patent literature alone provides limited insight into the benefits of this novel class of inhibitors in cancer treatment. In response, our study undertook the design and synthesis of eleven NAMPT/PARP1 dual-target inhibitors with novel core structures. We have meticulously summarized the structure–activity relationships and extensively investigated their efficacy and underlying mechanisms in the context of breast cancer treatment. It is our expectation that this work will serve as a valuable reference, enriching the understanding and application of targeting NAD metabolism in cancer treatment.

## 2. Results and Discussion

### 2.1. Design

**Figure 3:**
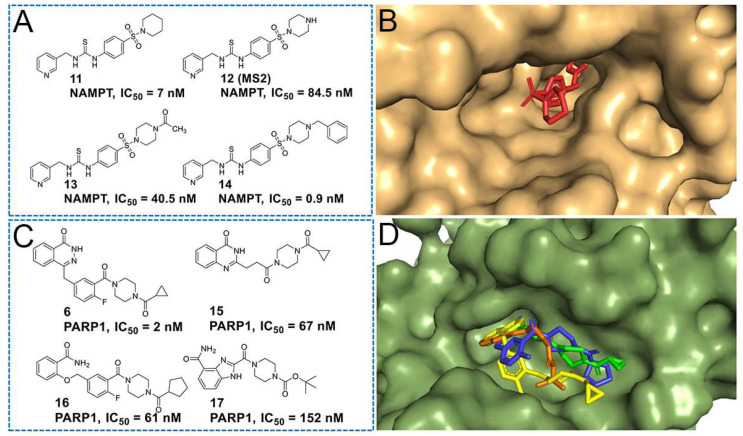
The modeled poses of NAMPT inhibitors and PARP1 inhibitors in the binding site. (**A**) The structures of NAMPT inhibitors; (**B**) the modeled pose of NAMPT inhibitor **11** (red) in the binding site of NAMPT; (**C**) the structures of PARP1 inhibitors; (**D**) the modeled poses of PARP1 inhibitors **6** (yellow), **15** (green), **16** (blue), and **17** (orange) in the binding site of PARP1.

As shown in Figure 3A, Zheng’s group [22] identified compound **11**, a thiourea derivative, as a potent NAMPT inhibitor. Detailed analysis of its eutectic structure with NAMPT was conducted using X-ray diffraction (PDB ID: 4JR5). Subsequently, an independent research group led by Sheng synthesized forty-six derivatives of **11**, including compounds **12** to **14**, and undertook a comprehensive study of their structure–activity relationship [23]. Results from these studies indicated that modifications made to the terminal nitrogen of the piperazine moiety of **12** were well tolerated, maintaining effective NAMPT inhibition. Figure 3B illustrated the docking analysis of the binding mode between **12** and NAMPT. Notably, the piperazine structure was observed to extend into the solvent-exposed region, indicating that the introduction of additional structures onto the piperazine would not compromise the inhibition ability of NAMPT.

Building upon these insights, Sheng’s group utilized **12** and its derivatives as templates for the design of multifunctional molecules targeting NAMPT. This innovative approach included the development of dual-target inhibitors, proteolysis-targeting chimeras (PROTACs), and fluorescent probes, showcasing the versatility and potential applications of these compounds in the context of NAMPT-related research [24,25,26,27,28,29].

As depicted in Figure 3C, compounds **6**, **15**~**17** comprised a class of PARP1 inhibitors featuring a piperazine structure [30,31,32,33,34]. As shown in Figure 3D, molecular docking analysis, examining the binding modes of these compounds with PARP1, revealed that the piperazines in these inhibitors formed an amide bond connecting to the PARP1 pharmacophore, anchoring the molecule at the active binding site. Meanwhile, the tail group of the piperazine extended outward toward the solvent.

**Figure 4:**
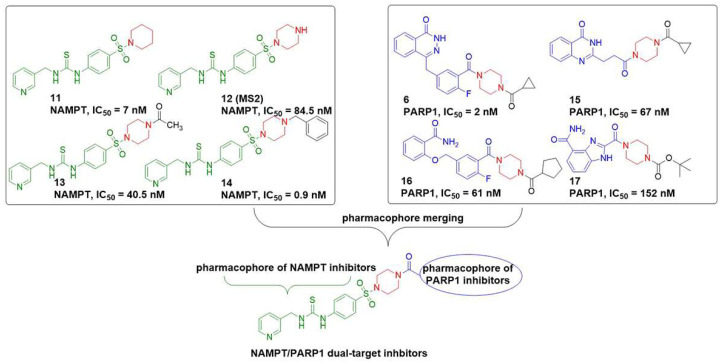
The design strategy of NAMPT/PARP1 dual-target inhibitors. The pharmacophores of NAMPT inhibitors and PARP1 inhibitors are shown in green and blue, respectively.

Considering both binding modes and chemical tractability, the piperazine emerged as a suitable bridge for merging pharmacophores, allowing for the integration of **12** and compounds **6** and **15**~**17**. Building on these insights, as illustrated in Figure 4, we employed the pharmacophores of PARP1 inhibitors **6** and **15**~**17** (indicated by the blue sections) and incorporated them into **12**, leading to the design of a series of NAMPT/PARP1 dual-target inhibitors, denoted as **DDY01**~**DDY11**.

### 2.2. Biological Screening

#### 2.2.1. Enzymatic Activities against NAMPT and PARP1

In Table 1, the inhibitory effects of eleven target compounds on NAMPT and PARP1 were examined. We first examined the enzyme activity inhibition of these compounds against PARP1 and NAMPT at a single concentration. Considering that compounds with low inhibition rates are not suitable for subsequent validation of the synergistic effect of dual-target inhibitors, the IC_50_ values for enzyme inhibition were determined for selected compounds exhibiting significant inhibition rates. Compound **DDY01**, derived from the amalgamation of the pharmacophores of compound **6** (Olaparib) and compound **12**, demonstrated a noteworthy inhibition rate of 83% at 0.1 μM against NAMPT. However, **DDY01** exhibited a 4.5-fold decrease in inhibitory capacity against PARP1 (IC_50_ = 0.009 μM) compared to Olaparib (IC_50_ = 0.002 μM). Urea derivative **DDY02**, designed based on bioisosterism principles, displayed excellent NAMPT inhibitory activity (IC_50_ = 0.01 μM) but experienced a 5.5-fold decrease in inhibitory capacity against PARP1 compared to **DDY01**. The fusion of the pharmacophores of compound **12** with compound **15** resulted in compound **DDY03**, which demonstrated enhanced inhibitory capacity against both NAMPT (IC_50_ = 0.055 μM) and PARP1 (IC_50_ = 0.025 μM), outperforming the parent compounds. Compounds **DDY04**~**DDY09**, which integrate the pharmacophores of compound **16** with **12**, were also evaluated. Thiourea **DDY04** and urea **DDY05** exhibited excellent NAMPT inhibitory capacity at low concentrations (84%~86% at 0.1 μM) as well as moderate PARP1 inhibitory capacity. Interestingly, introducing a methylene group between the benzene ring and the sulfonyl group in **DDY05** led to **DDY06**, which showed an increased inhibition rate of PARP1 (89% at 0.5 μM) but a significantly decreased inhibition rate of NAMPT (48% at 0.1 μM). Pursuing enhancements in PARP1 inhibitory capacity, derivatives **DDY07** to **DDY09** were designed with varying numbers of fluorine substitutions on the benzene ring, a modification suggested by the literature to enhance PARP1 inhibition [30]. The results indicated that these modifications improved PARP1 inhibition but did not affect NAMPT activity. Finally, **DDY10** and **DDY11**, which incorporate a benzimidazole core derived from compound **17**, showed distinct inhibitory profiles. Thiourea **DDY10** exhibited poor inhibition of NAMPT (7% at 0.1 μM) and modest inhibition of PARP1 (55% at 0.5 μM). In contrast, urea **DDY11** displayed significantly enhanced inhibitory capacity against both NAMPT (IC_50_ = 0.034 μM) and PARP1 (IC_50_ = 0.014 μM), achieving a balanced inhibitory profile against both targets.

#### 2.2.2. Molecular Docking Study

Considering that **DDY02** and **DDY11** exhibited balanced inhibitory capacities against NAMPT and PARP1, making them suitable candidates for further binding mode analysis, we conducted a molecular docking study.

**Figure 5:**
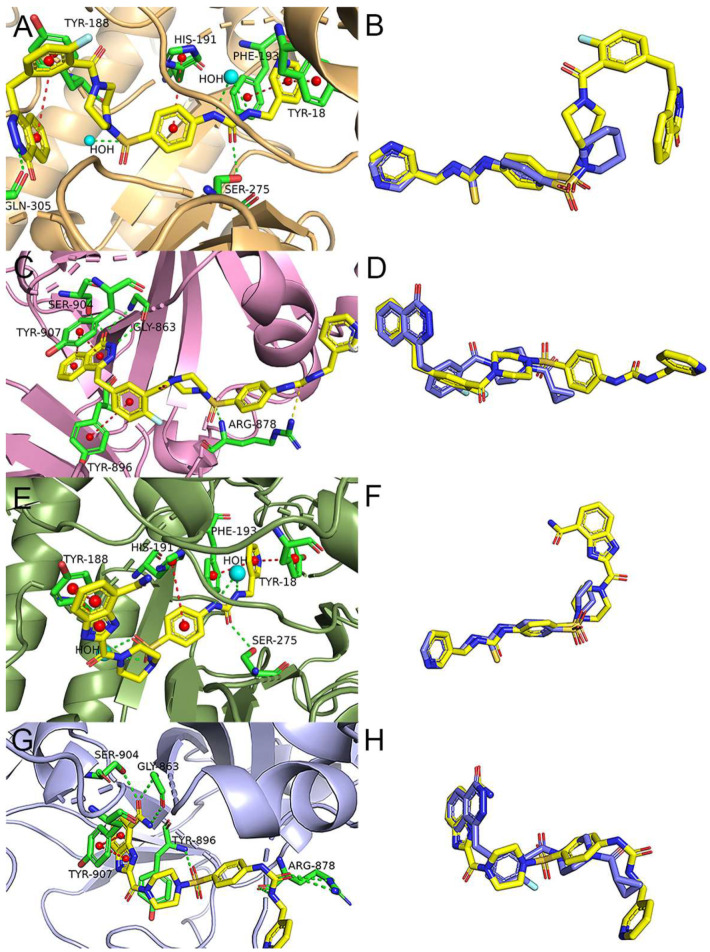
Binding mode analysis of **DDY02** and **DDY11**. (**A**) Binding mode of **DDY02** with NAMPT (PDB ID: 4JR5). (**B**) Conformation of **DDY02** at the binding site of NAMPT. (**C**) Binding mode of **DDY02** with PARP1 (PDB ID: 5DS3). (**D**) Conformation of **DDY02** at the binding site of PARP1. (**E**) Binding mode of **DDY11** with NAMPT (PDB ID: 4JR5). (**F**) Conformation of **DDY11** at the binding site of NAMPT. (**G**) Binding mode of **DDY11** with PARP1 (PDB ID: 5DS3). (**H**) Conformation of **DDY11** at the binding site of PARP1. Hydrogen bonds are shown by green dashed lines and pi–pi stacking interactions are shown by red dashed lines. **DDY02** and **DDY11** are shown in yellow sticks, the ligands in the co-crystals are shown in purple sticks. The figure was generated using PyMol (version 2.5).

As depicted in Figure 5A,B, the docking results revealed a high degree of conformational similarity between **DDY02** and **11** at the active site. Both compounds exhibited analogous binding modes with NAMPT. Specifically, the pyridine moiety formed a sandwich-shaped pi–pi stacking interaction with Phe193 and TYR18′, while the oxygen in the urea group engaged in a hydrogen bond with Ser275. Additionally, the nitrogen of the urea group and the oxygen of the sulfonyl group established hydrogen bond interactions with water molecules. Notably, the terminal 4-benzyl phthalazinone structure adopted a “J” shape conformation, facilitating interactions with Gly305 and Tyr188 at an optimal distance.

As illustrated in Figure 5C,D, the binding mode of **DDY02** and PARP1 resembled that of Olaparib. The amide of phthalazinone formed hydrogen bond interactions with Gly863 and Ser904, and the benzene rings engaged in pi–pi stacking interactions with Tyr907 and Tyr896. Further hydrogen bond interactions were observed between the oxygens of the sulfonyl and urea groups and Arg878. The terminal pyridine structure extended into the solvent-exposed region. It is noteworthy that **DDY02** did not completely overlap with Olaparib. Instead, the fluorine-substituted benzene ring of **DDY02** underwent a T-shaped pi–pi interaction with Tyr896 after a 90-degree rotation at the dihedral angle.

We also simulated the binding mode of **DDY11** at the active sites of the target proteins using molecular docking. As shown in Figure 5E–H, the results indicated that **DDY11**, similar to **DDY02**, formed hydrogen bonds, van der Waals forces, pi–pi stacking, and hydrophobic interactions with key residues of NAMPT and PARP1. All these docking results indicated that **DDY02** and **DDY11** effectively bound with both NAMPT and PARP1, supporting the feasibility of our dual-target drug design strategy.

#### 2.2.3. Cytotoxicity Assay

We assessed the proliferation inhibition abilities of the synthesized target compounds, FK866, Olaparib, and their combination on three types of breast cancer cells. As presented in Table 2, FK866 exhibited a more potent inhibitory effect on the proliferation of all three breast cancer cell lines compared to Olaparib. Interestingly, the combination of FK866 and Olaparib demonstrated enhanced proliferation inhibition, showcasing a synergistic effect. Among the cell lines tested, the triple-negative breast cancer (TNBC) cell lines, MDA-MB-231 and MDA-MB-468, displayed higher sensitivity to FK866, Olaparib, and their combination, relative to MCF-7 cells. Among these target compounds, compound **DDY10** displayed the most substantial proliferation inhibition in MDA-MB-231 cells, surpassing the effects of Olaparib but slightly less potent than FK866. Despite its limited NAMPT inhibitory activity, **DDY10**’s significant anti-proliferative effects in MDA-MB-231 cells suggest that it may operate through mechanisms other than direct NAMPT inhibition. **DDY02** exhibited the strongest inhibitory effect on MDA-MB-468 cell proliferation among all target compounds. While **DDY02**, **DDY06**, and **DDY11** showed comparable inhibition in MCF-7 cells, our synthesized compounds generally demonstrated weaker anti-proliferative effects in MCF-7 cells compared to both MDA-MB-468 and MDA-MB-231 cells. This aligns with Ashworth’s team’s findings, which highlighted a significant difference in the synergistic effect of NAMPT inhibitors combined with PARP1 inhibitors between MDA-MB-468 and MDA-MB-231 cells [17]. Their research revealed that MDA-MB-468 cells exhibited greater sensitivity to the combination of FK866 and Olaparib compared to MDA-MB-231 cells. Based on these observations, we selected MDA-MB-468 cells and **DDY02**, the most potent compound, for subsequent mechanistic exploration experiments.

The cytotoxic effects of synthetic compounds on normal human breast epithelial cells (MCF-10A) were evaluated in this study. As shown in Table 3, our findings demonstrated that FK866, Olaparib, and **DDY02** exhibited no selectivity in their cytotoxic activity between breast cancer cells and normal cells. However, flow cytometry analysis revealed that **DDY02** induced a dose-dependent G1-phase cell cycle arrest in MDA-MB-468 cells (Figure S5). This cell cycle arrest capacity is likely advantageous for selective targeting of cancer cells, given their higher proliferation rate compared to normal cells. These results suggest that the selectivity of such dual-target inhibitors warrants further investigation.

#### 2.2.4. **DDY02** Suppressed Colony Formation

**Figure 6:**
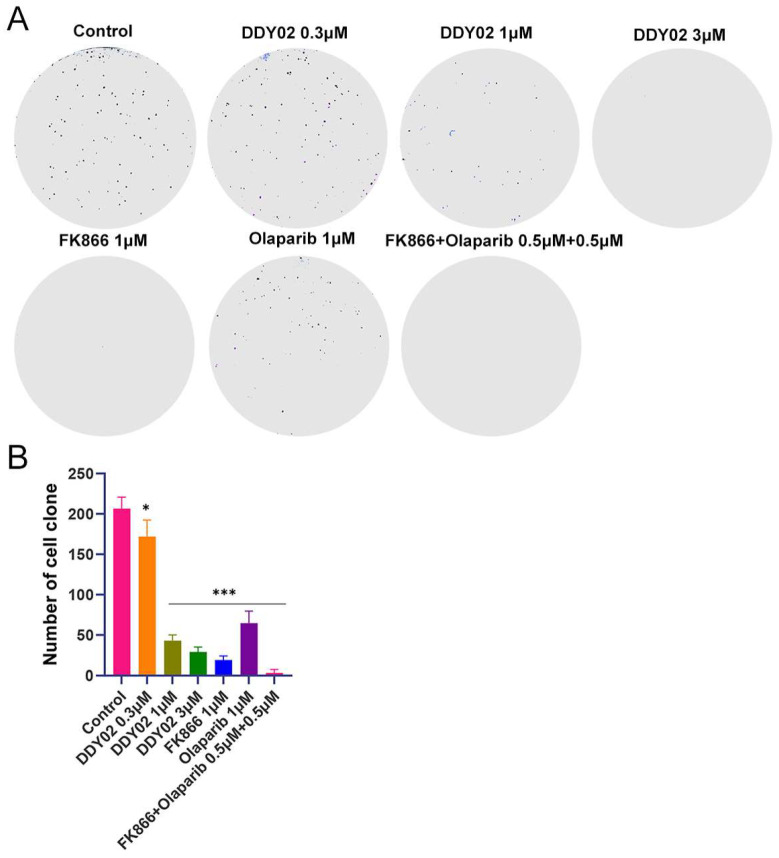
**DDY02** inhibited colony formation in MDA-MB-468 cells. (**A**) Colony formation assay of MDA-MB-468 cells treated with FK866, Olaparib, FK866 + Olaparib, and **DDY02**. (**B**) The number of cell clones in each population. Data are expressed as the mean ± SD (*n* = 3). * *p* < 0.05 and *** *p* < 0.001 vs. control group.

We assessed the impact of **DDY02**, FK866, Olaparib, and the combination of FK866 and Olaparib on the proliferation of MDA-MB-468 cells using a colony formation assay. As depicted in Figure 6A,B, the results obtained from the colony formation assay were consistent with those from the MTT assay. **DDY02** demonstrated a dose-dependent suppression of colony formation in MDA-MB-468 cells. Notably, at a concentration of 1 μM, **DDY02** exhibited a more potent ability to inhibit colony formation compared to Olaparib, albeit slightly less effective than FK866. It is noteworthy that the combination of FK866 and Olaparib displayed a pronounced inhibition of colony formation, indicating a synergistic effect on the proliferative capacity of MDA-MB-468 cells.

#### 2.2.5. NMN Reversed the Efficacy of Compound **DDY02**

**Figure 7:**
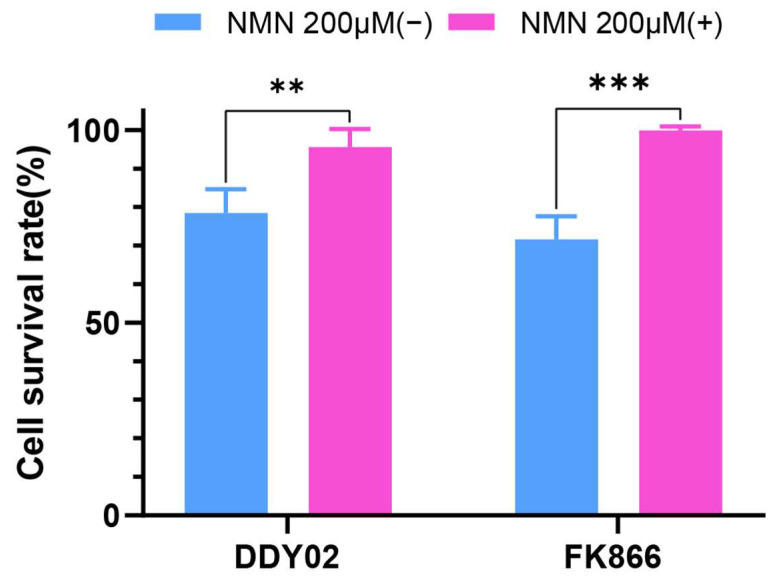
NMN reversed the efficacy of compound **DDY02**. Data are expressed as the mean ± SD (*n* = 3). ** *p* < 0.01 and *** *p* < 0.001 vs. control group.

NMN is the direct product of the NAMPT-catalyzed reaction. Excessive NMN can counteract the inhibitory effect of NAMPT inhibition. As depicted in Figure 7, preincubation with 200 μM of NMN reversed the cytotoxic effects of **DDY02** and FK866 on MDA-MB-468 cells. The reversal of **DDY02**’s efficacy by NMN underscores the compound’s mechanism of action and confirms the critical role of NAMPT inhibition in mediating its therapeutic effects. This observation also emphasizes the potential of NMN as a modulator in therapeutic scenarios involving NAMPT-targeted treatments.

#### 2.2.6. **DDY02** Reduced Cellular NAD Levels

**Figure 8:**
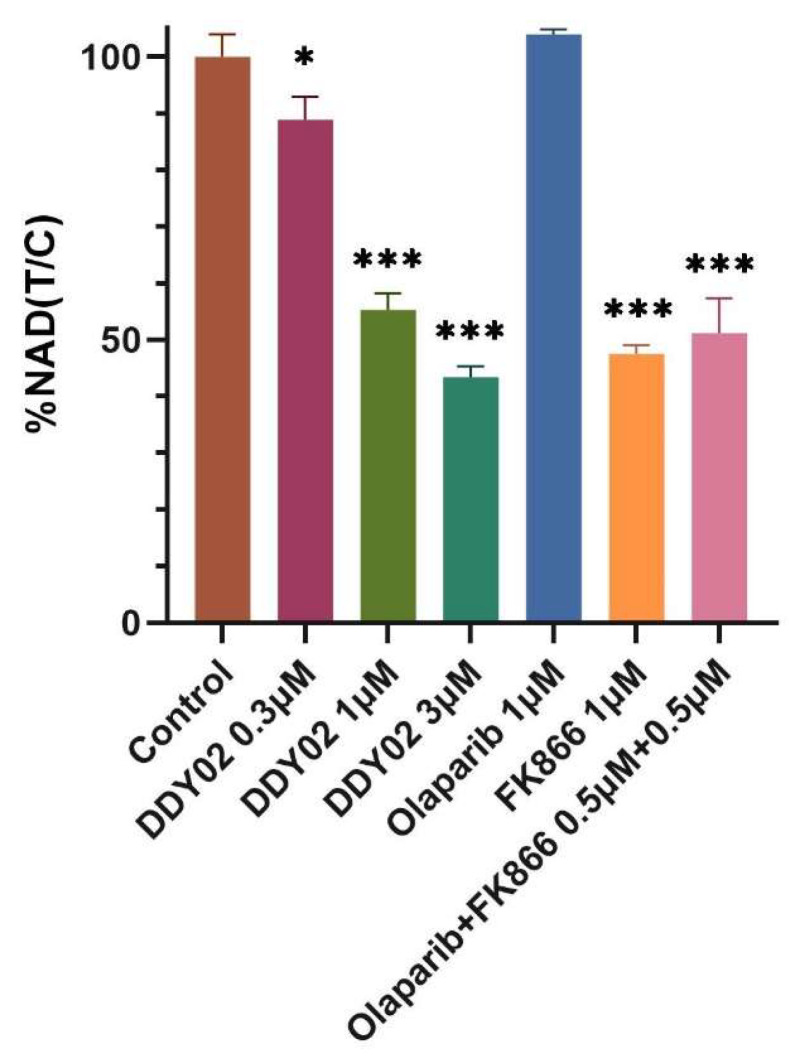
Compound **DDY02** reduced the NAD level in MDA-MB-468 cells. Data are expressed as the mean ± SD (*n* = 3). * *p* < 0.05 and *** *p* < 0.001 vs. control group.

We investigated the impact of **DDY02**, FK866, Olaparib, and the combination of FK866 and Olaparib on NAD levels in MDA-MB-468 cells. As illustrated in Figure 8, **DDY02** reduced cellular NAD levels in a dose-dependent manner 24 h post-treatment. At a concentration of 1 μM, **DDY02**’s capacity to deplete NAD was marginally less potent than FK866. In contrast, Olaparib administered alone at the same concentration did not alter intracellular NAD levels compared to the control. Interestingly, the cellular NAD levels in the combination group (0.5 μM FK866 + 0.5 μM Olaparib) were comparable to those in the FK866 group (1 μM FK866), demonstrating that the combination enhanced the NAD-depleting ability.

#### 2.2.7. **DDY02** Induced DNA Damage

DNA single-strand damage typically activates PARP1, which binds to the damage site and catalyzes the poly(ADP-ribosyl)ation of histones, facilitating chromatin remodeling and the recruitment of DNA repair enzymes. However, inhibiting PARP1 activity prevents the repair of these single-strand breaks, potentially converting them into double-strand breaks during DNA replication at the replication fork. Histone H2AX phosphorylation at Ser139, resulting in γ-H2AX, is an early and sensitive marker of DNA double-strand breaks.

**Figure 9:**
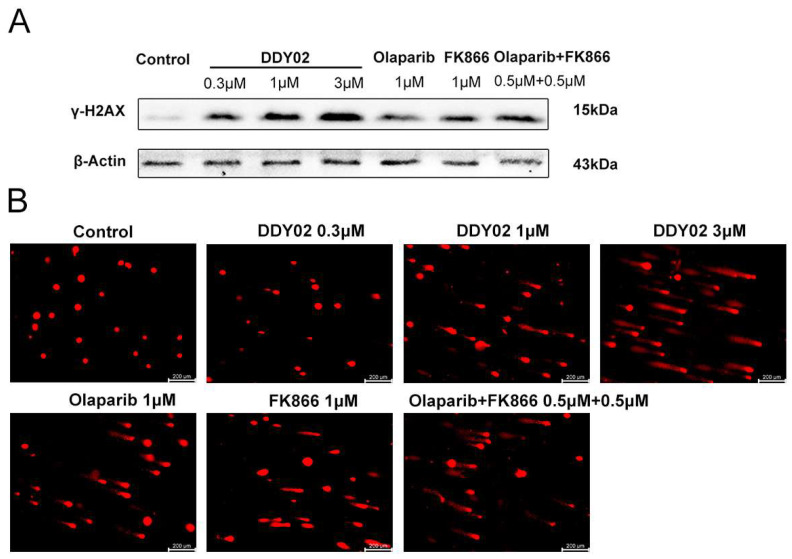
**DDY02** induced DNA damage. (**A**) The effects of FK866, Olaparib, FK866 + Olaparib, and **DDY02** on the expression of γ-H2AX analyzed by Western blot. (**B**) Effects of FK866, Olaparib, FK866+ Olaparib, and **DDY02** on the DNA damage of MDA-MB-468 cells. The scale bar in the figure represents 200 μm.

As depicted in Figure 9A, a Western blot assay demonstrated that **DDY02** induced a dose-dependent accumulation of γ-H2AX in MDA-MB-468 cells. At a concentration of 1 μM, **DDY02**, Olaparib, FK866, and their combination group each promoted the accumulation of γ-H2AX, with **DDY02** showing the most substantial effect and Olaparib the least. Remarkably, the protein levels of γ-H2AX from cells treated with the combination group were higher than those treated with Olaparib or FK866 alone, indicating a synergistic effect when simultaneously inhibiting NAMPT and PARP1, leading to increased DNA damage.

Further confirming these results, the comet assay (Figure 9B) directly visualized the DNA damage in MDA-MB-468 cells (Figure 9B). Consistent with the Western blot data, **DDY02** treatment led to increased DNA damage in a concentration-dependent manner. At a concentration of 1 μM, the extent of DNA damage in the combination group was more pronounced than that observed with Olaparib or FK866 alone, highlighting a synergistic effect.

#### 2.2.8. **DDY02** Induced Apoptosis

To investigate whether **DDY02** induced apoptosis in MDA-MB-468 cells, we performed the annexin V/PI double staining assay, and the staining results were analyzed by flow cytometry, dividing the results into four quadrants (Q1~Q4). In this representation, Q1 represents necrosis, Q2 represents late apoptosis, Q3 represents early apoptosis, and Q4 represents living cells.

**Figure 10:**
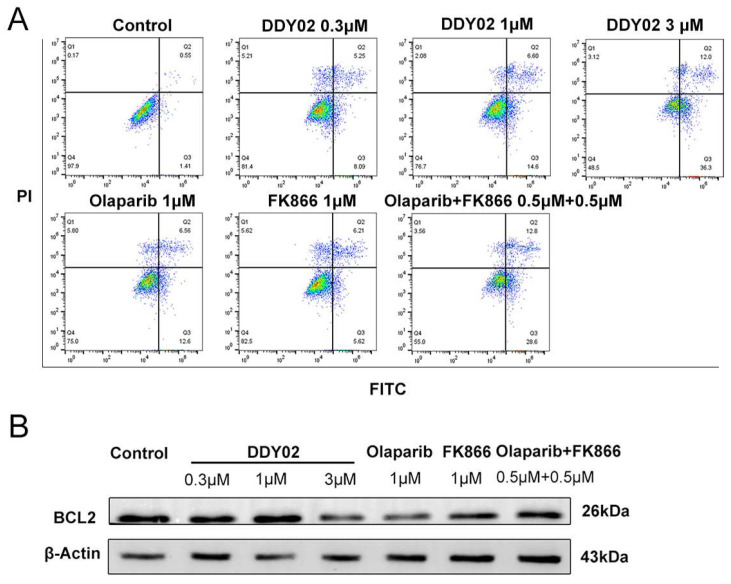
**DDY02** induced apoptosis in MDA-MB-468 cells. (**A**) Cell apoptosis induced by FK866, Olaparib, FK866 + Olaparib, **DDY02**. (**B**) The effects of FK866, Olaparib, FK866 + Olaparib, and **DDY02** on the expression of BCL2 analyzed by Western blot.

As illustrated in Figure 10A, following treatment with **DDY02** at concentrations of 0.3, 1, and 3 µM, the percentage of apoptotic cells (Q2 + Q3) was 13.34%, 21.20%, and 48.3%, respectively, displaying a dose-dependent trend. At a concentration of 1 μM, **DDY02** and the combination of Olaparib and FK866 exhibited higher levels of apoptosis compared to Olaparib and FK866 alone. These results indicate that **DDY02** efficiently induces apoptosis in MDA-MB-468 cells and shows a synergistic effect at the cellular level.

Further exploration was focused on the B-cell lymphoma 2 (BCL2) protein, an anti-apoptotic protein highly expressed in TNBC that contributes to drug resistance by inhibiting drug-induced mitochondrial apoptosis [35,36]. Given that NAMPT inhibition leading to NAD deficiency and PARP1 inhibition leading to DNA damage can both induce mitochondrial apoptosis in cancer cells, we investigated whether **DDY02** affects BCL2 expression using Western blot analysis [37,38]. As shown in Figure 10B, **DDY02** inhibited BCL2 expression in a dose-dependent manner. At a concentration of 1 μM, Olaparib significantly inhibited the expression of the BCL2 protein, while FK866 and the combination group had no effect on the expression of BCL2. These findings suggest that **DDY02**-induced apoptosis may be mediated by inhibiting PARP1-induced DNA damage, thereby activating the BCL2-mediated mitochondrial apoptosis pathway.

#### 2.2.9. **DDY02** Inhibited Cell Migration

Breast cancer-related deaths are primarily attributed to tumor metastasis, emphasizing the critical importance of preventing metastasis to enhance patient survival and prognosis. The upregulation of NAMPT plays a significant role in various cellular processes that collectively contribute to the metastatic behavior of breast cancer cells. Firstly, increased NAMPT levels elevate NAD levels, supporting enhanced energy production in cells. This heightened energy state may provide breast cancer cells with the resources necessary for increased migratory and invasive activities associated with metastasis. Furthermore, SIRT1, an NAD-dependent deacetylase, has been linked to promoting metastasis in breast cancer, and elevated NAD levels can activate SIRT1, influencing the expression of genes associated with metastasis [39]. Additionally, upregulated NAMPT is associated with increased production of inflammatory cytokines, creating a microenvironment conducive to tumor metastasis. The epithelial–mesenchymal transition (EMT) is a biological process where cancer cells undergo changes that make them more migratory and invasive. PARP1 is linked to the regulation of EMT in breast cancer cells, promoting the acquisition of mesenchymal traits conducive to metastasis. The PARP1 inhibitor, Olaparib, has been shown to prevent EMT induced by TGF-β in cancer cells [40].

**Figure 11:**
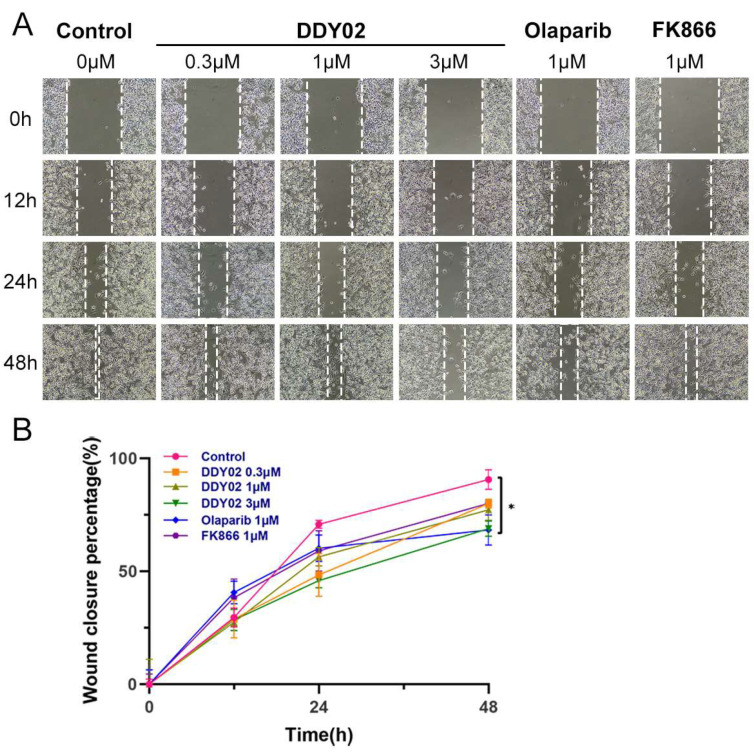
**DDY02** inhibits the migration of MDA-MB-468 cells in a concentration-dependent manner. (**A**) Effects of **DDY02**, FK866, and Olaparib on the migration of MDA-MB-468 cells. (**B**) Wound closure percentage of **DDY02**, FK866, and Olaparib in MDA-MB-468 cells. Data are expressed as the mean ± SD (*n* = 3). * *p* < 0.05 vs. control group.

We evaluated the impact of **DDY02**, FK866, and Olaparib on the migratory potential of MDA-MB-468 cells using a wound scratch assay. As depicted in Figure 11A,B, the control group showed complete filling of the wound area by MDA-MB-468 cells 48 h post-scratching. Notably, at a concentration of 1 μM, Olaparib exhibited the most robust inhibitory effect on the migration of MDA-MB-468 cells. In comparison, **DDY02** showed a slightly less pronounced inhibitory effect on cell migration, while FK866 demonstrated the weakest inhibitory activity. Interestingly, the migration-inhibitory effect of **DDY02** on MDA-MB-468 cells was further augmented at a concentration of 3 μM, demonstrating the concentration-dependent nature of **DDY02**’s inhibitory action. This highlights the potential of **DDY02** as an effective agent in controlling the migratory behavior of breast cancer cells, which is crucial for preventing metastasis and improving clinical outcomes.

## 3. Materials and Methods

### 3.1. Chemistry

**Scheme 1:**
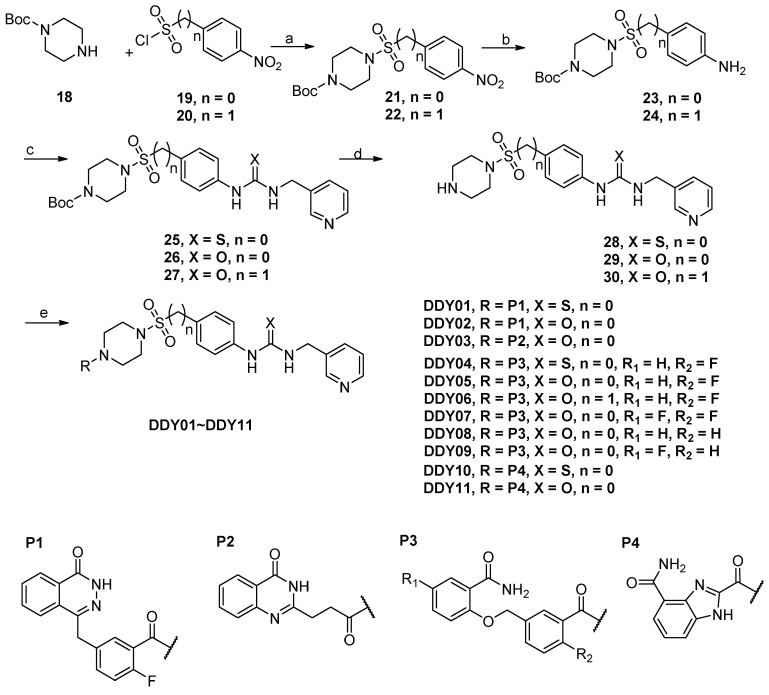
Synthesis of designed NAMPT/PAPR1 dual-target inhibitors. Reagents and conditions: (a) pyridine/DCM (1:1), rt, 2 h; (b) Pd/C, H_2_, EtOAc, rt, 12 h; (c) thiophosgene or triphosgene, NaHCO_3_, CH_2_Cl_2_, C_2_H_5_OH, rt, 1 h; (d) TFA, CH_2_Cl_2_, rt,1 h; (e) EDCI, HOBT, TEA, DMF, rt, overnight.

The synthesis of target compounds is outlined in Scheme 1. Commencing with commercially available 1-Boc-piperazine **18** and sulfonyl chlorides **19**~**20**, compounds **21**~**22** were obtained through sulfonylation reactions. The reduction of the nitro groups in compounds **21**~**22** yielded compounds **23**~**24**. Thiourea **25** was synthesized by reacting **23** with thiophosgene, followed by the addition of pyridin-3-ylmethanamine. Ureas **26**~**27** were produced by reacting **23**~**24** with triphosgene and pyridin-3-ylmethanamine using a procedure analogous to that employed for intermediate **25**. Subsequently, the removal of Boc protections was achieved with trifluoroacetic acid, resulting in the key intermediates **28**~**30**. Additionally, the corresponding intermediate acids (P1~P4) were synthesized following reported procedures [30,31,32,33]. Finally, the condensation of these acids and intermediates (**28**~**30**) led to the formation of the target compounds **DDY01**~**DDY11**.

#### 3.1.1. *tert*-Butyl 4-((4-Nitrophenyl)sulfonyl)piperazine-1-carboxylate (Compound **21**)

To a solution of **18** (170 mg, 0.9 mmol) in tetrahydrofuran (5 mL) and pyridine (3 mL), 4-nitrobenzenesulfonyl chloride (200 mg, 0.9 mmol) was added at 25 °C. After stirring at 60 °C for 30 min, the solution was poured into dichloromethane (20 mL). The organic layer was washed successively with 2 N HCl (10 mL) and brine (20 mL) and dried over anhydrous Na_2_SO_4_. Upon completion of drying, the resulting solution was filtered and concentrated under reduced pressure. The residue obtained was then purified by silica gel column chromatography (petroleum ether/ethyl acetate = 1/2), yielding the intermediate **21** (279 mg, 83%) as a white solid. Melting point: 106–107 °C; ^1^H NMR (400 MHz, DMSO-*d*_6_) δ 8.44 (d, *J* = 8.8 Hz, 2H), 8.01 (d, *J* = 8.8 Hz, 2H), 3.41 (t, *J* = 5.1 Hz, 4H), 2.96 (t, *J* = 5.0 Hz, 4H), 1.34 (s, 9H). HRMS (ESI) *m*/*z* calculated for C_15_H_21_N_3_O_6_S [M + H]^+^ 372.1229, found 372.1223.

#### 3.1.2. *tert*-Butyl 4-((4-Nitrobenzyl)sulfonyl)piperazine-1-carboxylate (Compound **22**)

Compound **22** was synthesized using (4-nitrophenyl)methanesulfonyl chloride as a key starting material, following a procedure similar to that employed for the synthesis of compound **21**. White solid; yield: 18%; melting point: 133–134 °C; ^1^H NMR (400 MHz, DMSO-*d*_6_) δ 8.26 (d, *J* = 8.7 Hz, 2H), 7.69 (d, *J* = 8.8 Hz, 2H), 4.65 (s, 2H), 3.36 (t, *J* = 5.1 Hz, 4H), 3.13 (t, *J* = 5.0 Hz, 4H), 1.41 (s, 9H). HRMS (ESI) *m*/*z* calculated for C_16_H_23_N_3_O_6_S [M − H]^−^ 384.1234, found 384.1238.

#### 3.1.3. *tert*-Butyl 4-((4-Aminophenyl)sulfonyl)piperazine-1-carboxylate (Compound **23**)

To a solution of **21** (2.8 g, 0.7 mmol) in methanol (10 mL), Pd/C (0.28 g, 10% *w*/*w*) was added. The mixture was stirred at 25 °C for 8 h under a H_2_ atmosphere (1 atm). After completion of the reaction, the solution was filtered and concentrated under reduced pressure. The crude mixture was then subjected to purification by silica gel column chromatography (dichloromethane/methanol = 40/1), affording intermediate **23** (2.36 g, 92%) as a white solid. Melting point: 180–181 °C; ^1^H NMR (400 MHz, DMSO-*d*_6_) δ 8.91 (d, *J* = 141.3 Hz, 2H), 7.49 (d, *J* = 8.1 Hz, 2H), 6.93 (s, 2H), 3.38 (s, 4H), 2.77 (s, 4H), 1.35 (s, 9H). HRMS (ESI) *m*/*z* calculated for C_15_H_23_N_3_O_4_S [M + Na]^+^ 364.1307, found 364.1300.

#### 3.1.4. *tert*-Butyl 4-((4-Aminobenzyl)sulfonyl)piperazine-1-carboxylate (Compound **24**)

Compound **24** was synthesized using compound **22** as a key starting material, following a procedure similar to that employed for the synthesis of compound **23**. White solid; yield: 53%; melting point: 133–134 °C; ^1^H NMR (400 MHz, DMSO-*d*_6_) δ 7.02 (d, *J* = 8.4 Hz, 2H), 6.53 (d, *J* = 8.4 Hz, 2H), 5.19 (s, 2H), 4.18 (s, 2H), 3.30 (t, *J* = 5.1 Hz, 4H), 3.11–2.87 (m, 4H), 1.40 (s, 9H). HRMS (ESI) *m*/*z* calculated for C_16_H_25_N_3_O_4_S [M + Na]^+^ 378.1463, found 378.1459.

#### 3.1.5. *tert*-Butyl 4-((4-(3-(Pyridin-3-ylmethyl)thioureido)phenyl)sulfonyl)piperazine-1-carboxylate (Compound **25**)

To a solution of NaHCO_3_ (0.15 g, 1.8 mmol) in water (5 mL), **23** (0.34 g, 0.9 mmol) and thiophosgene (0.13 g, 1.1 mmol) were added and stirred at 25 °C for 10 min. Dichloromethane (5 mL) was then added to the reaction mixture, and stirring continued at 25 °C for 2 h. The resulting mixture was further diluted with dichloromethane (15 mL), and the organic layer was washed with water (30 mL) and brine (30 mL), and dried over anhydrous Na_2_SO_4_. Upon completion of drying, the solution was filtered and concentrated under reduced pressure. The residue obtained was purified by silica gel column chromatography (dichloromethane/methanol = 40/1), providing intermediate **25** (0.33 g, 76%) as a white solid. Melting point: 127–128 °C; ^1^H NMR (400 MHz, DMSO-*d*_6_) δ 10.13 (s, 1H), 8.62 (t, *J* = 5.8 Hz, 1H), 8.58 (d, *J* = 2.2 Hz, 1H), 8.48 (d, *J* = 3.1 Hz, 1H), 7.81 (d, *J* = 8.8 Hz, 2H), 7.77 (d, *J* = 7.9 Hz, 1H), 7.66 (d, *J* = 8.9 Hz, 2H), 7.42–7.33 (m, 1H), 4.79 (d, *J* = 5.6 Hz, 2H), 3.40 (t, *J* = 5.0 Hz, 4H), 2.84 (t, *J* = 5.0 Hz, 4H), 1.34 (s, 9H). HRMS (ESI) *m*/*z* calculated for C_22_H_29_N_5_O_4_S_2_ [M + H]^+^ 492.1734, found 492.1738.

#### 3.1.6. *tert*-Butyl 4-((4-(3-(Pyridin-3-ylmethyl)ureido)phenyl)sulfonyl)piperazine-1-carboxylate (Compound **26**)

Compound **26** was synthesized using compound **23** as a key intermediate, following a procedure similar to that employed for the synthesis of compound **25**. White solid; yield: 33%; melting point: 151–152 °C; ^1^H NMR (400 MHz, DMSO-*d*_6_) δ 9.21 (s, 1H), 8.54 (d, *J* = 2.2 Hz, 1H), 8.46 (d, *J* = 4.8 Hz, 1H), 7.72 (d, *J* = 7.8 Hz, 1H), 7.66 (d, *J* = 6.8 Hz, 1H), 7.58 (d, *J* = 8.9 Hz, 2H), 7.40–7.32 (m, 1H), 6.92 (t, *J* = 6.0 Hz, 1H), 4.35 (d, *J* = 5.9 Hz, 2H), 3.38 (t, *J* = 5.0 Hz, 4H), 2.80 (t, *J* = 5.0 Hz, 4H), 1.34 (s, 9H). HRMS (ESI) *m*/*z* calculated for C_22_H_29_N_5_O_5_S [M + H]^+^ 476.1962, found 476.1975.

#### 3.1.7. *tert*-Butyl 4-((4-(3-(Pyridin-3-ylmethyl)ureido)benzyl)sulfonyl)piperazine-1-carboxylate (Compound **27**)

Compound **27** was synthesized using compound **24** as a key intermediate, following a procedure similar to that employed for the synthesis of compound **25**. Yellow solid; yield: 50%; melting point: 141–142 °C; ^1^H NMR (400 MHz, DMSO-*d*_6_) δ 8.72 (s, 1H), 8.50 (d, *J* = 27.2 Hz, 2H), 7.72 (d, *J* = 9.8 Hz, 1H), 7.41 (d, *J* = 8.5 Hz, 2H), 7.39–7.34 (m, 1H), 7.25 (d, *J* = 8.3 Hz, 2H), 6.74 (t, *J* = 6.0 Hz, 1H), 4.32 (d, *J* = 5.8 Hz, 4H), 3.43–3.25 (m, 4H), 3.05 (t, *J* = 5.0 Hz, 4H), 1.40 (s, 9H). HRMS (ESI) *m*/*z* calculated for C_23_H_31_N_5_O_5_S [M + H]^+^ 490.2119, found 490.2126.

#### 3.1.8. 1-(4-(Piperazin-1-ylsulfonyl)phenyl)-3-(pyridin-3-ylmethyl)thiourea (Compound **28**)

To a solution of **25** (0.33 g, 0.67 mmol) in dichloromethane (10 mL), trifluoroacetic acid (1.50 g, 13.4 mmol) was added and stirred at 25 °C for 4 h under a nitrogen atmosphere. The reaction mixture was washed with saturated NaHCO_3_ (60 mL) and brine (30 mL) and dried over anhydrous Na_2_SO_4_. After completion of drying, the resulting solution was filtered and concentrated under reduced pressure to yield intermediate **28** (0.17 g, 65%) as a white solid without further purification. Melting point: 84–85 °C; ^1^H NMR (400 MHz, DMSO-*d*_6_) δ 8.96 (t, *J* = 5.8 Hz, 1H), 8.72 (s, 1H), 8.57 (d, *J* = 2.2 Hz, 1H), 8.48 (d, *J* = 6.4 Hz, 1H), 7.91 (d, *J* = 8.5 Hz, 2H), 7.76 (d, *J* = 7.9 Hz, 1H), 7.70 (d, *J* = 8.6 Hz, 2H), 7.46–7.29 (m, 1H),4.80 (d, *J* = 5.6 Hz, 2H), 3.34 (s, 1H), 3.20 (t, *J* = 5.0 Hz, 4H), 3.10 (d, *J* = 2.7 Hz, 4H). HRMS (ESI) *m*/*z* calculated for C_17_H_21_N_5_O_2_S_2_ [M + H]^+^ 392.1209, found 392.1203.

#### 3.1.9. 1-(4-(Piperazin-1-ylsulfonyl)phenyl)-3-(pyridin-3-ylmethyl)urea (Compound **29**)

Compound **29** was synthesized using compound **26** as a key intermediate, following a procedure similar to that employed for the synthesis of compound **28**. White solid; yield: 74%; melting point: 102–103 °C; ^1^H NMR (400 MHz, DMSO-*d*_6_) δ 9.23 (d, *J* = 2.5 Hz, 1H), 8.53 (d, *J* = 2.2 Hz, 1H), 8.46 (d, *J* = 6.4 Hz, 1H), 7.71 (d, *J* = 7.8 Hz, 1H), 7.65 (d, *J* = 8.9 Hz, 2H), 7.57 (d, *J* = 8.9 Hz, 2H), 7.39–7.35 (m, 1H), 6.96 (t, *J* = 4.3 Hz, 1H), 4.34 (d, *J* = 5.8 Hz, 2H), 3.16 (s, 1H), 2.73 (d, *J* = 7.7 Hz, 4H), 2.70 (d, *J* = 4.8 Hz, 4H). HRMS (ESI) *m*/*z* calculated for C_17_H_21_N_5_O_3_S [M + H]^+^ 376.1438, found 376.1434.

#### 3.1.10. 1-(4-((Piperazin-1-ylsulfonyl)methyl)phenyl)-3-(pyridin-3-ylmethyl)urea (Compound **30**)

Compound **30** was synthesized using compound **27** as a key intermediate, following a procedure similar to that employed for the synthesis of compound **28**. White solid; yield: 85%; melting point: 158–159 °C; ^1^H NMR (400 MHz, DMSO-*d*_6_) δ 9.51 (s, 1H), 9.41 (s, 2H), 8.84 (d, *J* = 2.0 Hz, 1H), 8.81 (d, *J* = 4.3 Hz, 2H), 8.52–8.45 (m, 1H), 8.06–7.98 (m, 1H), 7.44 (d, *J* = 8.6 Hz, 2H), 7.38 (t, *J* = 6.0 Hz, 1H), 7.28 (d, *J* = 8.6 Hz, 2H), 4.50 (d, *J* = 5.3 Hz, 2H), 4.43 (s, 2H), 3.32 (d, *J* = 2.7 Hz, 4H), 3.04 (s, 4H). HRMS (ESI) *m*/*z* calculated for C_18_H_23_N_5_O_3_S [M + H]^+^ 390.1594, found 390.1589.

#### 3.1.11. 1-(4-((4-(2-Fluoro-5-((4-oxo-3,4-dihydrophthalazin-1-yl)methyl)benzoyl)piperazin-1-yl)sulfonyl)phenyl)-3-(pyridin-3-ylmethyl)thiourea (Compound **DDY01**)

To a solution of 2-fluoro-5-((4-oxo-3,4-dihydrophthalazin-1-yl)methyl)benzoic acid (0.24 g, 0.8 mmol) in N,N-dimethylformamide (5 mL), EDCI (0.16 g, 0.8 mmol), HOBt (0.12 g, 0.8 mmol), and DIPEA (0.23 mL, 1.38 mmol) were added. After stirring at 25 °C for 2 h, **28** (0.31 g, 0.8 mmol) was introduced into the reaction mixture. The reaction mixture was stirred at 25 °C for 12 h, and then poured into dichloromethane (20 mL). The organic layer was washed with water (30 mL) and brine (30 mL), and dried over anhydrous Na_2_SO_4_. Upon completion of drying, the resulting solution was filtered and concentrated under reduced pressure. The residue obtained was purified by silica gel column chromatography (dichloromethane/methanol = 40/1), yielding the compound **DDY01** (279 mg, 83%) as a white solid. Melting point: 189–190 °C. IR (neat) νmax 3038, 1650, 1523, 1328, 1159, 935, 841, 704 cm^−1^. ^1^H NMR (400 MHz, DMSO-*d*_6_) δ 12.57 (s, 1H), 10.13 (s, 1H), 8.63 (t, *J* = 5.7 Hz, 1H), 8.58 (d, *J* = 2.2 Hz, 1H), 8.48 (d, *J* = 3.1 Hz, 1H), 8.27 (d, *J* = 7.5 Hz, 1H), 7.97–7.90 (m, 1H), 7.91–7.79 (m, 4H), 7.77 (d, *J* = 7.9 Hz, 1H), 7.69–7.62 (m, 2H), 7.47–7.35 (m, 2H), 7.31 (d, *J* = 6.5 Hz, 1H), 7.23–7.15 (m, 1H), 4.79 (d, *J* = 5.6 Hz, 2H), 4.29 (s, 2H), 3.70 (t, *J* = 5.2 Hz, 2H), 3.26 (d, *J* = 5.3 Hz, 2H), 2.97 (d, *J* = 5.4 Hz, 2H), 2.83 (d, *J* = 4.5 Hz, 2H). ^13^C NMR (101 MHz, DMSO-*d*_6_) δ 181.18, 164.40, 159.85, 156.76 (d, *J* = 244.4 Hz), 149.42, 148.69, 145.28, 144.69, 135.76, 135.30 (d, *J* = 3.3 Hz), 134.61, 133.94, 132.35, 132.27, 132.02, 129.55, 129.17, 128.84, 128.35, 126.53, 125.90, 123.90, 123.62 (d, *J* = 18.2 Hz), 121.91, 116.33 (d, *J* = 21.6 Hz), 46.10, 45.18, 41.04, 36.85. HRMS (ESI) *m*/*z* calculated for C_33_H_30_FN_7_O_4_S_2_ [M + H]^+^ 672.1857, found 672.1858.

#### 3.1.12. 1-(4-((4-(2-Fluoro-5-((4-oxo-3,4-dihydrophthalazin-1-yl)methyl)benzoyl)piperazin-1-yl)sulfonyl)phenyl)-3-(pyridin-3-ylmethyl)urea (Compound **DDY02**)

Compound **DDY02** was synthesized using compound **29** as a key intermediate, following a procedure similar to that employed for the synthesis of compound **DDY01**. White solid; yield: 33%; melting point: 197–198 °C. IR (neat) νmax 2988, 2905, 1698, 1645, 1538, 1151, 837, 721 cm^−1^. ^1^H NMR (400 MHz, DMSO-*d*_6_) δ 12.57 (s, 1H), 9.28 (s, 1H), 8.56 (d, *J* = 2.2 Hz, 1H), 8.48 (d, *J* = 3.2 Hz, 1H), 8.34–8.23 (m, 1H), 7.97–7.91 (m, 1H), 7.90–7.80 (m, 2H), 7.76 (d, *J* = 7.9 Hz, 1H), 7.70–7.64 (m, 2H), 7.63–7.56 (m, 2H), 7.46–7.35 (m, 2H), 7.32 (d, *J* = 6.5 Hz, 1H), 7.19 (t, *J* = 9.0 Hz, 1H), 6.98 (t, *J* = 6.0 Hz, 1H), 4.36 (d, *J* = 5.9 Hz, 2H), 4.30 (s, 2H), 3.70 (t, *J* = 5.1 Hz, 2H), 3.26 (t, *J* = 5.0 Hz, 2H), 2.93 (t, *J* = 5.2 Hz, 2H), 2.80 (t, *J* = 5.1 Hz, 2H). ^13^C NMR (101 MHz, DMSO-*d*_6_) δ 164.38, 159.84, 156.75 (d, *J* = 244.7 Hz), 155.30, 148.79, 148.18, 145.53, 145.28, 136.24, 135.92, 135.31, 133.92, 132.29 (d, *J* = 8.4 Hz), 132.02, 129.55, 129.32, 128.35, 126.57, 126.53, 125.90, 124.09, 123.73, 123.55, 117.78, 116.31 (d, *J* = 21.5 Hz), 49.07, 46.11, 40.96, 36.84. HRMS (ESI) *m*/*z* calculated for C_33_H_30_FN_7_O_5_S [M + H]^+^ 656.2086, found 656.2087.

#### 3.1.13. 1-(4-((4-(3-(4-Oxo-3,4-dihydroquinazolin-2-yl)propanoyl)piperazin-1-yl)sulfonyl)phenyl)-3-(pyridin-3-ylmethyl)urea (Compound **DDY03**)

Compound **DDY03** was synthesized using compound **29** as a key intermediate, following a procedure similar to that employed for the synthesis of compound **DDY01**. White solid; yield: 55%; melting point: 120–121 °C. IR (neat) νmax 2979, 2893, 1707, 1668, 1618, 1538, 1159, 940, 781, 730 cm^−1^. ^1^H NMR (400 MHz, DMSO-*d*_6_) δ 12.12 (s, 1H), 9.42 (s, 1H), 8.55 (s, 1H), 8.47 (d, *J* = 3.2 Hz, 1H), 8.04 (d, *J* = 9.5 Hz, 1H), 7.76–7.65 (m, 4H), 7.60 (d, *J* = 8.6 Hz, 2H), 7.42–7.32 (m, 2H), 7.29 (d, *J* = 8.1 Hz, 1H), 7.06 (t, *J* = 6.0 Hz, 1H), 4.36 (d, *J* = 5.9 Hz, 2H), 3.69–3.59 (m, 2H), 3.53 (t, *J* = 5.2 Hz, 2H), 2.92 (t, *J* = 4.8 Hz, 2H), 2.84–2.71 (m, 6H). ^13^C NMR (101 MHz, DMSO-*d*_6_) δ 170.05, 162.02, 157.16, 155.34, 149.19, 149.12, 148.55, 145.57, 136.04, 135.43, 134.65, 129.37, 126.99, 126.35, 126.27, 126.13, 123.92, 121.29, 117.64, 46.62, 46.34, 44.70, 29.82, 28.76. HRMS (ESI) *m*/*z* calculated for C_28_H_29_N_7_O_5_S [M + H]^+^ 576.2024, found 576.2022.

#### 3.1.14. 2-((4-Fluoro-3-(4-((4-(3-(pyridin-3-ylmethyl)thioureido)phenyl)sulfonyl)piperazine-1-carbonyl)benzyl)oxy)benzamide (Compound **DDY04**)

Compound **DDY04** was synthesized using compound **28** as a key intermediate, following a procedure similar to that employed for the synthesis of compound **DDY01**. White solid; yield: 86%; melting point: 125–126 °C. IR (neat) νmax 3032, 1650, 1541, 1319, 1224, 1156, 940, 831, 710 cm^−1^. ^1^H NMR (400 MHz, DMSO-*d*_6_) δ 10.42 (s, 1H), 8.85 (s, 1H), 8.57 (s, 1H), 8.47 (d, *J* = 3.3 Hz, 1H), 7.87 (d, *J* = 8.6 Hz, 2H), 7.75 (d, *J* = 5.9 Hz, 2H), 7.66 (d, *J* = 8.7 Hz, 2H), 7.64–7.60 (m, 1H), 7.55 (s, 1H), 7.53–7.48 (m, 1H), 7.46–7.40 (m, 1H), 7.37 (d, *J* = 7.8, 4.8 Hz, 1H), 7.32 (t, *J* = 9.0 Hz, 1H), 7.16 (d, *J* = 8.3 Hz, 1H), 7.03 (t, *J* = 7.5 Hz, 1H), 5.21 (s, 2H), 4.80 (s, 2H), 3.74 (t, *J* = 5.1 Hz, 2H), 3.33 (s, 2H), 2.99 (t, *J* = 5.0 Hz, 2H), 2.88 (t, *J* = 5.1 Hz, 2H). ^13^C NMR (101 MHz, DMSO-*d*_6_) δ 181.16, 167.04, 164.23, 157.67 (d, *J* = 246.3 Hz), 156.27, 149.42, 148.69, 144.69, 135.76, 134.61, 133.95 (d, *J* = 3.2 Hz), 132.57, 131.63 (d, *J* = 8.3 Hz), 131.01, 129.10, 128.87, 127.80, 124.34, 123.91, 123.75, 121.91, 121.33, 116.57 (d, *J* = 21.8 Hz), 113.94, 69.34, 46.50, 46.33, 46.16. HRMS (ESI) *m*/*z* calculated for C_32_H_31_FN_6_O_6_S_2_ [M + H]^+^ 663.1854, found 663.1865.

#### 3.1.15. 2-((4-Fluoro-3-(4-((4-(3-(pyridin-3-ylmethyl)ureido)phenyl)sulfonyl)piperazine-1-carbonyl)benzyl)oxy)benzamide (Compound **DDY05**)

Compound **DDY05** was synthesized using compound **29** as a key intermediate, following a procedure similar to that employed for the synthesis of compound **DDY01**. White solid; yield: 81%; melting point: 119–120 °C. IR (neat) νmax 3401, 1650, 1535, 1221, 1156, 1100, 940, 834, 730 cm^−1^. ^1^H NMR (400 MHz, DMSO-*d*_6_) δ 9.26 (s, 1H), 8.64–8.44 (m, 2H), 7.74 (t, *J* = 7.1 Hz, 2H), 7.63 (d, *J* = 16.6 Hz, 5H), 7.57–7.46 (m, 3H), 7.47–7.35 (m, 2H), 7.31 (t, *J* = 8.9 Hz, 1H), 7.17 (d, *J* = 8.2 Hz, 1H), 7.04 (t, *J* = 7.5 Hz, 1H), 6.96 (d, *J* = 6.2 Hz, 1H), 5.21 (s, 2H), 4.35 (d, *J* = 5.8 Hz, 2H), 3.73 (s, 2H), 3.42 (s, 2H), 2.95 (s, 2H), 2.84 (s, 2H). ^13^C NMR (101 MHz, DMSO-*d*_6_) δ 167.03, 164.21, 157.67 (d, *J* = 246.2 Hz), 156.28, 155.28, 148.97, 148.35, 145.54, 136.14, 135.72, 133.95 (d, *J* = 3.2 Hz), 132.55, 131.61 (d, *J* = 8.5 Hz), 131.01, 129.34, 128.92, 126.56, 124.36, 124.03, 123.87 (d, *J* = 18.2 Hz), 121.33, 117.79, 116.55 (d, *J* = 21.9 Hz), 113.95, 69.37, 46.50, 46.31, 46.16. HRMS (ESI) *m*/*z* calculated for C_32_H_31_FN_6_O_6_S [M + H]^+^ 647.2083, found 647.2078.

#### 3.1.16. 2-((4-Fluoro-3-(4-((4-(3-(pyridin-3-ylmethyl)ureido)benzyl)sulfonyl)piperazine-1-carbonyl)benzyl)oxy)benzamide (Compound **DDY06**)

Compound **DDY06** was synthesized using compound **30** as a key intermediate, following a procedure similar to that employed for the synthesis of compound **DDY01**. White solid; yield: 37%; melting point: 146–147 °C. IR (neat) νmax 3039, 1647, 1544, 1511, 1313, 1226, 1150, 943, 840, 754 cm^−1^. ^1^H NMR (400 MHz, DMSO-*d*_6_) δ 8.73 (s, 1H), 8.53 (d, *J* = 2.2 Hz, 1H), 8.45 (d, *J* = 3.0 Hz, 1H), 7.75 (d, *J* = 5.8 Hz, 1H), 7.71 (d, *J* = 7.8 Hz, 1H), 7.65 (t, *J* = 8.0 Hz, 1H), 7.59 (s, 1H), 7.55 (d, *J* = 6.5 Hz, 2H), 7.45–7.40 (m, 3H), 7.38–7.31 (m, 2H), 7.26 (d, *J* = 8.6 Hz, 2H), 7.19 (d, *J* = 8.3 Hz, 1H), 7.04 (t, *J* = 7.3 Hz, 1H), 6.75 (t, *J* = 6.0 Hz, 1H), 5.26 (s, 2H), 4.42–4.26 (m, 4H), 3.67 (t, *J* = 5.2 Hz, 2H), 3.21 (d, *J* = 15.3 Hz, 4H), 3.08 (s, 2H). ^13^C NMR (101 MHz, DMSO-*d*_6_) δ 167.13, 164.32, 157.70 (d, *J* = 246.2 Hz), 156.23, 155.64, 149.20, 148.50, 140.97, 136.29, 135.46, 134.00, 132.55, 131.75, 131.54 (d, *J* = 8.1 Hz), 130.98, 128.64, 124.47, 124.06 (d, *J* = 18.4 Hz), 123.92, 121.89, 121.34, 118.01, 116.64 (d, *J* = 21.9 Hz), 113.94, 69.32, 55.38, 47.02, 46.08, 45.76. HRMS (ESI) *m*/*z* calculated for C_33_H_33_FN_6_O_6_S [M + H]^+^ 661.2239, found 661.2234.

#### 3.1.17. 5-Fluoro-2-((4-fluoro-3-(4-((4-(3-(pyridin-3-ylmethyl)ureido)phenyl)sulfonyl)piperazine-1-carbonyl)benzyl)oxy)benzamide (Compound **DDY07**)

Compound **DDY07** was synthesized using compound **29** as a key intermediate, following a procedure similar to that employed for the synthesis of compound **DDY01**. White solid; yield: 76%; melting point: 122–123 °C. IR (neat) νmax 3420, 3174, 1644, 1544, 1434, 1233, 1156, 926, 828, 727 cm^−1^. ^1^H NMR (400 MHz, DMSO-*d*_6_) δ 9.23 (s, 1H), 8.54 (d, *J* = 2.2 Hz, 1H), 8.46 (d, *J* = 4.8 Hz, 1H), 7.72 (d, *J* = 7.8 Hz, 1H), 7.69–7.63 (m, 4H), 7.60 (d, *J* = 8.9 Hz, 3H), 7.52–7.46 (m, 2H), 7.38–7.34 (m, 1H), 7.34–7.30 (m, 1H), 7.30–7.27 (m, 1H), 7.21 (d, *J* = 4.4 Hz, 1H), 6.94 (t, *J* = 6.0 Hz, 1H), 5.20 (s, 2H), 4.35 (d, *J* = 5.9 Hz, 2H), 3.73 (t, *J* = 5.2 Hz, 2H), 3.30 (t, *J* = 5.0 Hz, 2H), 2.95 (t, *J* = 5.1 Hz, 2H), 2.84 (s, 2H). ^13^C NMR (101 MHz, DMSO-*d*_6_) δ 165.79, 164.18, 157.69 (d, *J* = 246.3 Hz), 156.63 (d, *J* = 237.6 Hz), 155.26, 152.58 (d, *J* = 2.0 Hz), 149.19, 148.56, 145.55, 136.01, 135.46, 133.77 (d, *J* = 3.4 Hz), 131.66 (d, *J* = 8.2 Hz), 129.34, 128.97, 126.50, 125.96 (d, *J* = 6.5 Hz), 123.93, 123.88 (d, *J* = 18.4 Hz), 118.79 (d, *J* = 23.1 Hz), 117.78, 116.93 (d, *J* = 24.4 Hz), 116.56 (d, *J* = 22.0 Hz), 115.93 (d, *J* = 7.8 Hz), 70.04, 46.49, 46.16, 40.98. HRMS (ESI) *m*/*z* calculated for C_32_H_39_F_2_N_6_O_6_S [M + H]^+^ 665.1988, found 665.1984.

#### 3.1.18. 2-((3-(4-((4-(3-(Pyridin-3-ylmethyl)ureido)phenyl)sulfonyl)piperazine-1-carbonyl)benzyl)oxy)benzamide (Compound **DDY08**)

Compound **DDY08** was synthesized using compound **29** as a key intermediate, following a procedure similar to that employed for the synthesis of compound **DDY01**. White solid; yield: 81%; melting point: 202–203 °C. IR (neat) νmax 3325, 3041, 1698, 1647, 1588, 1538, 1213, 1153, 1106, 937, 831, 730 cm^−1^. ^1^H NMR (400 MHz, DMSO-*d*_6_) δ 9.22 (s, 1H), 8.54 (d, *J* = 2.2 Hz, 1H), 8.46 (d, *J* = 3.1 Hz, 1H), 7.77 (d, *J* = 7.7 Hz, 1H), 7.75–7.68 (m, 1H), 7.69–7.62 (m, 2H), 7.63–7.54 (m, 4H), 7.54–7.49 (m, 1H), 7.47 (d, *J* = 2.4 Hz, 1H), 7.48–7.39 (m, 2H), 7.40–7.33 (m, 1H), 7.36–7.29 (m, 1H), 7.17 (d, *J* = 8.8 Hz, 1H), 7.07–6.99 (m, 1H), 6.94 (t, *J* = 6.0 Hz, 1H), 5.26 (s, 2H), 4.35 (d, *J* = 5.8 Hz, 2H), 3.69 (s, 2H), 3.46 (s, 2H), 2.90 (s, 4H). ^13^C NMR (101 MHz, DMSO-*d*_6_) δ 169.38, 167.00, 156.38, 155.28, 149.17, 148.54, 145.49, 137.38, 136.04, 135.92, 135.47, 132.61, 131.06, 129.48, 129.34, 129.22, 127.33, 126.75, 126.64, 124.16, 123.94, 121.26, 117.79, 113.89, 69.94, 55.39, 46.22, 40.98. HRMS (ESI) *m*/*z* calculated for C_32_H_32_N_6_O_6_S [M + H]^+^ 629.2177, found 629.2176.

#### 3.1.19. 5-Fluoro-2-((3-(4-((4-(3-(pyridin-3-ylmethyl)ureido)phenyl)sulfonyl)piperazine-1-carbonyl)benzyl)oxy)benzamide (Compound **DDY09**)

Compound **DDY09** was synthesized using compound **29** as a key intermediate, following a procedure similar to that employed for the synthesis of compound **DDY01**. White solid; yield: 78%; melting point: 147–148 °C. IR (neat) νmax 3423, 3062, 1665, 1428, 1260, 1159, 822, 727 cm^−1^. ^1^H NMR (400 MHz, DMSO-*d*_6_) δ 9.22 (s, 1H), 8.54 (d, *J* = 2.2 Hz, 1H), 8.46 (d, *J* = 6.4 Hz, 1H), 7.71 (d, *J* = 7.9 Hz, 1H), 7.69–7.63 (m, 4H), 7.59 (d, *J* = 9.0 Hz, 2H), 7.58–7.54 (m, 1H), 7.51 (d, *J* = 6.0 Hz, 1H), 7.48–7.42 (m, 2H), 7.39–7.34 (m, 1H), 7.34–7.27 (m, 2H), 7.22 (d, *J* = 4.3 Hz, 1H), 6.93 (t, *J* = 6.0 Hz, 1H),5.24 (s, 2H), 4.35 (d, *J* = 5.9 Hz, 2H), 3.78–3.57 (m, 2H), 3.40 (s, 2H), 3.00–2.83 (m, 4H). ^13^C NMR (101 MHz, DMSO-*d*_6_) δ 169.37, 165.75, 162.78, 156.59 (d, *J* = 237.4 Hz), 155.27, 152.69 (d, *J* = 1.9 Hz), 149.19, 148.56, 145.50, 137.21, 135.98 (d, *J* = 8.3 Hz), 135.45, 129.51, 129.33, 129.23, 127.39, 126.79, 126.65, 125.77 (d, *J* = 6.5 Hz), 123.93, 118.85 (d, *J* = 23.0 Hz), 117.79, 116.96 (d, *J* = 24.4 Hz), 115.85 (d, *J* = 7.8 Hz), 70.62, 46.85, 46.23, 40.98. HRMS (ESI) *m*/*z* calculated for C_32_H_31_FN_6_O_6_S [M + H]^+^ 647.2083, found 647.2074.

#### 3.1.20. 2-(4-((4-(3-(Pyridin-3-ylmethyl)thioureido)phenyl)sulfonyl)piperazine-1-carbonyl)-1H-benzo[d]imidazole-4-carboxamide (Compound **DDY10**)

Compound **DDY10** was synthesized using compound **28** as a key intermediate, following a procedure similar to that employed for the synthesis of compound **DDY01**. Yellow solid; yield: 23%; melting point: >250 °C. IR (neat) νmax 3304, 3227, 1671, 1621, 1494, 1423, 1307, 1239, 1165, 937, 739 cm^−1^. ^1^H NMR (400 MHz, DMSO-*d*_6_) δ 13.58 (s, 1H), 10.12 (s, 1H), 8.68 (s, 1H), 8.64–8.59 (m, 1H), 8.57 (d, *J* = 2.2 Hz, 1H), 8.48 (d, *J* = 4.8 Hz, 1H), 7.93 (d, *J* = 7.3 Hz, 1H), 7.81 (d, *J* = 8.7 Hz, 2H), 7.77 (d, *J* = 5.8 Hz, 2H), 7.69 (d, *J* = 8.7 Hz, 3H), 7.44 (t, *J* = 7.6 Hz, 1H), 7.38 (dd, *J* = 7.9, 4.7 Hz, 1H), 4.78 (d, *J* = 5.6 Hz, 2H), 4.36 (s, 2H), 3.83 (s, 2H), 3.21–2.94 (m, 4H). ^13^C NMR (151 MHz, DMSO-*d*_6_) δ 181.14, 166.02, 158.54, 149.27, 148.55, 145.77, 144.63, 140.10, 135.90, 134.68, 134.08, 128.93, 124.52, 124.43, 124.25, 123.95, 121.94, 118.87, 116.49, 46.72, 46.20, 45.18. HRMS (ESI) *m*/*z* calculated for C_26_H_26_N_8_O_4_S_2_ [M + H]^+^ 579.1591, found 579.1596.

#### 3.1.21. 2-(4-((4-(3-(Pyridin-3-ylmethyl)ureido)phenyl)sulfonyl)piperazine-1-carbonyl)-1H-benzo[d]imidazole-4-carboxamide (Compound **DDY11**)

Compound **DDY11** was synthesized using compound **29** as a key intermediate, following a procedure similar to that employed for the synthesis of compound **DDY01**. Yellow solid; yield: 41%; melting point: >250 °C. IR (neat) νmax 3479, 3242, 1668, 1624, 1585, 1494, 1245, 1159, 937, 733 cm^−1^. ^1^H NMR (400 MHz, DMSO-*d*_6_) δ 13.57 (s, 1H), 9.24 (s, 1H), 8.68 (s, 1H), 8.61–8.49 (m, 1H), 8.46 (d, *J* = 4.8 Hz, 1H), 7.92 (d, *J* = 7.5 Hz, 1H), 7.73 (d, *J* = 8.2 Hz, 3H), 7.63 (q, *J* = 8.8 Hz, 4H), 7.43 (s, 1H), 7.41–7.33 (m, 1H), 6.93 (t, *J* = 6.1 Hz, 1H), 4.34 (d, *J* = 6.0 Hz, 4H), 3.81 (s, 2H), 3.02 (s, 4H). ^13^C NMR (101 MHz, DMSO-*d*_6_) δ 166.04, 158.54, 155.27, 148.89, 148.28, 145.80, 145.54, 140.11, 136.17, 135.77, 134.09, 129.38, 126.39, 124.46, 124.23, 124.04, 117.77, 116.50, 46.69, 46.18, 42.11. HRMS (ESI) *m*/*z* calculated for C_26_H_26_N_8_O_5_S [M + H]^+^ 563.1820, found 563.1838.

### 3.2. Molecule Docking

The crystal structures of NAMPT (PDB ID: 4JR5) and PARP1 (PDB ID: 5DS3) were obtained from the Protein Data Bank. Molecule docking was performed using BIOVIA Discovery Studio 2016 (San Diego, CA, USA). Prior to docking, the protein structures of NAMPT and PARP1 underwent processing using the Protein Preparation module. For PARP1 protein processing, default parameters of the Protein Preparation module were applied. In contrast to PARP1, the “Keep Water” parameter of the Protein Preparation module was set to selected for NAMPT protein processing, preserving some conserved waters. Binding sites were defined as spherical regions with a 15 Å diameter, centered on the ligand molecules using the From Current Selection module.

The poses of compounds were subjected to minimization using the Full Minimization module based on the CHARMm forcefield. Subsequently, docking simulations were carried out utilizing the CDOCKER module, with parameters “Pose Cluster Radius” and “Orientations to Refine” set to 0.5 and 20, respectively. The best 10 poses for each compound were retained, and the pose with the most favorable interactions was selected for further analysis using the module “Display receptor-ligand interactions”. The graphical display was processed using PyMol (version 2.5).

### 3.3. Determination of Inhibition Rates and IC_50_ Values against NAMPT and PARP1

The inhibitory effects of the target compounds on NAMPT and PARP1 were evaluated at Huawei Pharmaceutical Co., Ltd. (Jinan, China). Dilutions ten-fold higher than the final compound concentrations were prepared with 10% DMSO, and 5 µL of the dilution was added to a 50 µL reaction, resulting in a final DMSO concentration of 1% in all reactions. All enzymatic reactions were conducted in duplicates at 30 °C for 90 min in a 50 µL mixture containing 50 mM Tris-HCl, pH 8.0, 12.5 mM MgCl_2_, 20 µM nicotinamide, 0.4 mM Phosphoribosyl pyrophosphate, 2 mM ATP, 30 µg/mL of alcohol dehydrogenase, 10 µg/mL of NMNAT, 1.5% alcohol, 1 mM DTT, 0.02% BSA, 0.01% Tween 20, and the test compound. Fluorescence intensity was measured at an excitation of 360 nm and an emission of 460 nm using a Tecan Infinite M1000 microplate reader. NAMPT activity assays were performed in duplicates at each concentration. The fluorescent intensity data were analyzed using GraphPad Prism software (version 8.3.0). In the absence of the compound, the fluorescent intensity (Ft) in each dataset was defined as 100% activity, and in the absence of NAMPT, the fluorescent intensity (Fb) in each dataset was defined as 0% activity. The percent activity in the presence of each compound was calculated using the formula %activity = (F − Fb)/(Ft − Fb), where F is the fluorescent intensity in the presence of the compound. Following the described method, the inhibition rates of **DDY01**–**DDY11** against NAMPT were initially determined at a concentration of 0.1 µM. Subsequently, the inhibition rates of some selected compounds at nine concentrations (0.01 nM, 0.03 nM, 0.1 nM, 0.33 nM, 1 nM, 3.3 nM, 10 nM, 33 nM, 100 nM) were individually tested and obtained. IC_50_ values were determined using non-linear regression analysis in Graphpad Prism, employing concentration–response data.

The inhibition of PARP1 enzymatic activity by the tested compounds was assessed using an ELISA in 96-well plates. Each well was pre-coated with histone (20 μg/mL) in 100 μL of PBS buffer and incubated overnight at 4 °C. Subsequently, NAD^+^ (100 μM), biotinylated NAD^+^ (25 μM), and slDNA (200 nM) in 30 μL of reaction buffer (2 mM MgCl_2_ and 50 mM Tris, pH 8.0) were added to each well. This was followed by the addition of 5 μL of either the compound at varying concentrations or a solvent control. The reaction was initiated by adding 20 μL of PARP (50 ng/well) and incubating at 30 °C for 1 h. After the reaction, 50 µL of streptavidin-conjugated HRP was introduced, and the assay continued at 30 °C for an additional 30 min. Finally, 100 μL of a solution containing H_2_O_2_ and luminol in 0.1 M citrate buffer (pH 5.4) was added, and the luminescent signal was recorded using a SpectraMax M5 microplate reader (Molecular Devices™, San Jose, CA, USA). The inhibition rate of PARP1 enzymatic activity was determined as (Lu control − Lu treated)/Lu control × 100%. IC_50_ values, representing the concentration required for 50% inhibition of PARP1 enzymatic activity, were determined using non-linear regression with a normalized dose–response fit in GraphPad software (version 8.3.0). Following the described method, the inhibition rates of **DDY01**–**DDY11** against PARP1 were initially determined at a concentration of 0.5 µM. Subsequently, the inhibition rates of some selected compounds at nine concentrations (0.01 nM, 0.03 nM, 0.1 nM, 0.33 nM, 1 nM, 3.3 nM, 10 nM, 33 nM, 100 nM) were individually tested and obtained. IC_50_ values were determined using non-linear regression analysis in Graphpad Prism, employing concentration–response data.

### 3.4. Cell Culture

MDA-MB-468, MDA-MB-231, MCF-7, and MCF-10A cell lines were obtained from the National Collection of Authenticated Cell Culture, China. The cells were cultured in DMEM medium supplemented with 10% FBS and 1% penicillin/streptomycin, and maintained at 37 °C in a humidified atmosphere with 5% CO_2_.

### 3.5. Cell Viability Assay

Cell viability was assessed using the MTT assay. Cells were seeded at a density of 3 × 10^3^ cells per well in a 96-well plate and treated with various concentrations (0.05, 0.2, 0.8, 3.2, 6.4, 12.8 µM) of the compounds for 72 h. After incubation, 100 µL of MTT solution (0.5 mg/mL) was added to each well and further incubated for 4 h. The resulting formazan crystals were dissolved in 150 µL of DMSO, and the absorbance was measured at 570 nm using a microplate reader.

### 3.6. Western Blot Analysis

For Western blotting, cells were treated with **DDY02**, FK866, and Olaparib for 24 h. Cells were then lysed using RIPA buffer supplemented with protease and phosphatase inhibitors. Protein concentrations were determined using the BCA Protein Assay Kit (Thermo Fisher Scientific^TM^, Waltham, MA, USA). Equal amounts of protein (20–30 µg) were separated by SDS-polyacrylamide gel electrophoresis and transferred to a polyvinylidene fluoride (PVDF) membrane. The membrane was blocked with 5% milk in PBS before incubation with primary antibodies. Primary antibodies against γ-H2AX (Cell Signaling Technology™, Danvers, MA, USA), BCL2 (Abcam™, Cambridge, UK), and β-actin (Proteintech™, Rosemont, IL, USA) were used. After overnight incubation at 4 °C, membranes were washed and incubated with HRP-conjugated secondary antibodies for 1 h. Protein bands were visualized using ECL reagents (Labgic Technology^TM^, Beijing, China).

### 3.7. Apoptosis Assays

The Annexin V-FITC/PI cell apoptosis detection kit (KeyGen Biotech, Nanjing, China) was employed for the analysis of cell apoptosis. MDA-MB-468 cells (5 × 10^5^ cells/mL) were seeded in six-well plates and treated with compounds at corresponding concentrations for 72 h. Subsequently, MDA-MB-468 cells were harvested and incubated with 5 μL of Annexin V-FITC and 5 μL of propidium iodide (PI) in the dark for 15 min at room temperature. Apoptosis data were assessed using flow cytometry (Accuri C6 Plus, BD Bioscience, San Jose, CA, USA).

### 3.8. Clonogenic Assay

MDA-MB-468 cells were plated in 6-well plates at a density of 500 cells/well and allowed to grow for 7 days to promote colony formation. Following the incubation period, cells were washed with PBS, fixed in methanol for 15 min, and stained with 1% crystal violet for 1 h. Subsequently, colonies were imaged and counted.

### 3.9. Cell Migration Assay

MDA-MB-468 cells were plated in 6-well plates at a density of 5 × 10^5^ cells/well and allowed to incubate overnight. A scratch was carefully generated in the cell monolayer using a 10 µL pipette tip. Following the scratch, cells were washed with PBS and treated with **DDY02**, FK866, or Olaparib. Images were acquired at 0, 12, 24, and 48 h time points to monitor and analyze the migration of cells over the specified intervals.

### 3.10. Comet Assay

The comet assay was conducted under alkaline conditions using the Comet Assay Kit (Beyotime, Shanghai, China) following the manufacturer’s guidelines. Briefly, after treating cells with **DDY02**, FK866, Olaparib, and their combinations for 24 h, cells were harvested and combined with 0.7% low-melting-point agarose at a 1:7 (*v*/*v*) ratio. This mixture was spread onto microscope slides and immersed in lysis solution for 1 h. Electrophoresis was carried out in a horizontal apparatus at 25 V and 300 mA. After electrophoresis, slides were stained with propidium iodide (PI) for DNA visualization, and images were captured using a fluorescence inverted microscope.

### 3.11. NAD Level Determination Assay

According to the manufacturer’s instructions, an NAD^+^/NADH Assay kit with WST-8 (Beyotime, China) was used to measure the intracellular NAD level. The cells collected by centrifugation were added to NAD^+^/NADH lysis buffer to obtain a cell lysate. After adding acetaldehyde dehydrogenase working solution, the lysate was incubated at 37 °C for 10 min, and then followed by the addition of color-developing solution. The total concentration of NADH and NAD can be measured by detecting the absorbance at 450 nm. To measure the concentration of NADH, the cell lysate was incubated at 60 °C for 30 min before adding acetaldehyde dehydrogenase working solution. Finally, the NAD content is obtained by the subtraction [NAD^+^] = [NADH + NAD^+^] − [NADH].

## 4. Conclusions

Breast cancer is a malignant tumor that seriously threatens the health of women all over the world. In recent years, the incidence of breast cancer in China has increased year by year and showed a trend of younger age. Although the proportion of TNBC in breast cancer patients is only 15~20%, it is the most malignant subtype, prone to postoperative recurrence and metastasis [41]. Because ER, PR, and HER-2 are negative, TNBC lacks effective drug targets. Moreover, the duration of remission of TNBC with traditional chemotherapy drugs is shorter than other breast cancers. Thus, the development of novel anti-TNBC therapies, particularly those involving innovative mechanisms of action, has become a critical focus in both academic research and pharmaceutical development. In recent years, various dual-target inhibitors acting on tumor-related targets have been reported and drawn significant interest.

NAMPT and PARP1 are two key enzymes upstream and downstream of NAD metabolism. The combination of NAMPT inhibitors and PAPR1 inhibitors showed a synergistic effect in the treatment of TNBC. In this work, we designed and synthesized eleven NAMPT/PARP1 dual-target inhibitors based on the pharmacophore fusion strategy. Among these, **DDY02** emerged as the most effective, exhibiting significant proliferation inhibition in MDA-MB-468 cells. Our results showed that **DDY02** could reduce intracellular NAD in a dose-dependent manner, with NMN able to mitigate this anti-proliferative effect, suggesting that **DDY02**’s efficacy was directly linked to NAMPT inhibition. Western blot assay and comet assay indicated that **DDY02** induced DNA damage, likely due to its inhibition of PARP1. In addition, **DDY02** could induce apoptosis, arrest cell cycle progression, and inhibit cell migration.

It should be pointed out that there are still some deficiencies in this work that need improvement. The molecular weights of these dual-target inhibitors exceed 600, which challenges the drug-like properties by violating Lipinski’s “Rule of Five”. Additionally, the pyridine structure in these molecules is susceptible to rapid metabolism by cytochrome P450 enzymes, which could lead to unfavorable pharmacokinetic properties. The heterogeneity of TNBC also means that the effectiveness of these inhibitors may vary across different subtypes, potentially limiting their applicability. Furthermore, while the cytotoxic activities of our synthetic compounds are comparable to reference drugs, their potential synergistic effects may be diminished by factors such as low solubility, limited cellular uptake, or rapid degradation within the cellular environment. Finally, the limited selectivity of **DDY02** between cancer and normal cells underscores the importance of further investigating the safety profile of such dual-target inhibitors.

Despite existing patents for NAMPT/PARP1 dual-target inhibitors, the data disclosed in these documents are insufficient for a comprehensive evaluation of their potential. Our research acts as a pivotal conceptual validation, providing detailed insights into the drug design, structure–activity relationship, and mechanisms of action of these inhibitors. This study will serve as a valuable resource for researchers in the field. In conclusion, our study identified a promising lead compound, **DDY02**, for the treatment of TNBC. Further structural optimization and animal in vivo experiments will be conducted to validate its efficacy and safety profile.

## Figures and Tables

**Table 1:**
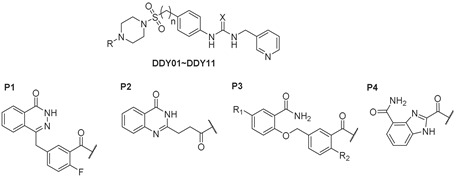
The inhibitory activities of target compounds against NAMPT and PARP1.

Compound	R	X	*n*	R_1_	R_2_	NAMPT IC_50_ (μM) ^a^ or Inhibition Rate (0.1 μM, %) ^b^	PARP1 IC_50_ (μM) ^a^ or Inhibition Rate (0.5 μM, %) ^b^
FK866	—	—	—	—	—	0.012 ± 0.004	—
Olaparib	—	—	—	—	—	—	0.002 ± 0.001
**DDY01**	P1	S	0	—	—	83%	0.009 ± 0.002
**DDY02**	P1	O	0	—	—	0.010 ± 0.003	0.050 ± 0.010
**DDY03**	P2	O	0	—	—	0.055 ± 0.019	0.025 ± 0.007
**DDY04**	P3	S	0	H	F	84%	70%
**DDY05**	P3	O	0	H	F	86%	1.879 ± 0.600
**DDY06**	P3	O	1	H	F	48%	89%
**DDY07**	P3	O	0	F	F	84%	92%
**DDY08**	P3	O	0	H	H	80%	69%
**DDY09**	P3	O	0	F	H	86%	78%
**DDY10**	P4	S	0	—	—	7%	55%
**DDY11**	P4	O	0	—	—	0.034 ± 0.009	0.014 ± 0.003

^a^ IC_50_ values are the mean of at least two independent experiments performed in duplicate with standard deviation. ^b^ Inhibition rates were tested in duplicate; the average values were obtained.

**Table 2:**
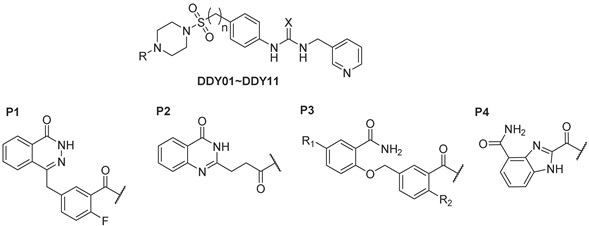
In vitro anti-proliferative activity of target compounds against breast cancer cell lines.

Compound	R	X	*n*	R1	R2	Cytotoxicity IC_50_ (μM) *		
						MDA-MB-231	MDA-MB-468	MCF-7
FK866	—	—	—	—	—	3.83 ± 0.12	4.01 ± 0.21	5.8 ± 0.86
Olaparib	—	—	—	—	—	6.74 ± 0.08	6.19 ± 0.09	8.68 ± 0.81
FK866 + Olaparib (1:1)	—	—	—	—	—	3.14 ± 0.09	3.59 ± 0.10	5.31 ± 0.03
**DDY01**	P1	S	0	—	—	6.17 ± 0.27	5.09 ± 0.09	8.48 ± 0.18
**DDY02**	P1	O	0	—	—	10.43 ± 0.16	4.43 ± 0.04	7.61 ± 0.14
**DDY03**	P2	O	0	—	—	10.88 ± 0.68	8.17 ± 0.31	11.23 ± 0.79
**DDY04**	P3	S	0	H	F	13.48 ± 0.66	14.06 ± 0.3	13.37 ± 2.41
**DDY05**	P3	O	0	H	F	10.22 ± 0.29	8.02 ± 0.74	>20
**DDY06**	P3	O	1	H	F	15.36 ± 2.15	17.16 ± 1.57	7.95 ± 0.34
**DDY07**	P3	O	0	F	F	8.1 ± 0.71	7.43 ± 0.23	13.47 ± 0.16
**DDY08**	P3	O	0	H	H	11.09 ± 0.19	12.89 ± 1.69	9.18 ± 0.44
**DDY09**	P3	O	0	F	H	8.7 ± 0.24	6.98 ± 0.57	11.01 ± 0.82
**DDY10**	P4	S	0	—	—	4.45 ± 0.29	6.74 ± 0.16	8.83 ± 0.31
**DDY11**	P4	O	0	—	—	13.83 ± 0.33	12.22 ± 1.39	7.39 ± 0.34

* Data are expressed as the mean ± SEM for at least 3 independent experiments.

**Table 3:** In vitro anti-proliferative activity of target compounds against MCF-10A cell lines.

Compound	Cell Survival Rate (3.0 μM, %) *	Cytotoxicity IC_50_ *
FK866	65.49 ± 0.41	4.41 ± 0.22
Olaparib	91.76 ± 1.78	9.61 ± 0.77
Olaparib + FK866	65.31 ± 0.03	—
**DDY01**	67.1 ± 1.73	—
**DDY02**	70.28 ± 1.06	5.31 ± 0.12
**DDY03**	80.71 ± 1.5	—
**DDY04**	98.38 ± 1.74	—
**DDY05**	88.65 ± 2.41	—
**DDY06**	69.46 ± 2.56	—
**DDY07**	70.73 ± 0.07	—
**DDY08**	72.63 ± 3.46	—
**DDY09**	66.93 ± 2.45	—
**DDY10**	96.87 ± 1.71	—
**DDY11**	82.15 ± 0.21	—

* Data are expressed as the mean ± SEM for at least 3 independent experiments.

## Data Availability

Data are contained within the article and Supplementary Materials.

## Supplementary Materials

The following supporting information can be downloaded at https://www.mdpi.com/article/10.3390/molecules29122836/s1: IC_50_ curves of MTT assay, cell cycle analysis, copies of NMR spectra and IR spectra, HPLC data for compounds.
