# Supplementary material for: Targeting NAD Metabolism: Rational Design, Synthesis and In Vitro Evaluation of NAMPT/PARP1 Dual-Target Inhibitors as Anti-Breast Cancer Agents
Source: Molecules. 2024 Jun 14;29(12):2836. doi: 10.3390/molecules29122836 (PMC11206297; doi:10.3390/molecules29122836)
Supplement: Supplementary file 1 [file molecules-29-02836-s001.zip › molecules-3007770-supplementary.pdf]

Supplementary Material

Targeting NAD Metabolism: Rational Design, Synthesis and In Vitro Evaluation of NAMPT/PARP1 Dual-Target Inhibitors as Anti-Breast Cancer Agents

Yingpeng Li [1,2,†], Xianxiu Kong [1,2,†], Xinhong Chu [1,2,†], Hui Fu [3], Xinchi Feng [1,2], Chengcheng Zhao [4], Yanru Deng [1,2] and Jun Ge [1,2,*]

[1] College of Chinese Materia Medica, Tianjin University of Traditional Chinese Medicine, Tianjin 301617, China
[2] Tianjin Key Laboratory of Therapeutic Substance of Traditional Chinese Medicine, Tianjin 301617, China
[3] College of Integrative Medicine, Tianjin University of Traditional Chinese Medicine, Tianjin 301617, China
[4] Experimental Teaching and Practical Training Center, Heilongjiang University of Chinese Medicine, Harbin 150040, China
* Correspondence: gejun@tjutcm.edu.cn
† These authors contributed equally to this work.

Table of Contents

1 IC_{50} curves of MTT assay 2
2 Cell cycle analysis 4
3 NMR spectral data 5
4 IR spectrum data 21
5 HPLC data 27

1 IC_{50} curves of MTT assay

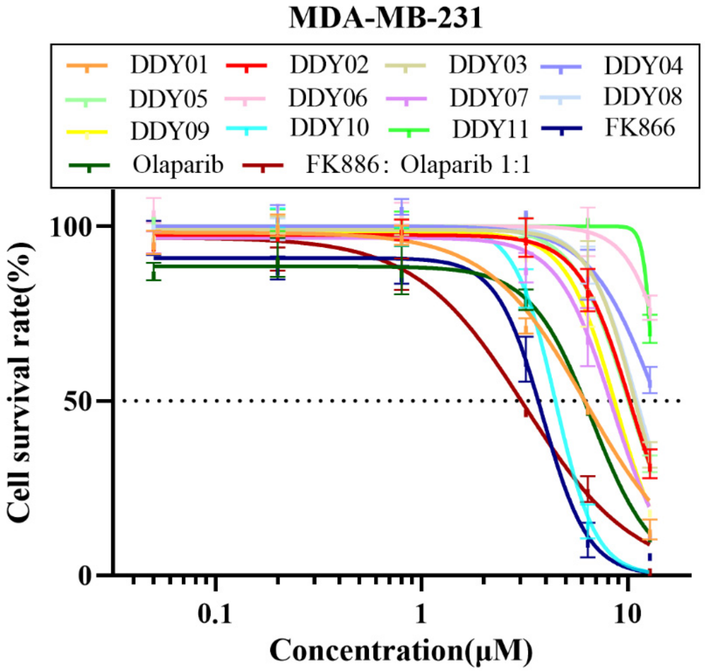

Figure S1. Dose-response curves of MDA-MB-231 cells treated with FK866, Olaparib, FK866 + Olaparib, DDY01~DDY11.

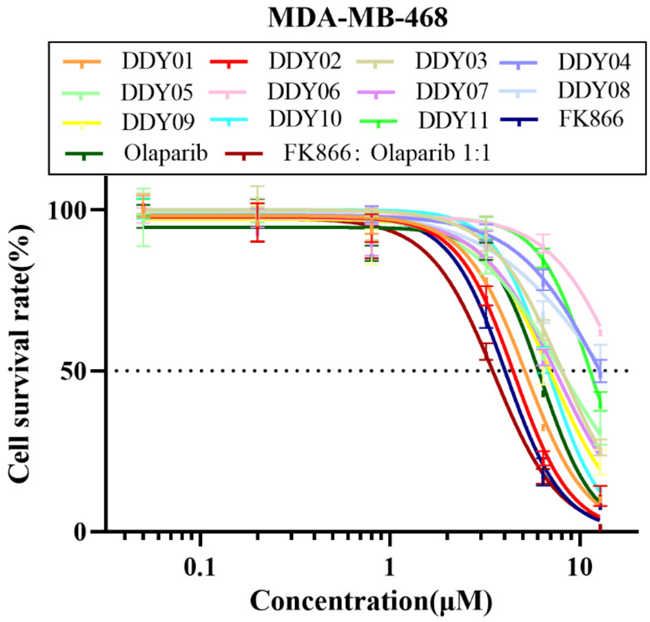

Figure S2. Dose-response curves of MDA-MB-468 cells treated with FK866, Olaparib, FK866 + Olaparib, DDY01~DDY11.

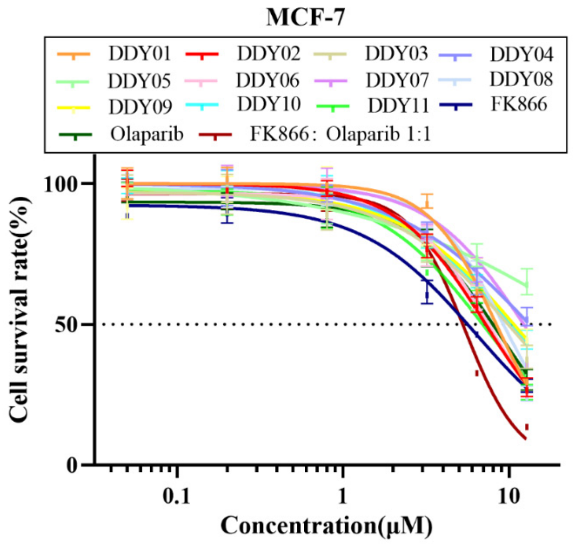

Figure S3. Dose-response curves of MCF-7 cells treated with FK866, Olaparib, FK866 + Olaparib, DDY01~DDY11.

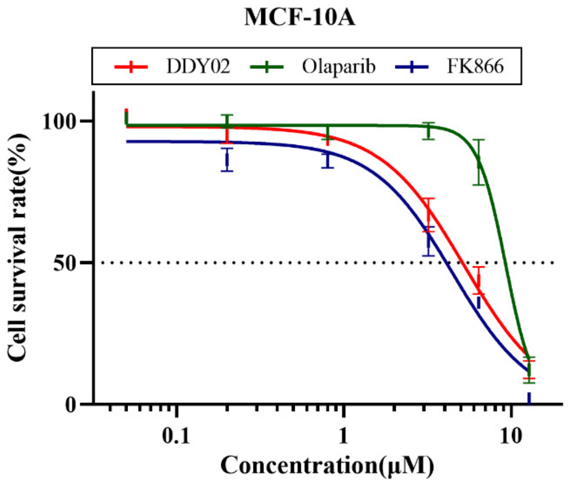

Figure S4. Dose-response curves of MCF-10A cells treated with FK866, Olaparib, DDY02.

2 Cell cycle analysis

Cell cycle distribution was determined using a DNA Content Quantitation Assay Kit (Solarbio, China). MDA-MB-468 cells were seeded in six-well plates and treated with various concentrations of DDY02, Olaparib, FK866, and their combinations for 48 hours. After treatment, cells were harvested, fixed in 70% ethanol at 4°C overnight, and washed with PBS. Cells were then incubated with RNase (100 μL) at 37°C for 30 minutes followed by propidium iodide (PI) staining (400 μL, 30 minutes, 4°C in the dark). Cell cycle distribution was analyzed using an Accuri C6 Plus flow cytometer (BD Biosciences, CA) and data were processed using FlowJo software.

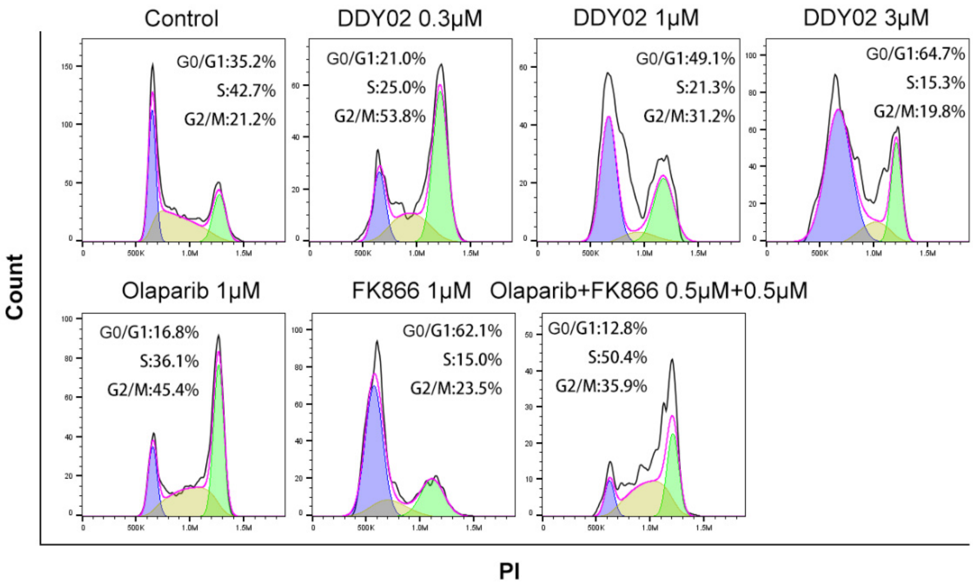

Figure S5. DDY02 induced cell cycle of MDA-MB-468 cells arrest in the G1 phase.

3 NMR spectral data

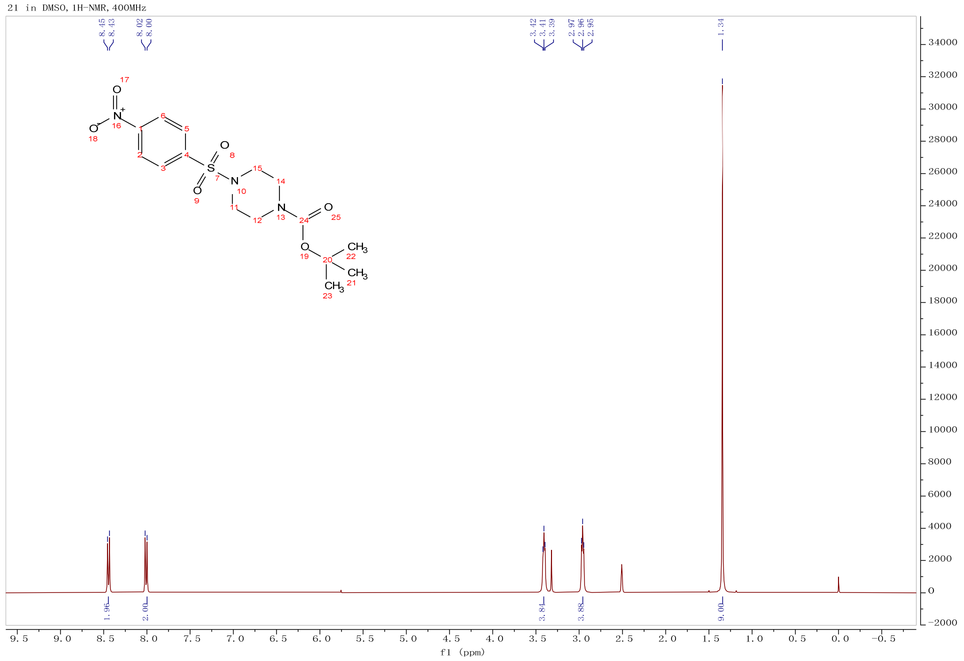

^{1}H-NMR spectrum of compound 21

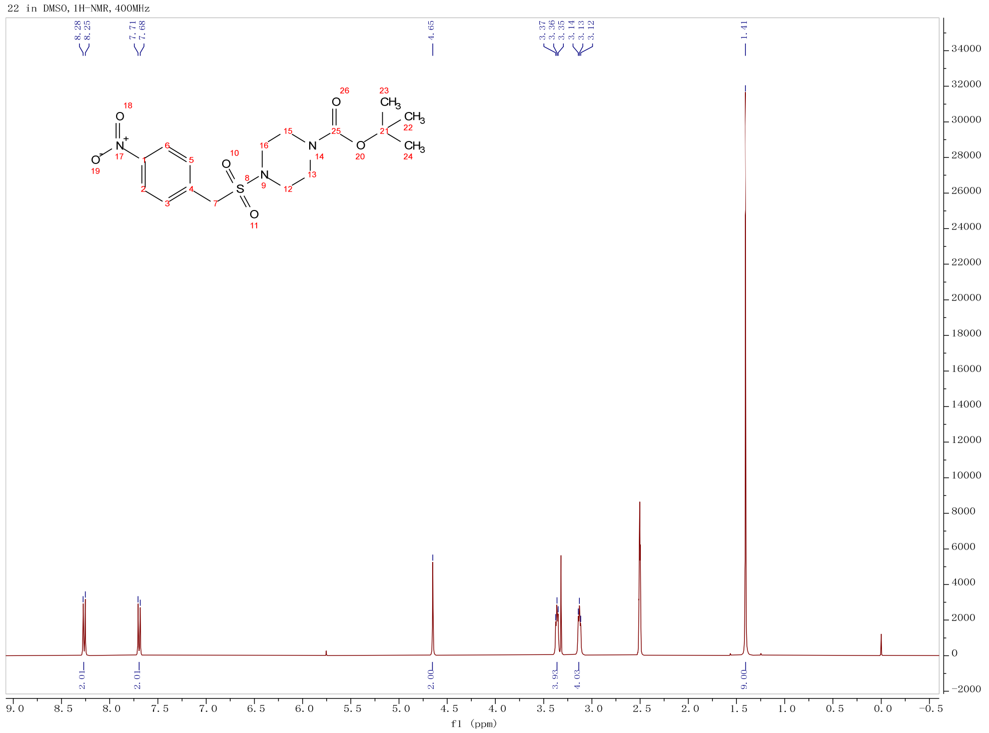

^{1}H-NMR spectrum of compound 22

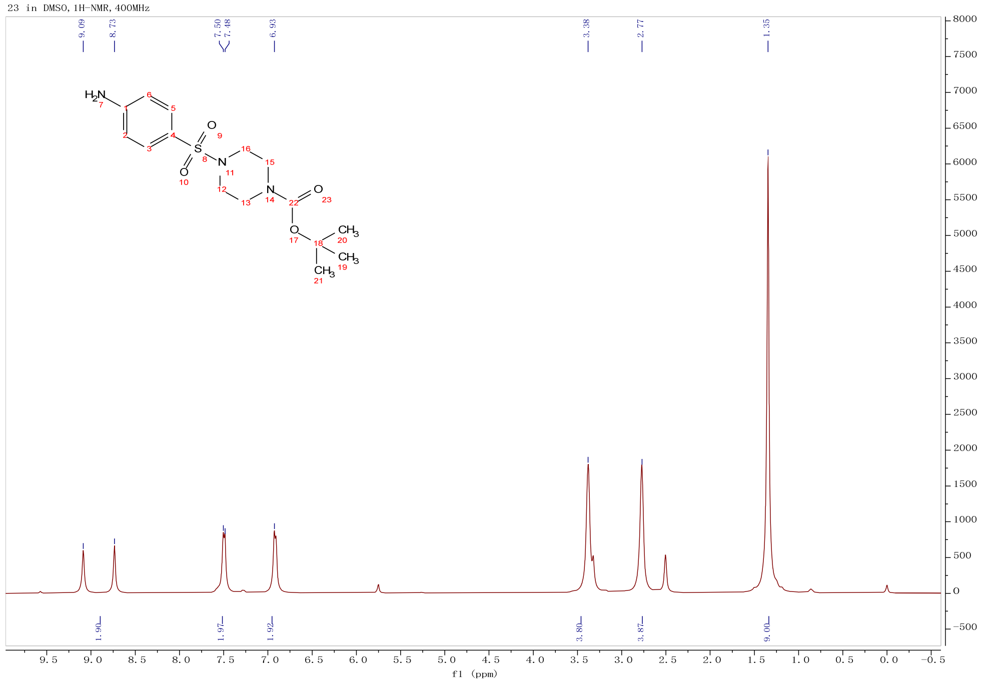

^{1}H-NMR spectrum of compound 23

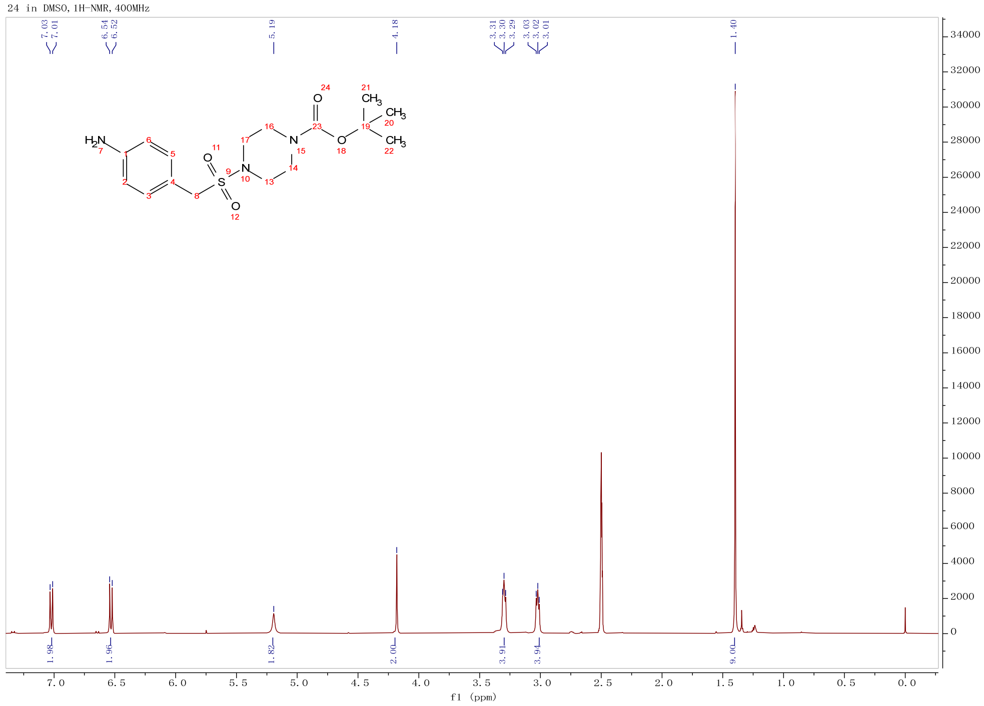

^{1}H-NMR spectrum of compound 24

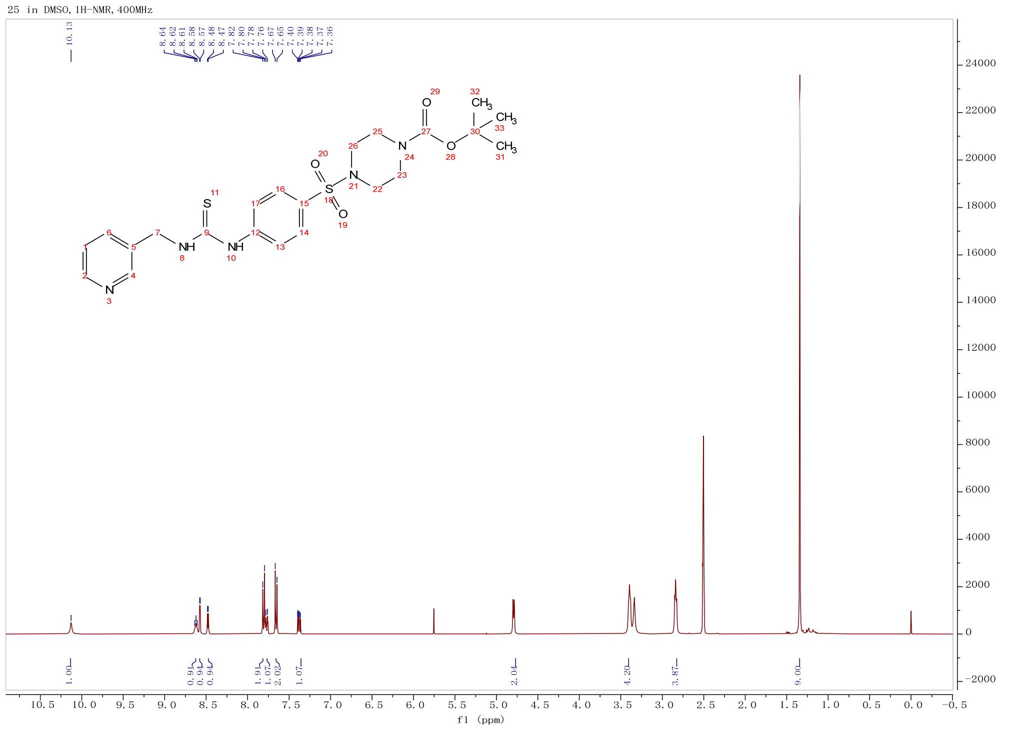

^{1}H-NMR spectrum of compound 25

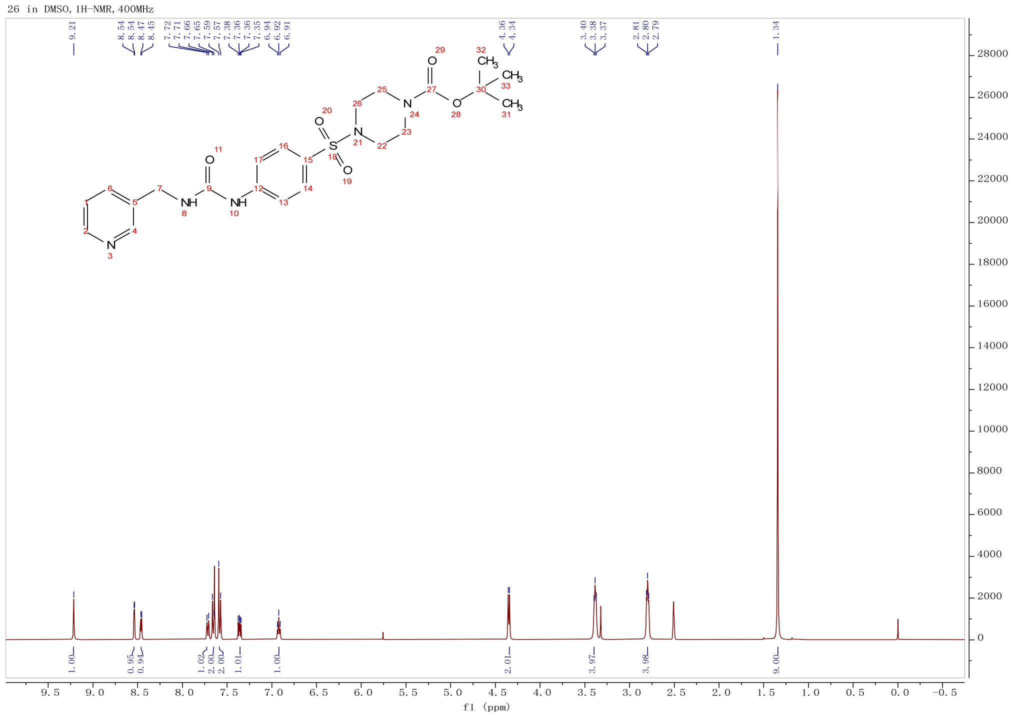

^{1}H-NMR spectrum of compound 26

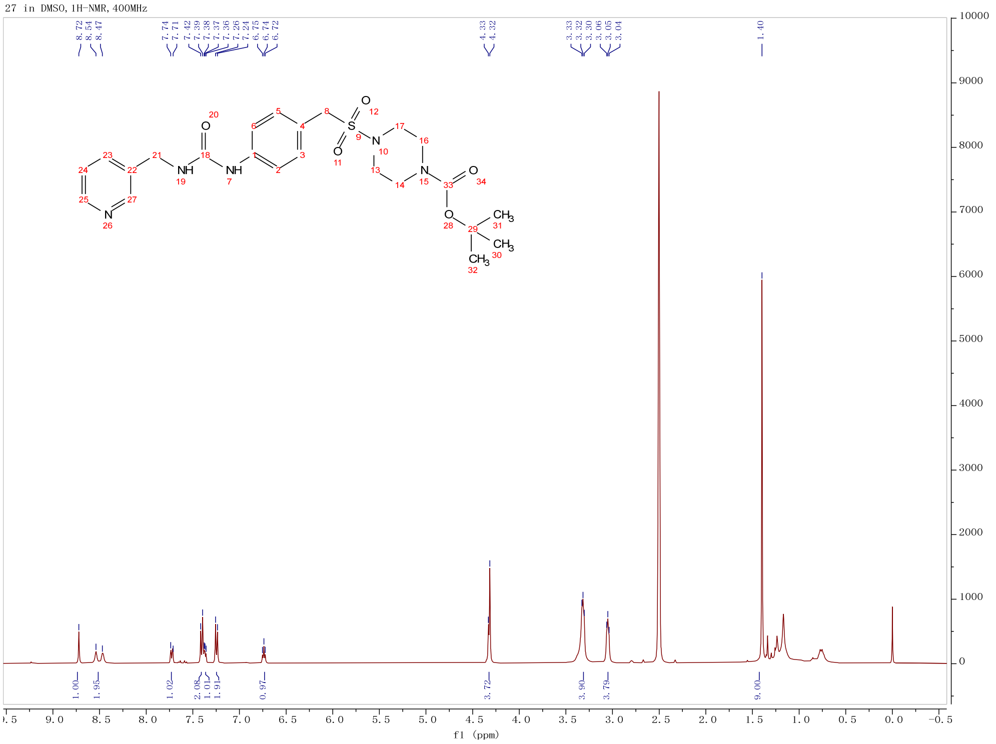

^{1}H-NMR spectrum of compound 27

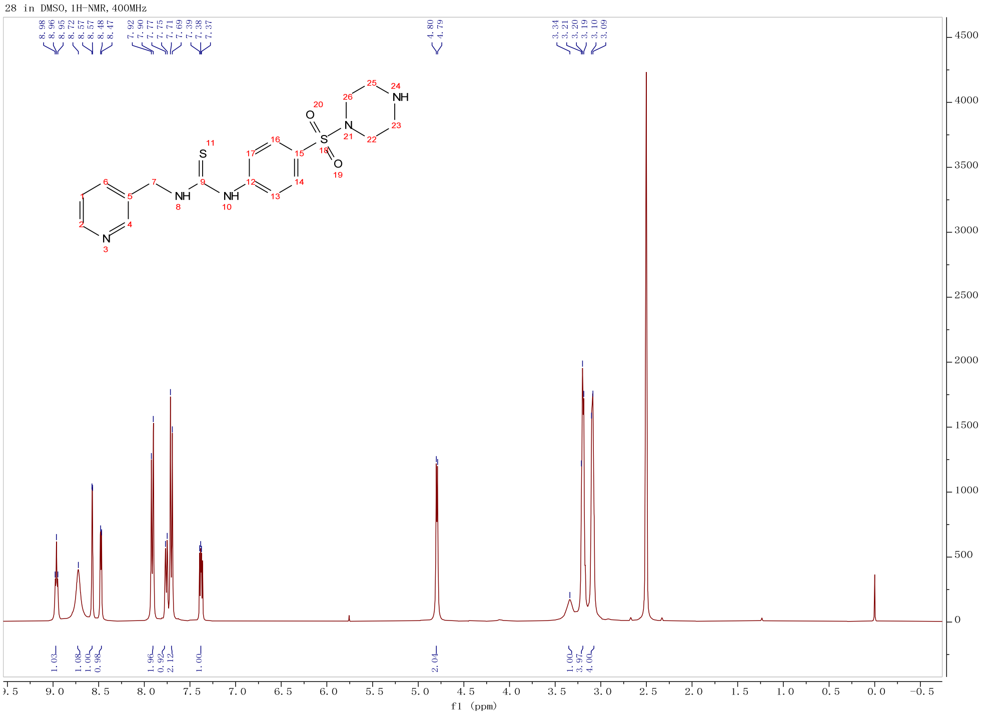

^{1}H-NMR spectrum of compound 28

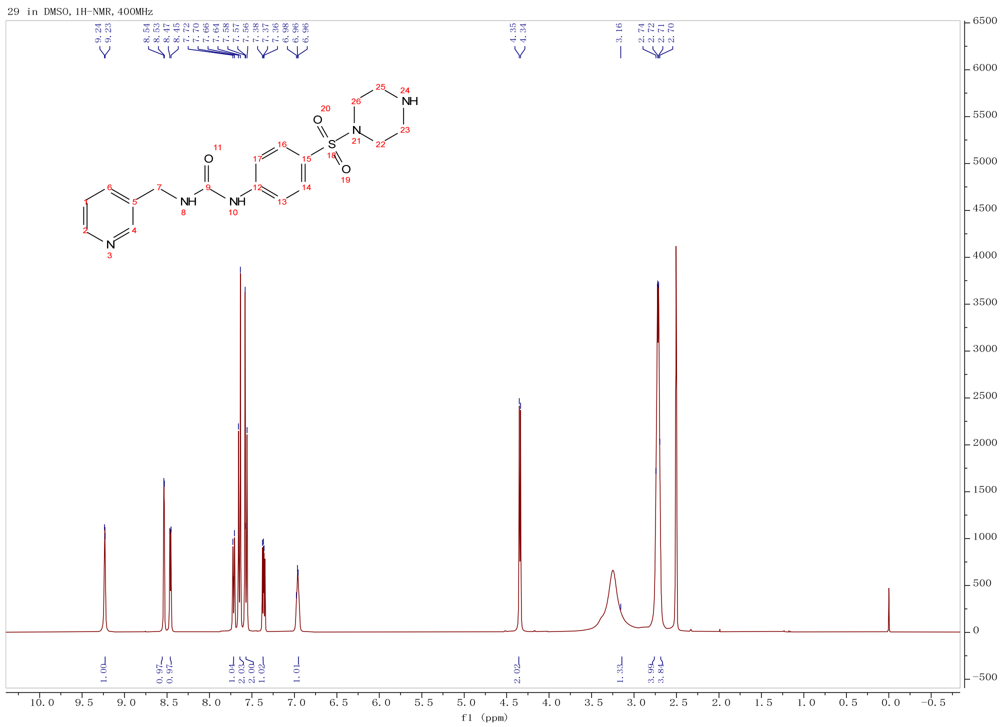

^{1}H-NMR spectrum of compound 29

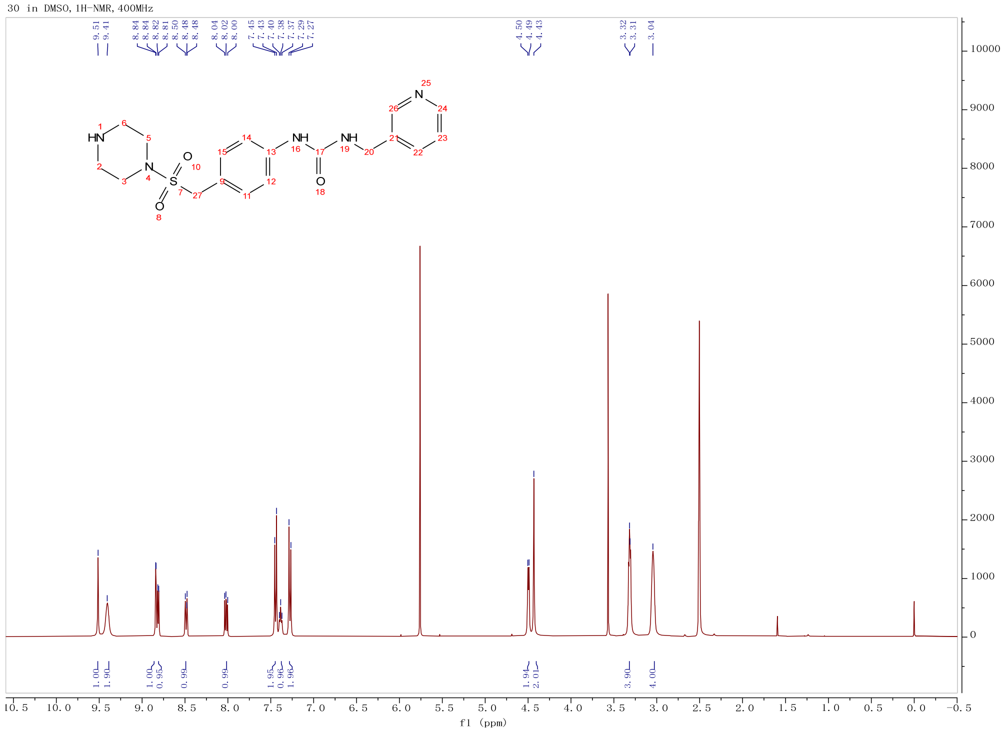

^{1}H-NMR spectrum of compound 30

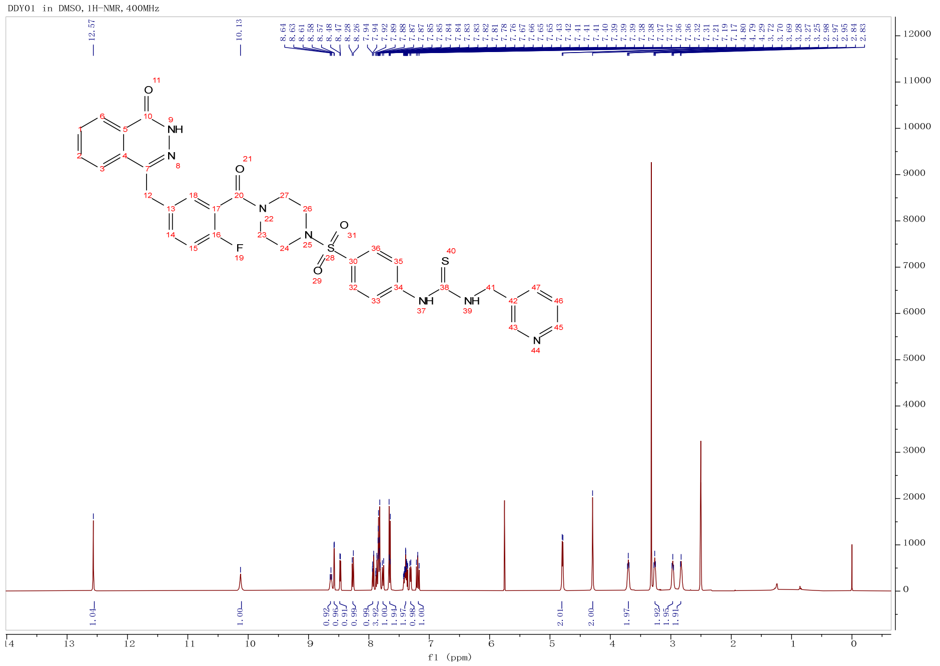

^{1}H-NMR spectrum of compound DDY01

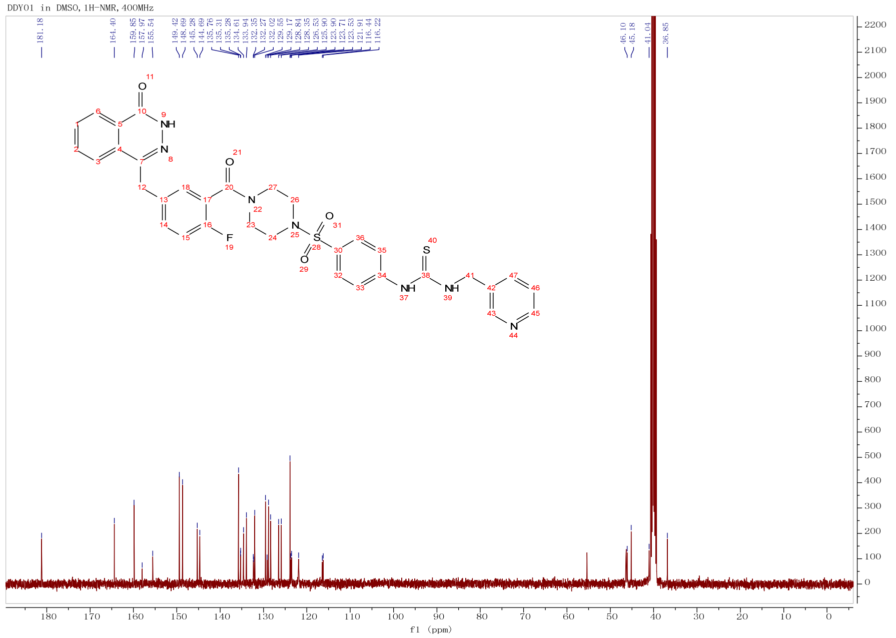

^{13}C-NMR spectrum of compound DDY01

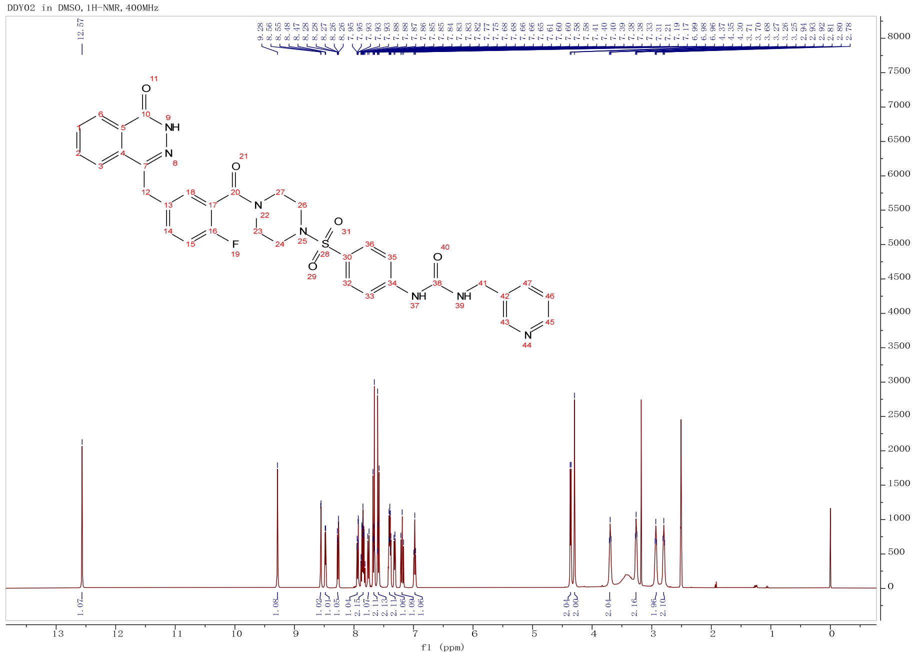

^{1}H-NMR spectrum of compound DDY02

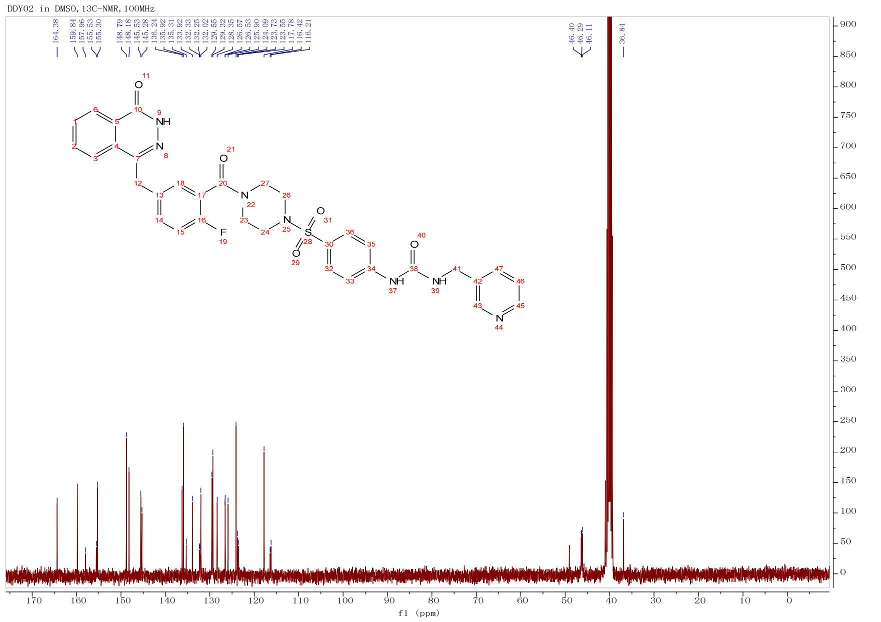

^{13}C-NMR spectrum of compound DDY02

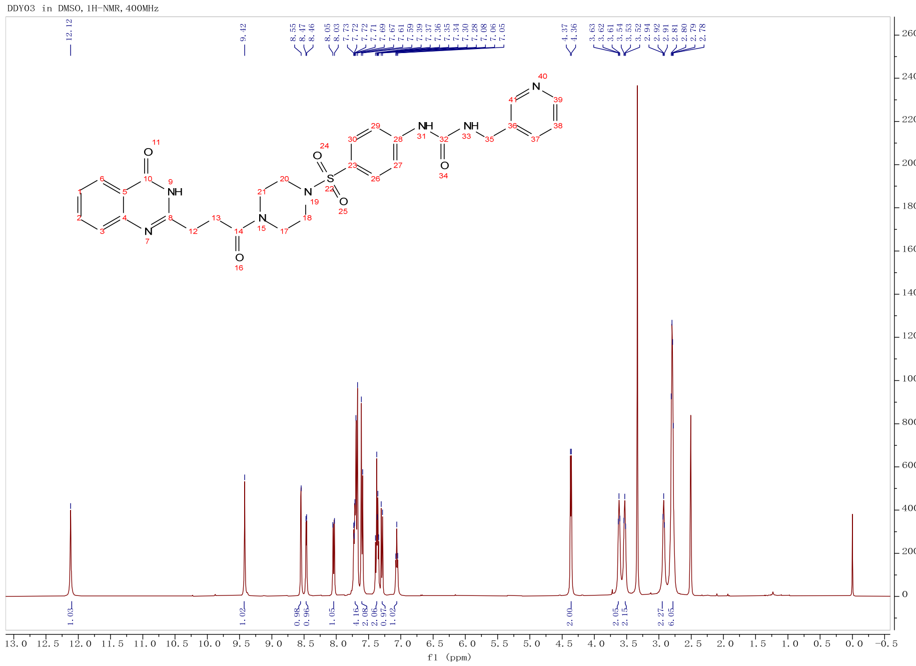

^1H-NMR spectrum of compound DDY03

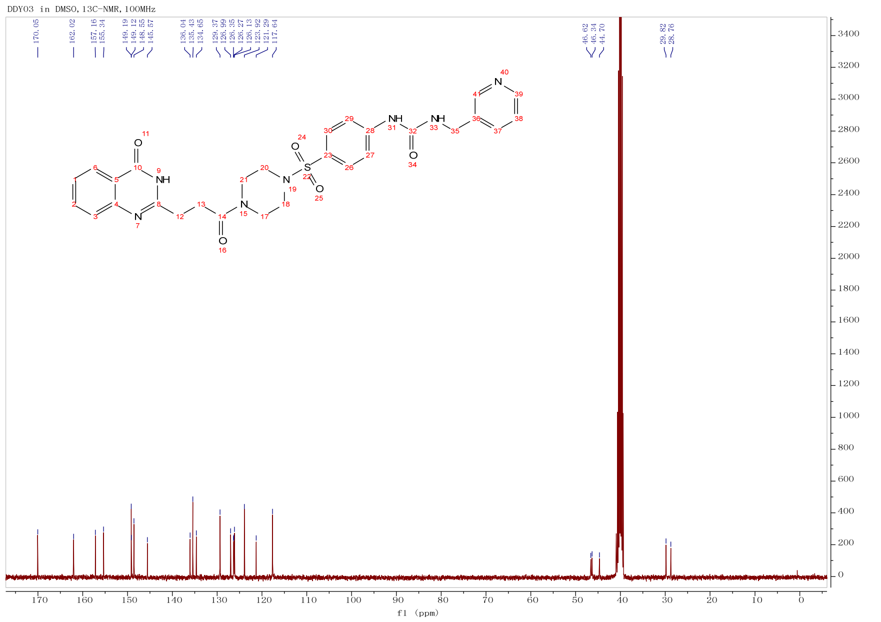

^{13}C-NMR spectrum of compound DDY03

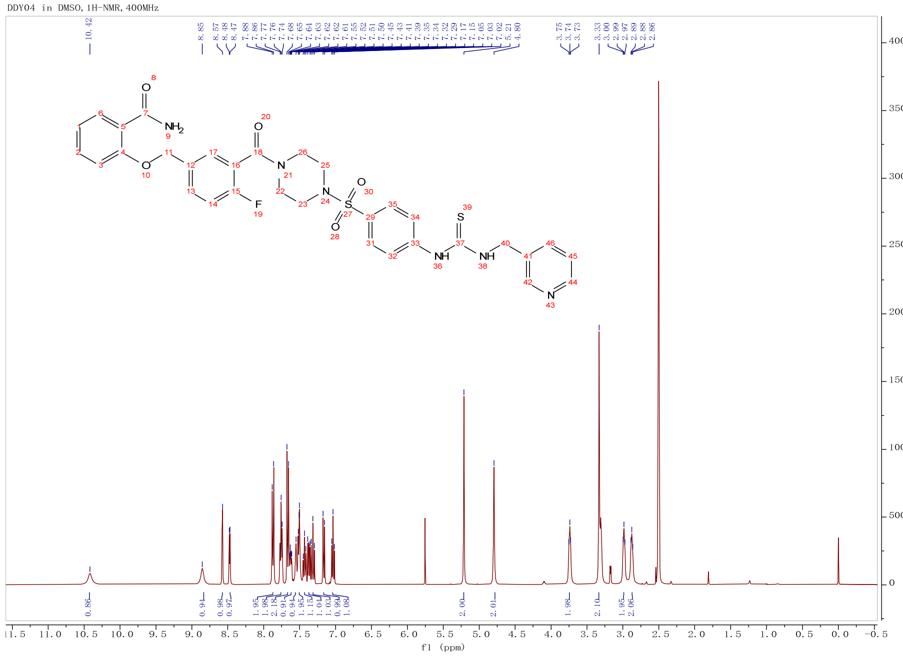

^{1}H-NMR spectrum of compound DDY04

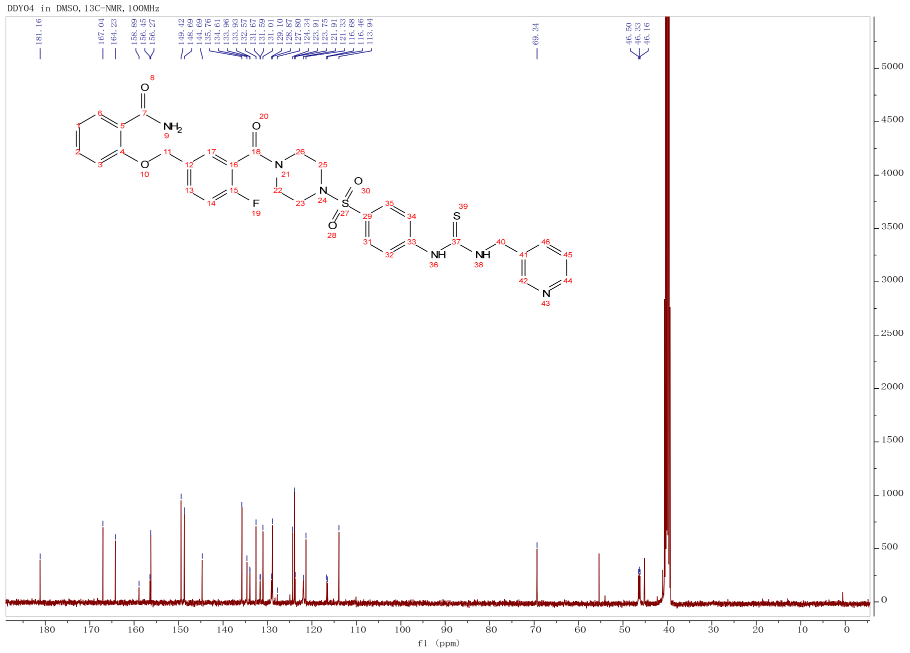

^{13}C-NMR spectrum of compound DDY04

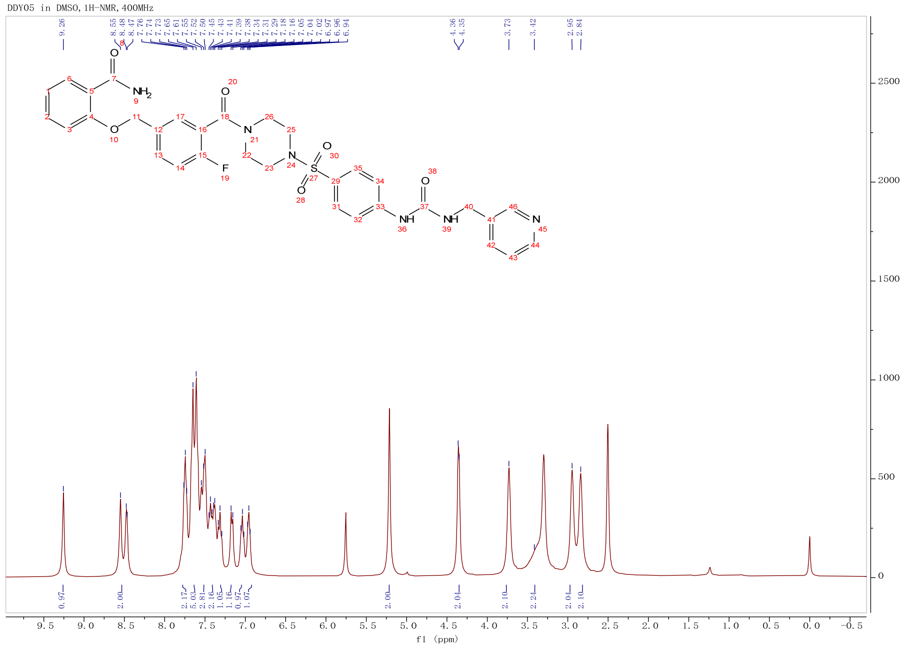

^{1}H-NMR spectrum of compound DDY05

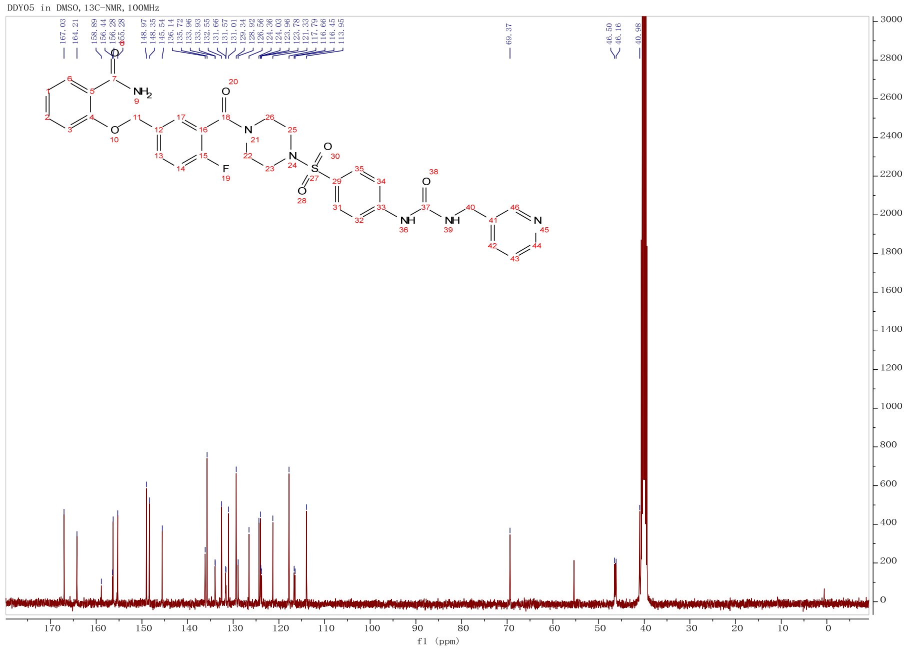

^{13}C-NMR spectrum of compound DDY05

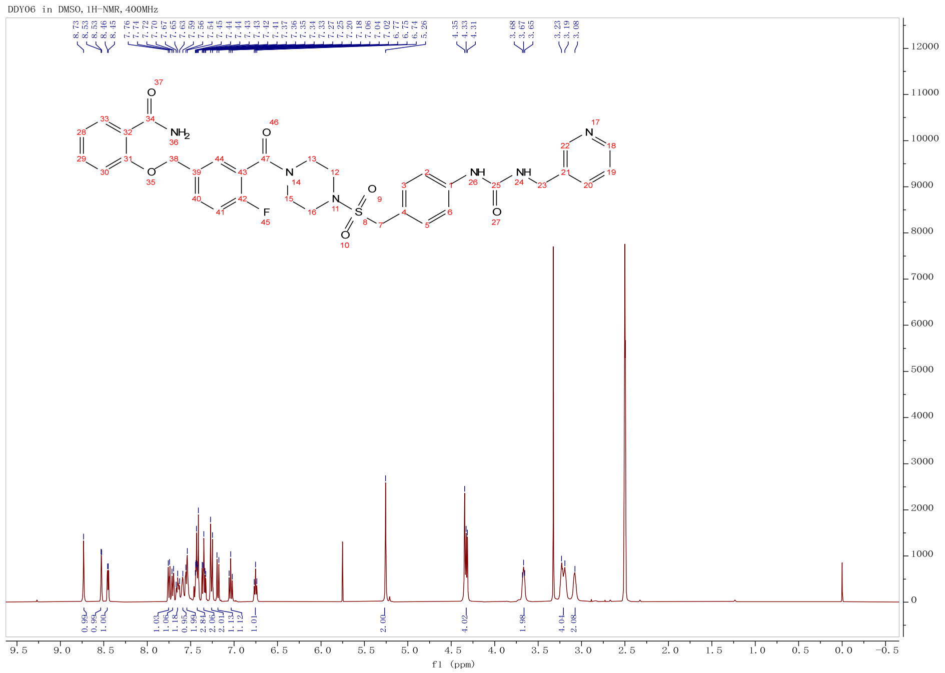

^{1}H-NMR spectrum of compound DDY06

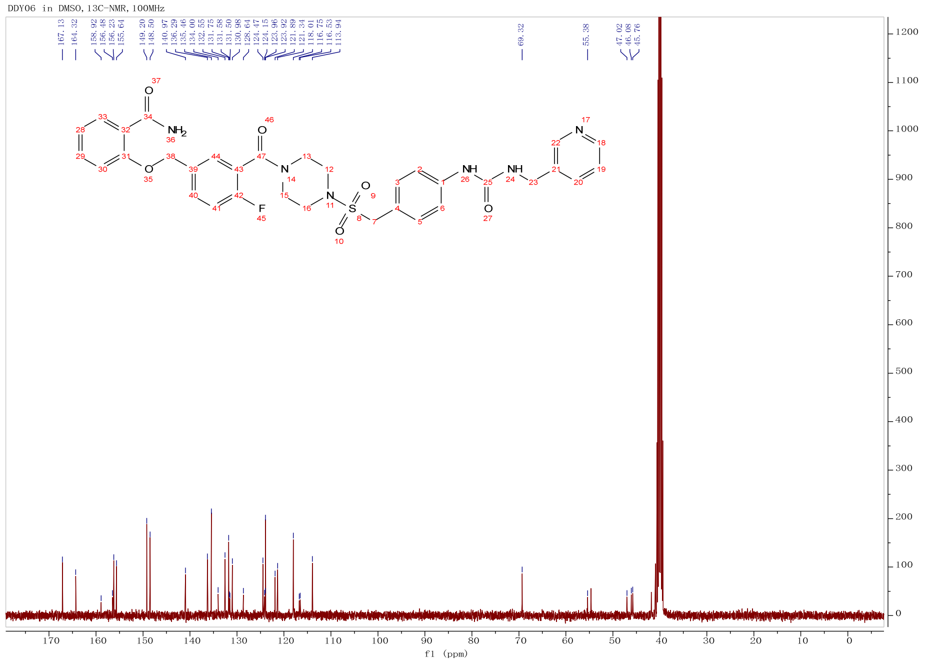

^{13}C-NMR spectrum of compound DDY06

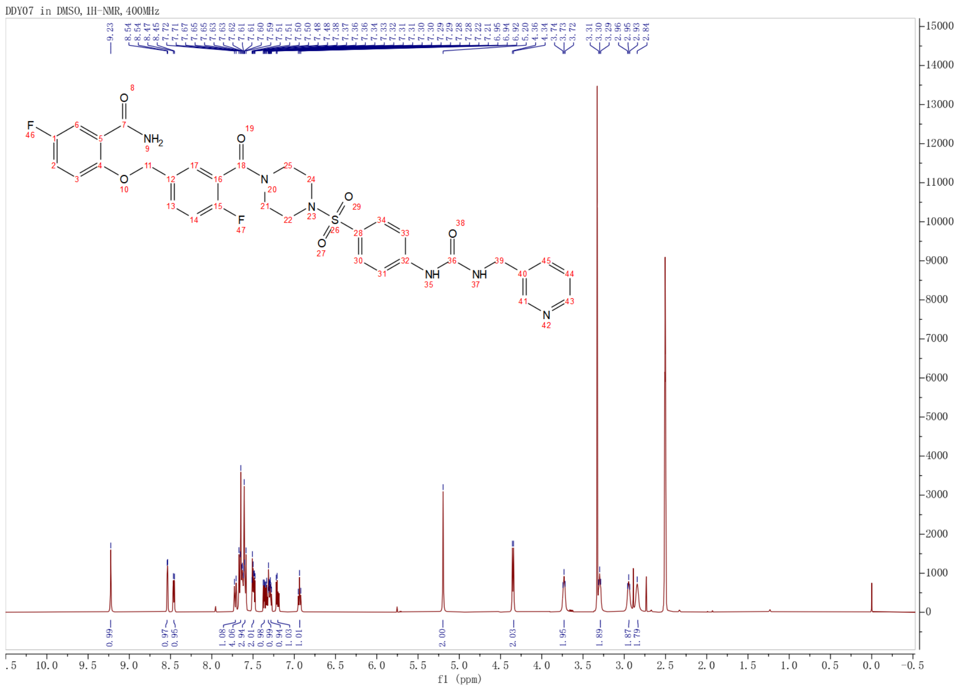

^{1}H-NMR spectrum of compound DDY07

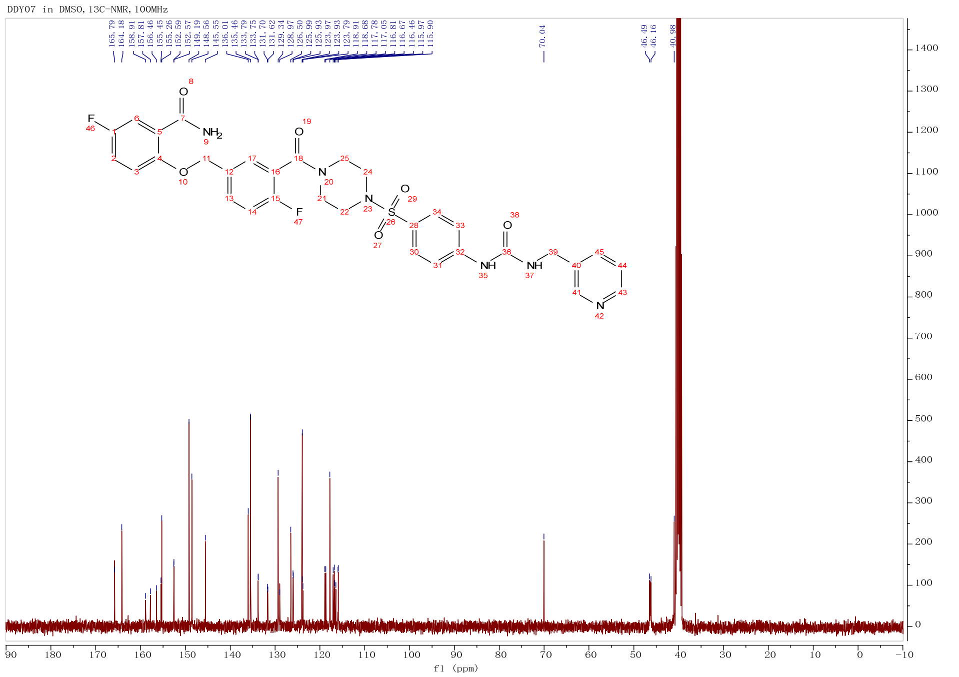

^{13}C-NMR spectrum of compound DDY07

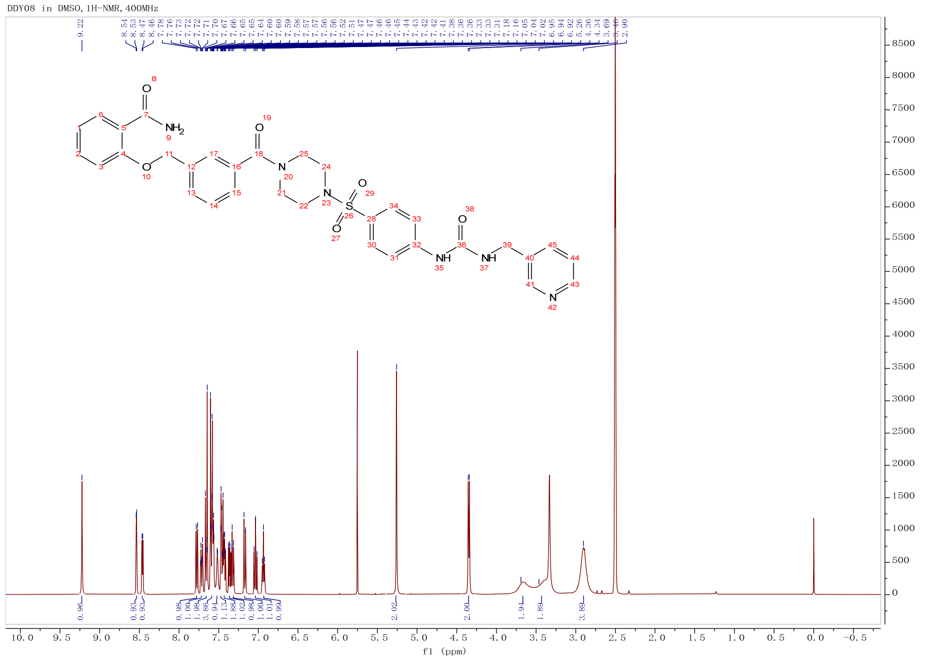

^{1}H-NMR spectrum of compound DDY08

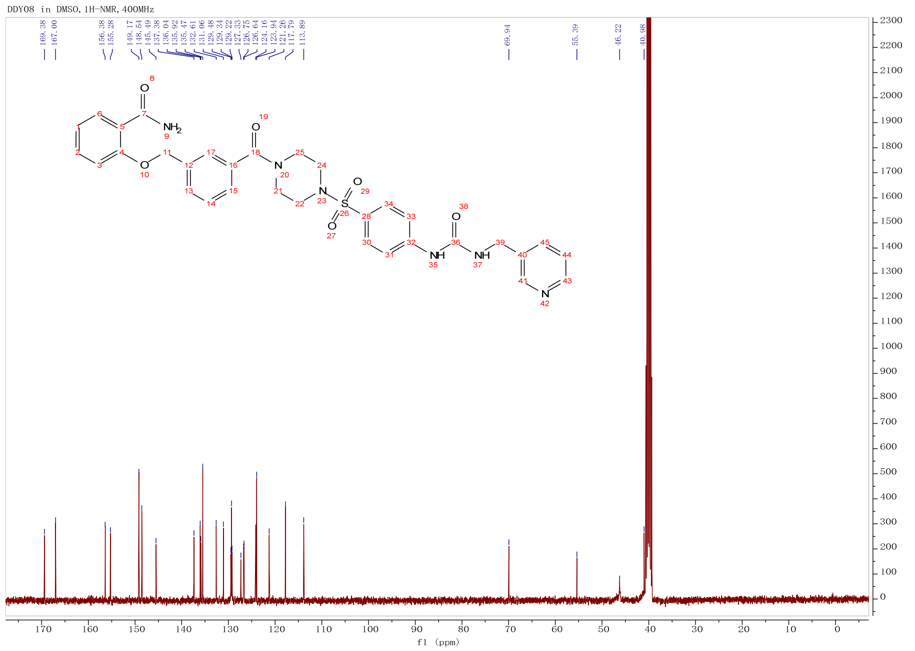

^{13}C-NMR spectrum of compound DDY08

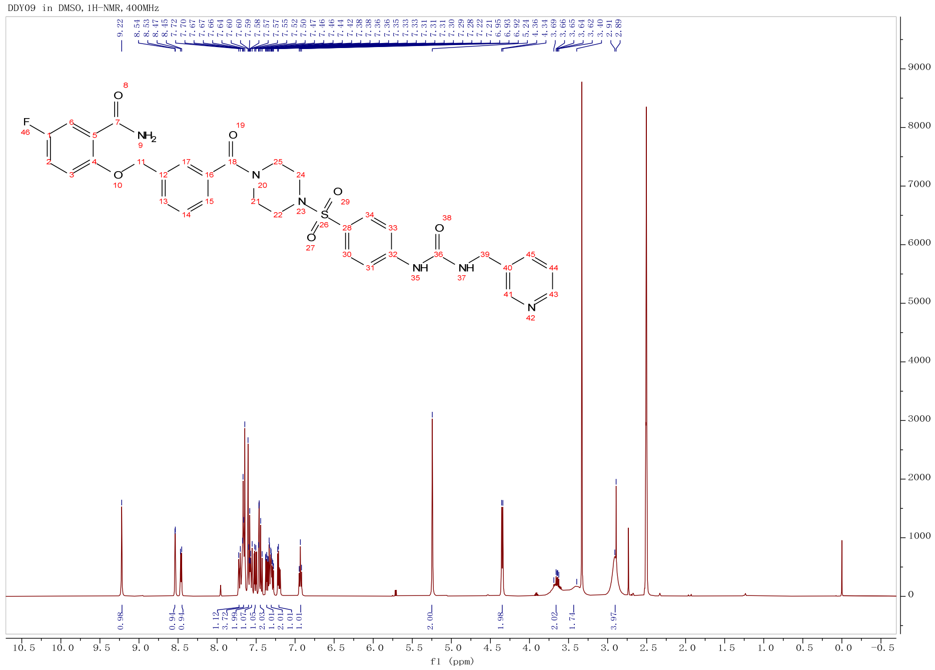

¹H-NMR spectrum of compound DDY09

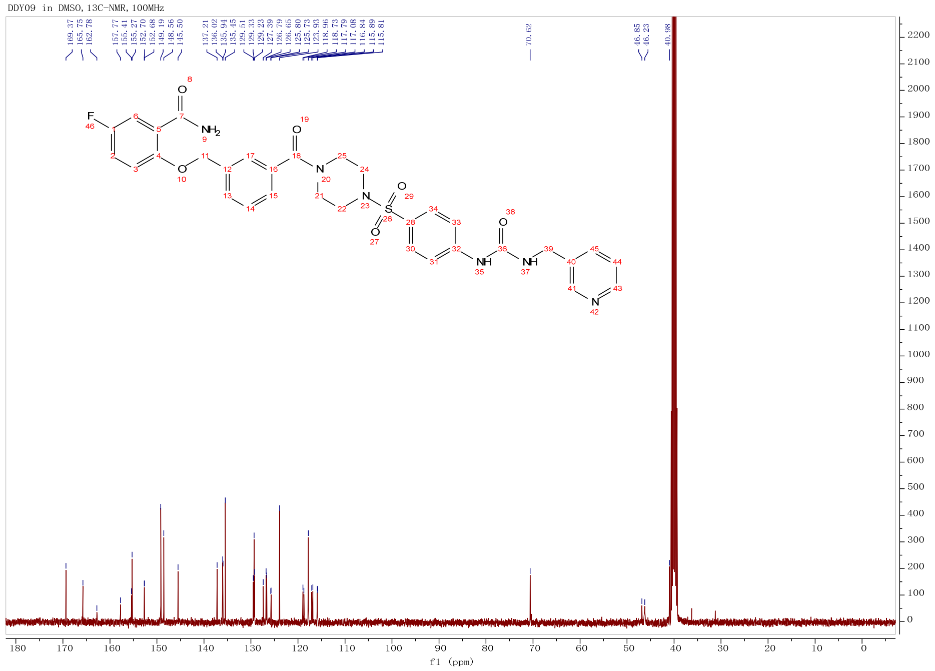

¹³C-NMR spectrum of compound DDY09

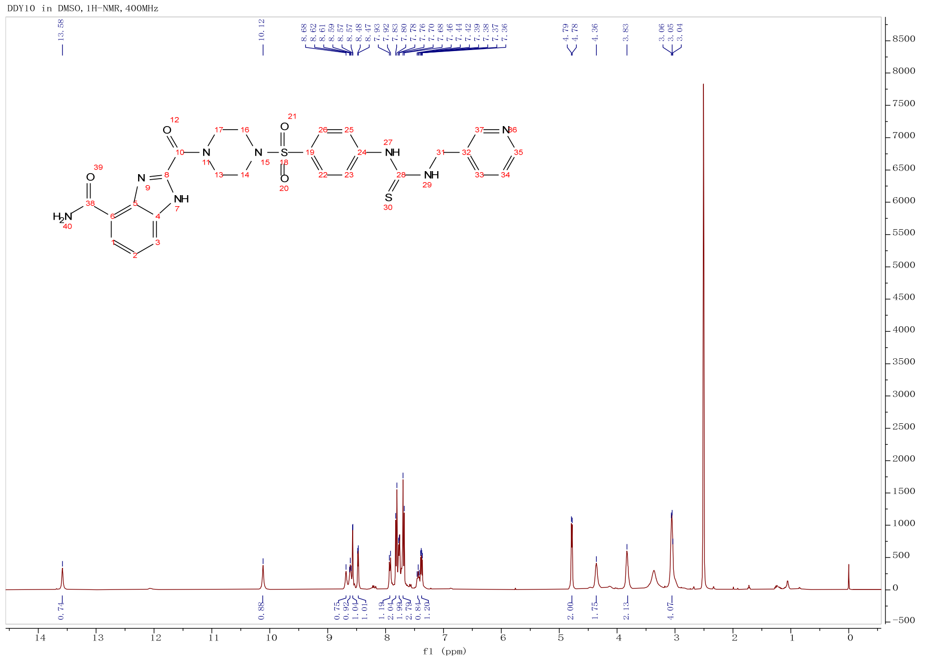

^{1}H-NMR spectrum of compound DDY10

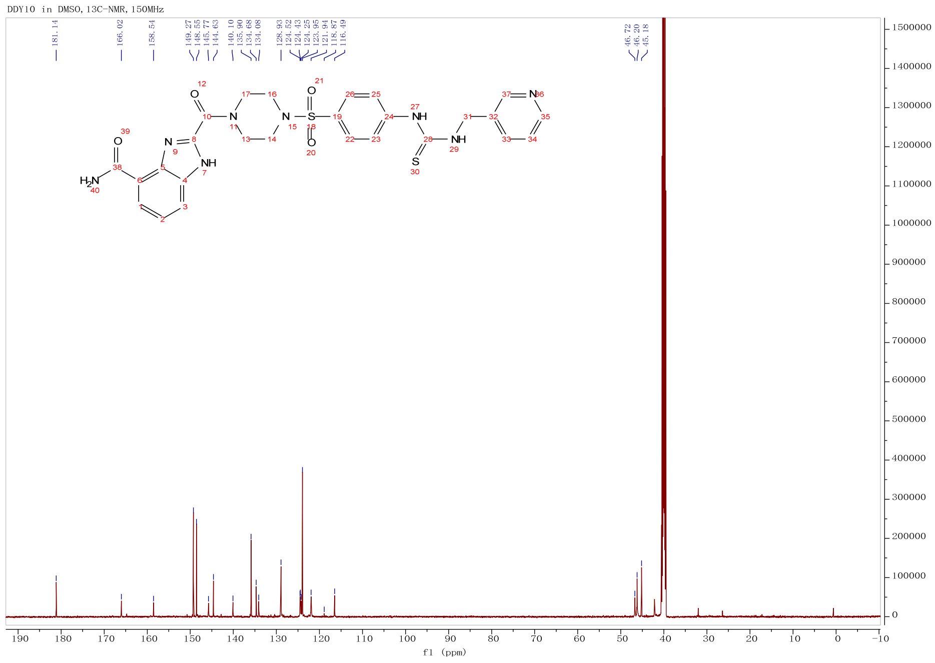

^{13}C-NMR spectrum of compound DDY10

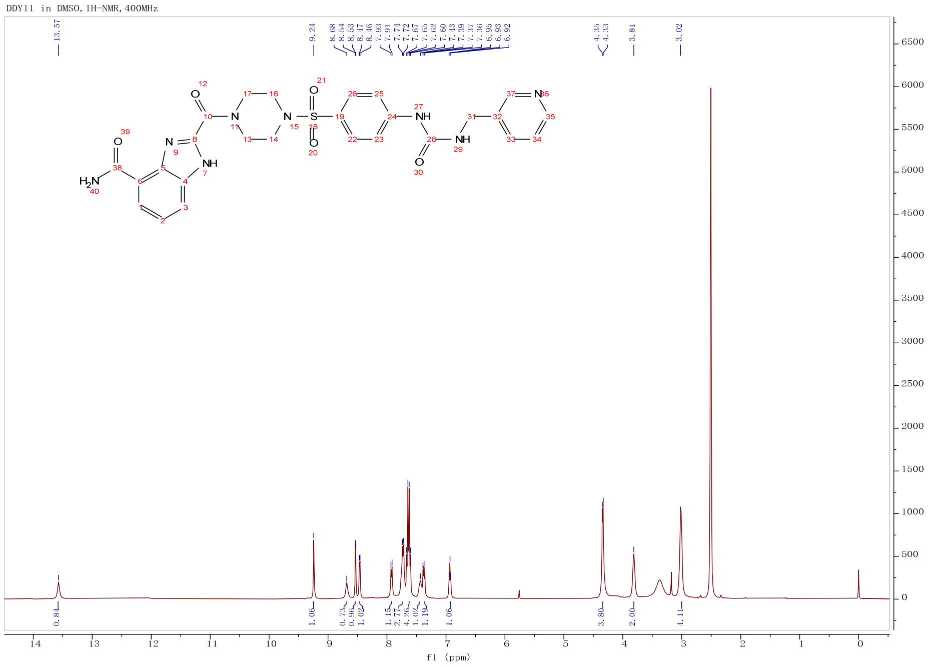

^{1}H-NMR spectrum of compound DDY11

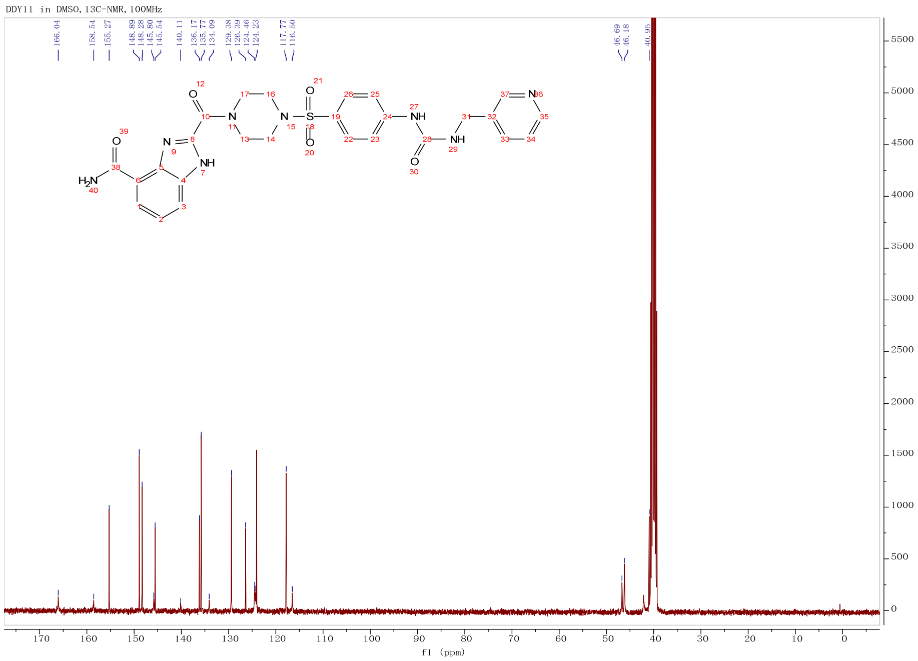

^{13}C-NMR spectrum of compound DDY11

4 IR spectrum data

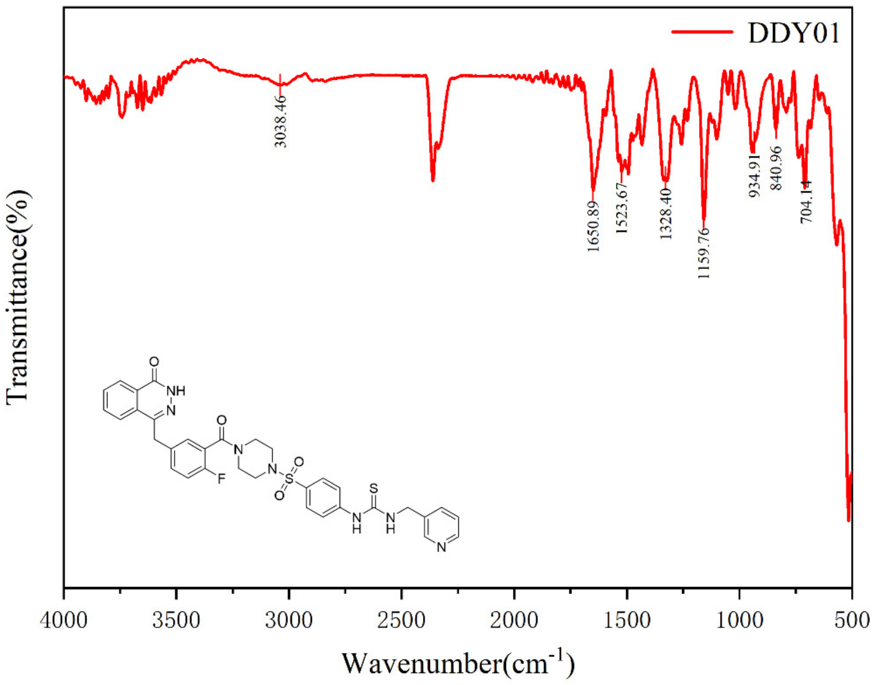

IR spectrum of compound DDY01

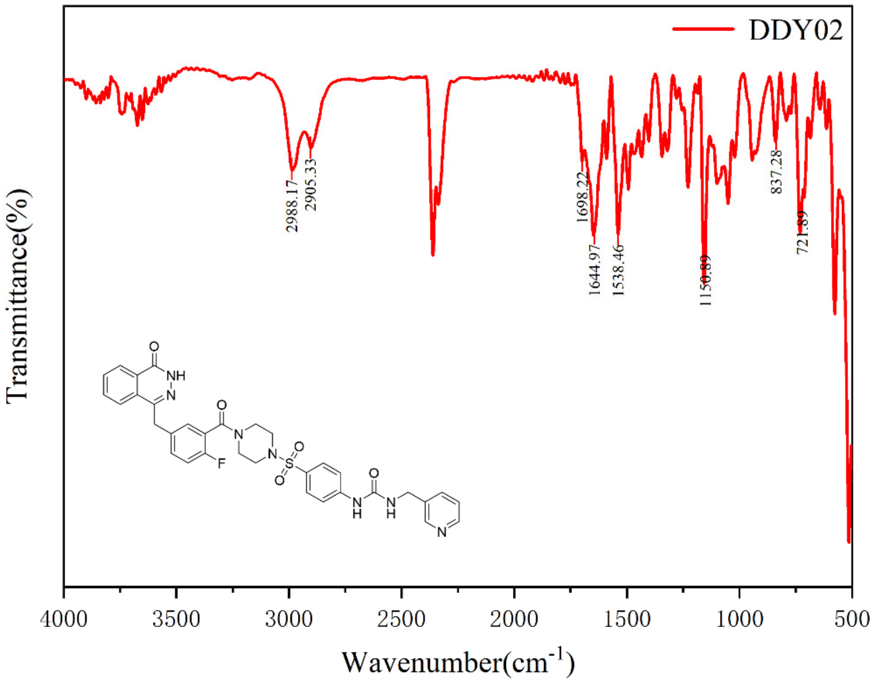

IR spectrum of compound DDY02

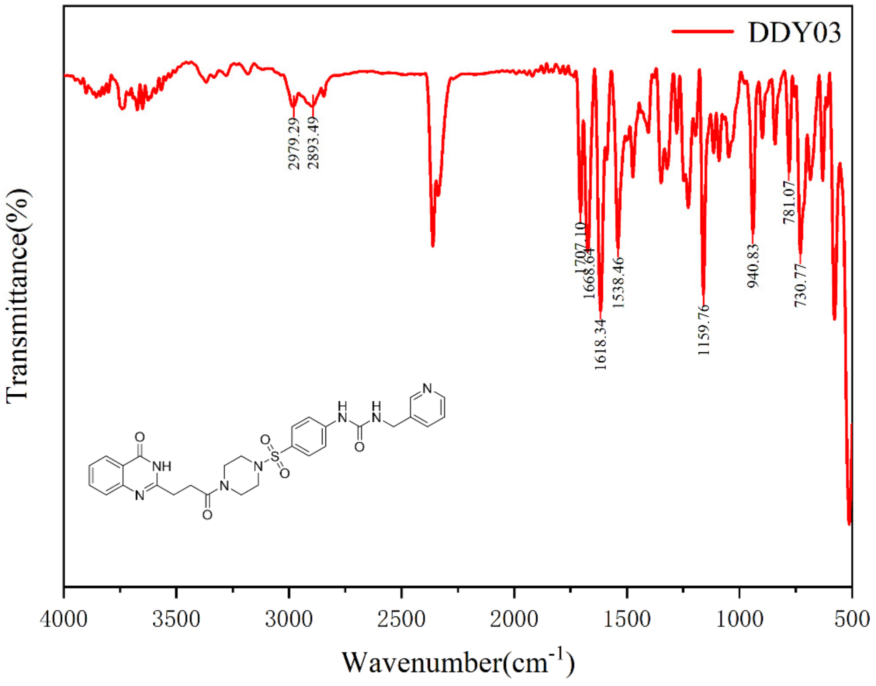

IR spectrum of compound DDY03

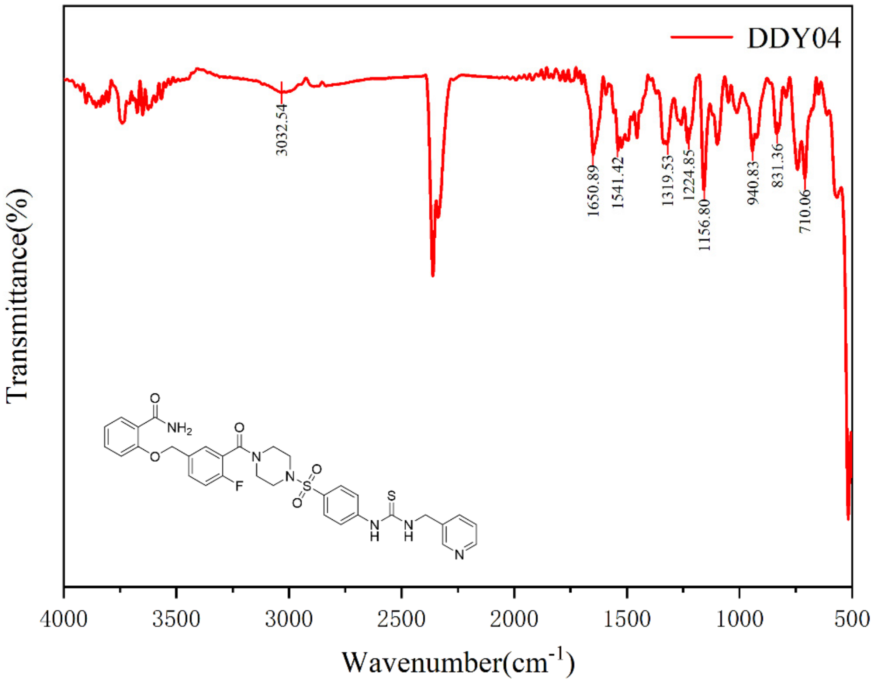

IR spectrum of compound DDY04

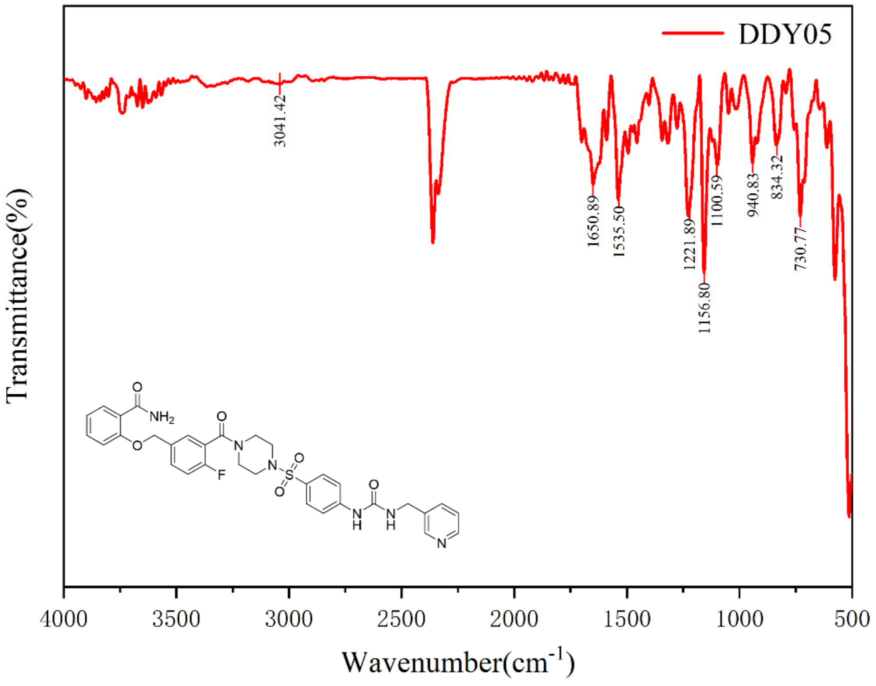

IR spectrum of compound DDY05

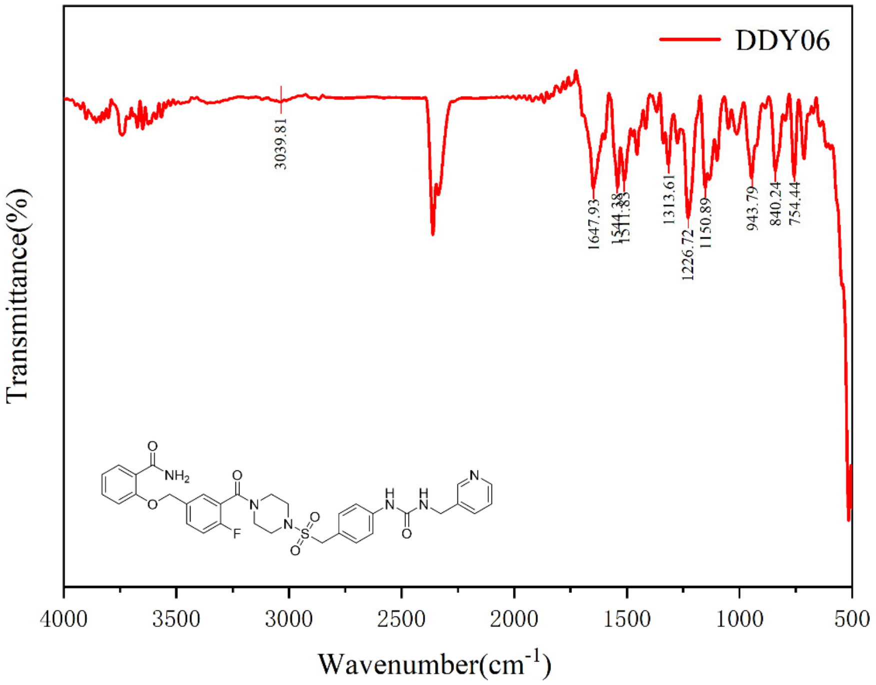

IR spectrum of compound DDY06

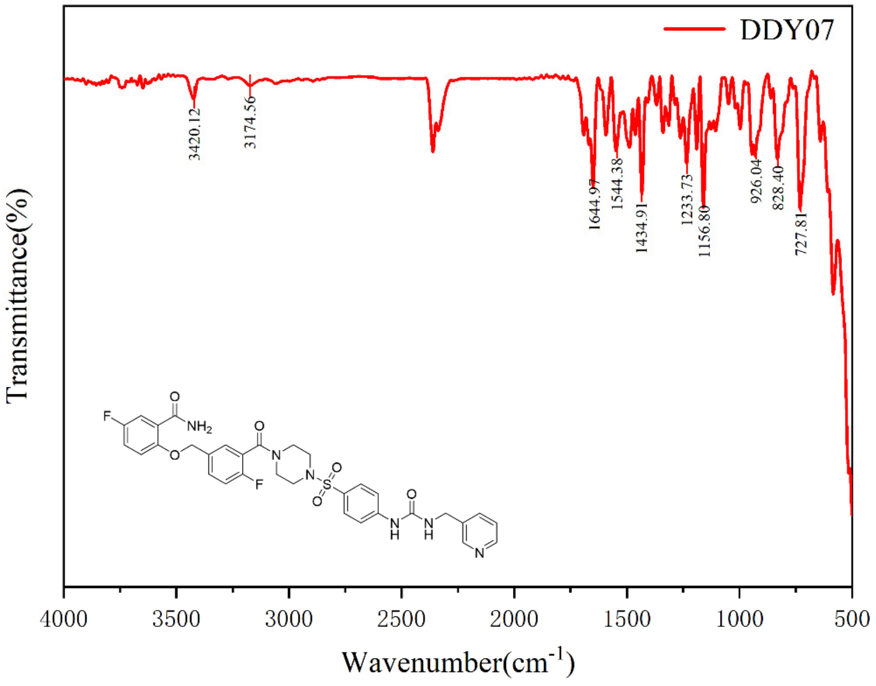

IR spectrum of compound DDY07

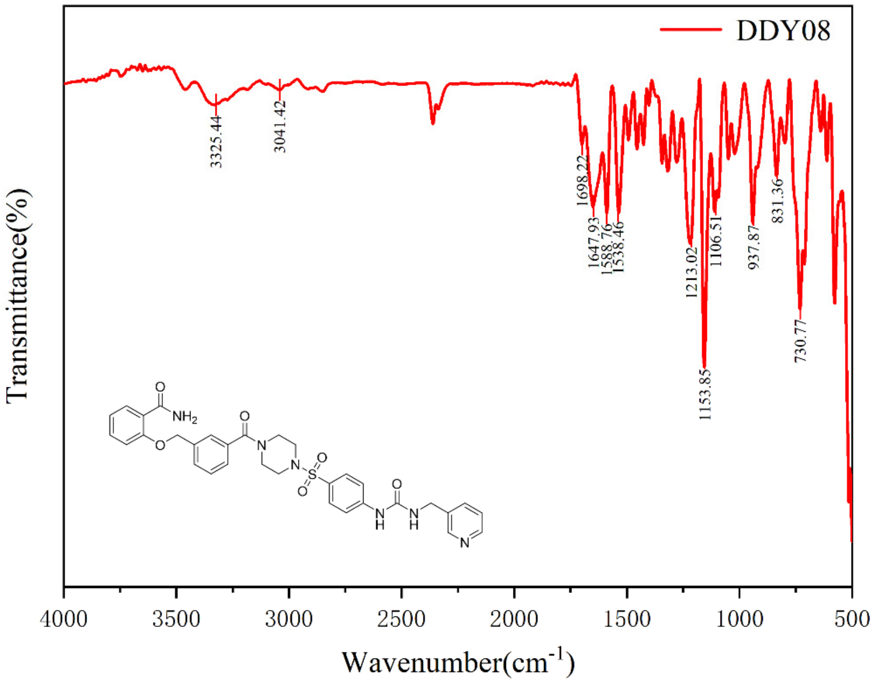

IR spectrum of compound DDY08

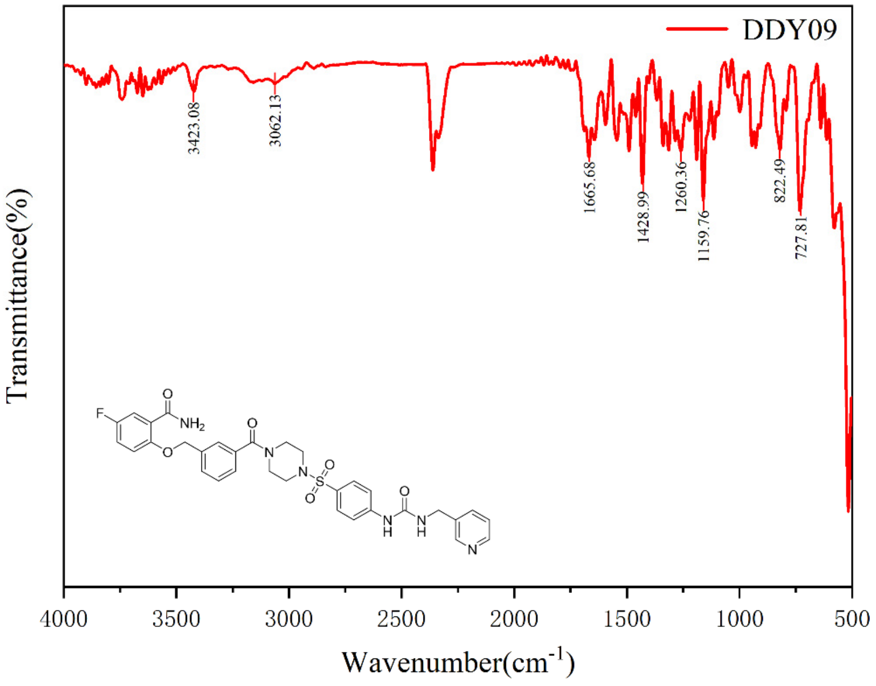

IR spectrum of compound DDY09

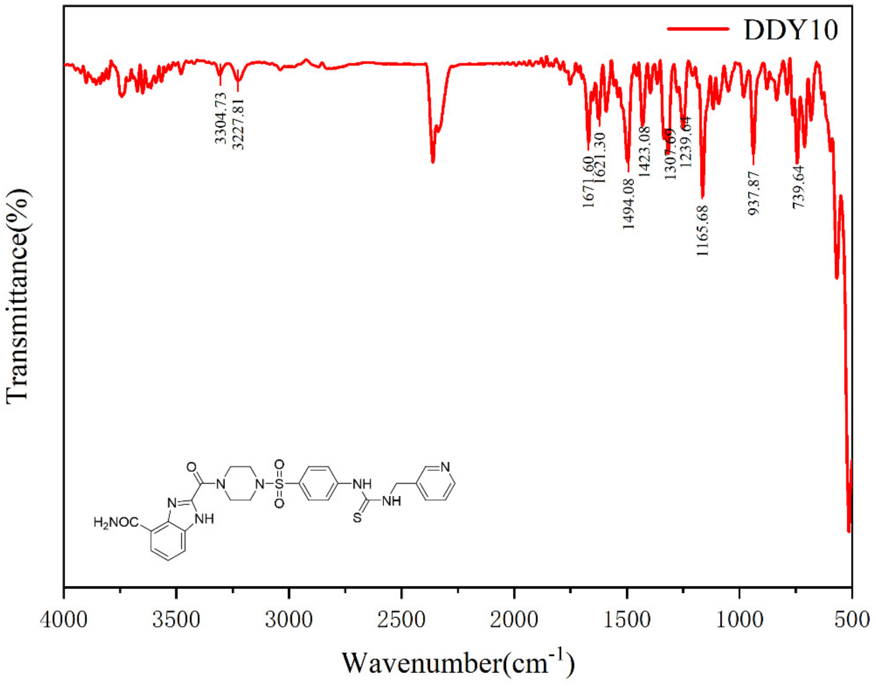

IR spectrum of compound DDY10

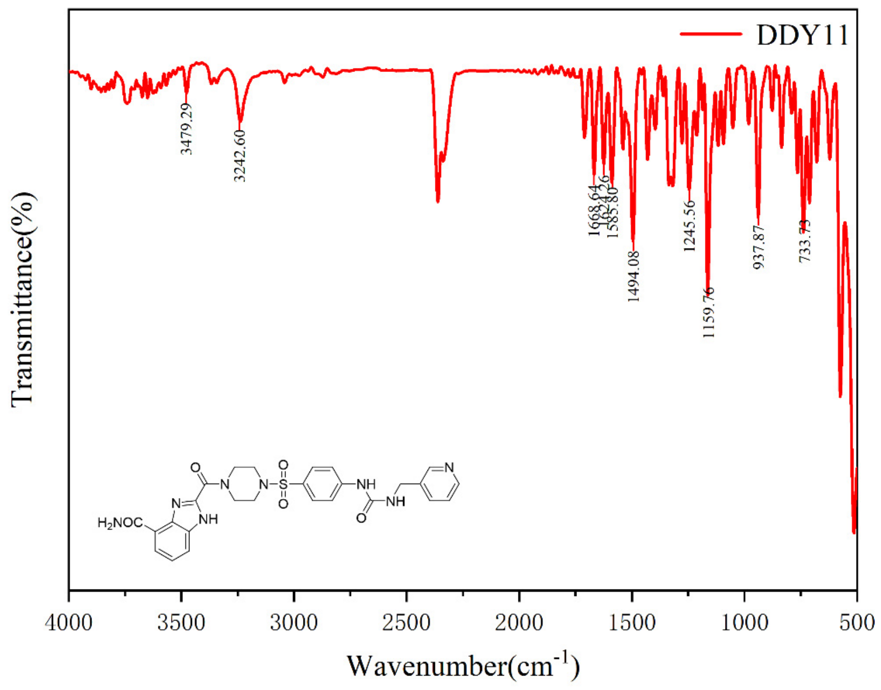

IR spectrum of compound DDY11

5 HPLC data

The purity of DDY01-DDY11 was determined by HPLC, meeting the experimental requirements. Here's a breakdown of the HPLC conditions:

Column: Diamonsil ®C18 (4.6×250 mm, 5μm).

Mobile Phase: Acetonitrile (solvent A) and 0.1% formic acid/water (solvent B)

Gradient: 0 min: 20% solvent A; 0-30 min: linear gradient from 20% to 60% solvent A; 30-35 min: linear gradient from 60% to 20% solvent A.

Detection: 254 nm.

Flow Rate: 1 mL/min.

Temperature: 30 ℃.

Injection Volume: 20 μL.

Concentration: 1 mg/mL (CH_3OH).

DDY01

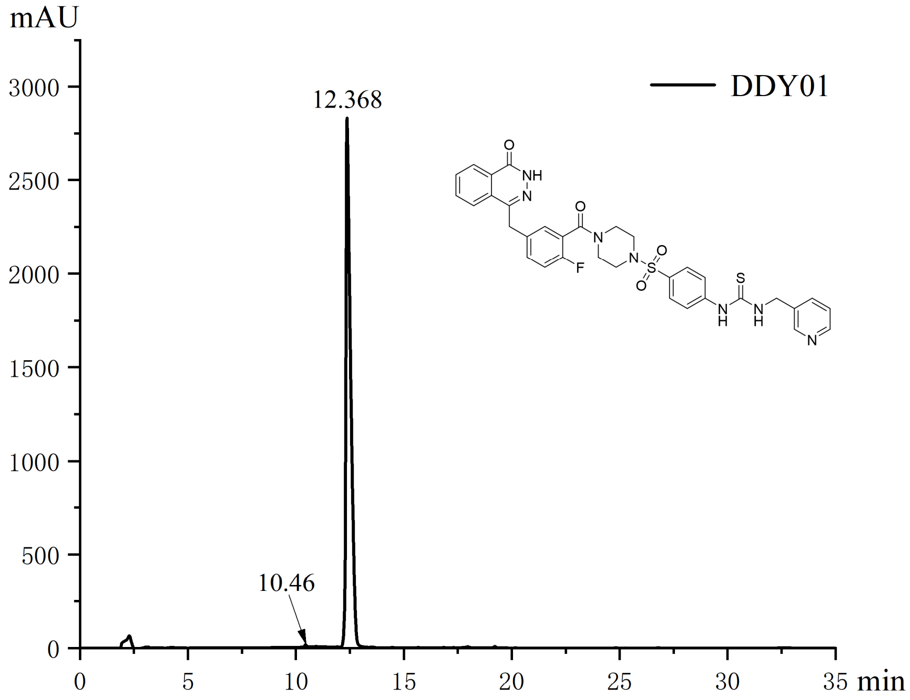

Peak	RT (min)	Peak Width (min)	Peak Height (mAU)	Peak Area (MAU*s)	Peak Area (%)
1	10.46	0.1136	15.5	63.8	0.148
2	12.368	0.2175	2828.6	43039.8	99.8520

DDY02

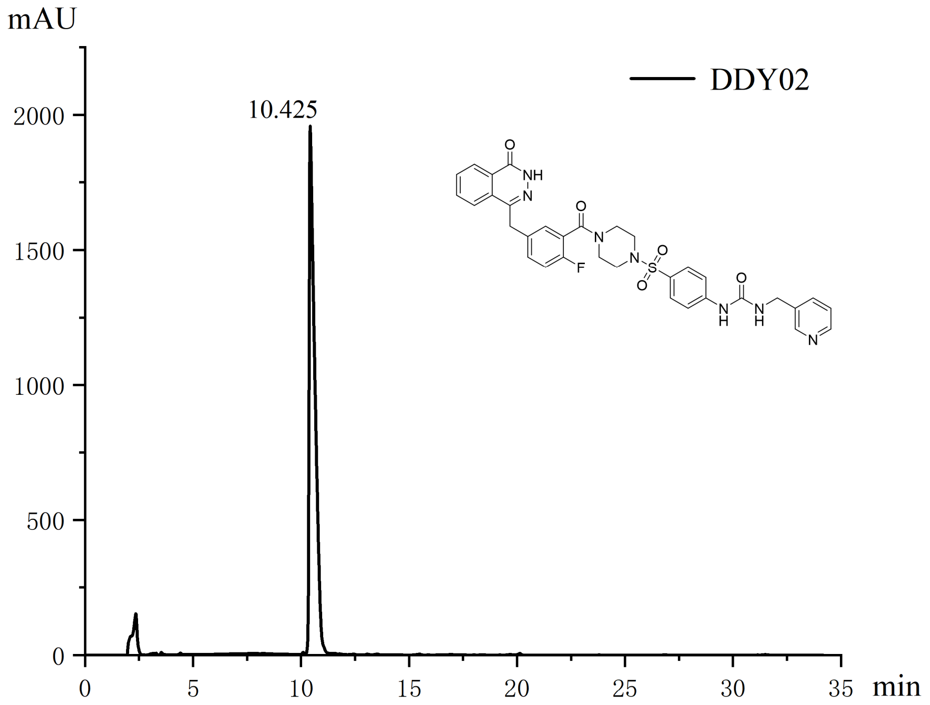

Peak	RT (min)	Peak Width (min)	Peak Height (mAU)	Peak Area (MAU*s)	Peak Area (%)
1	10.425	0.2512	1954.8	35382.7	100

DDY03

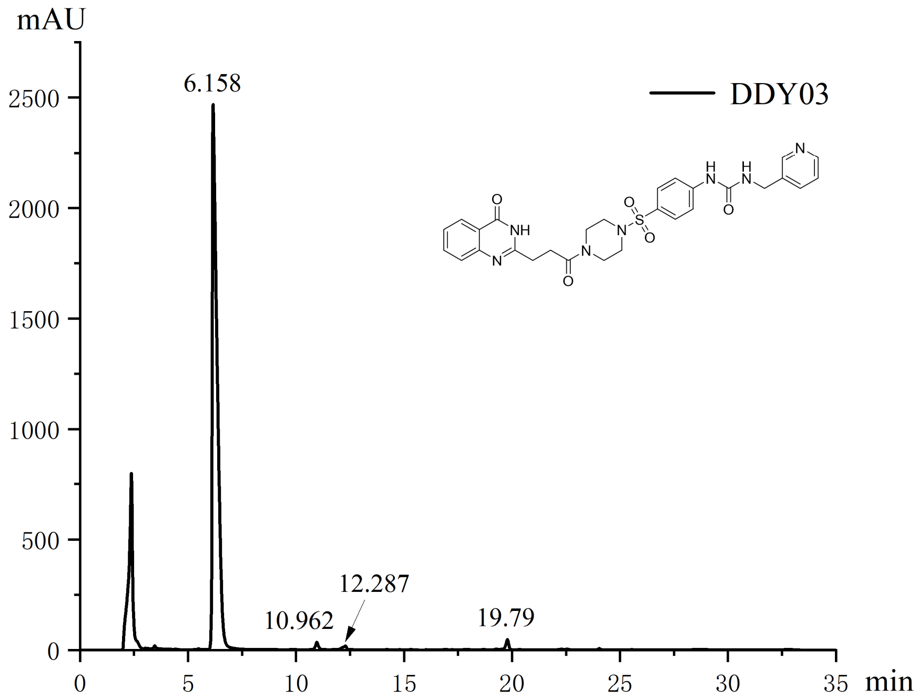

Peak	RT (min)	Peak Width (min)	Peak Height (mAU)	Peak Area (MAU*s)	Peak Area (%)
1	6.158	0.2262	2466.5	38935.4	97.5575
2	10.962	0.1494	33.2	345.6	0.8659
3	12.287	0.192	16.7	235.2	0.5901
4	19.79	0.1355	44.4	393.7	0.9865

DDY04

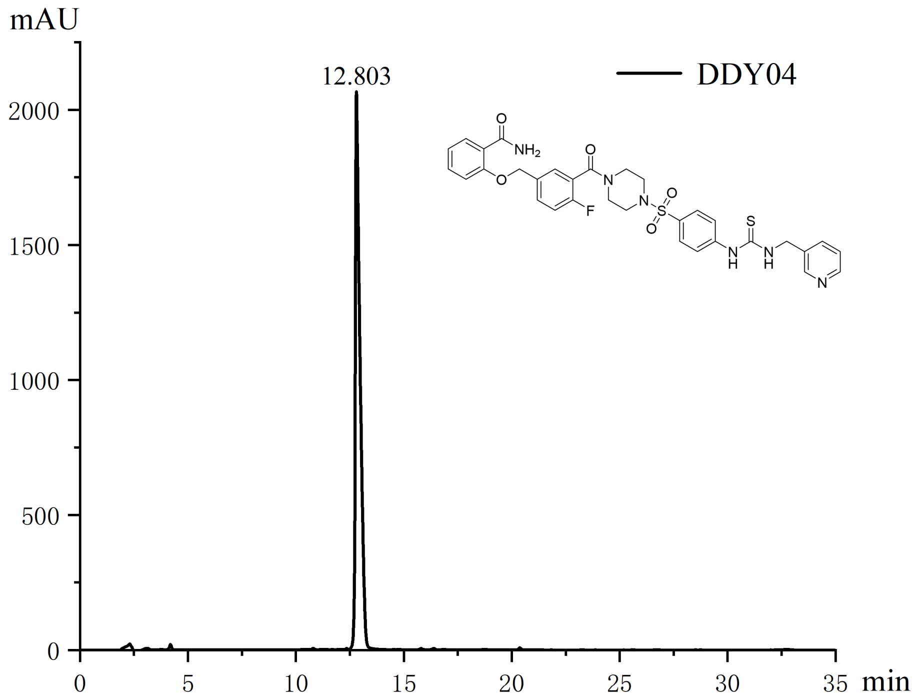

Peak	RT（min）	Peak Width（min）	Peak Height（mAU）	Peak Area（MAU*s）	Peak Area（%）
1	12.803	0.2066	2067.7	31303.1	100

DDY05

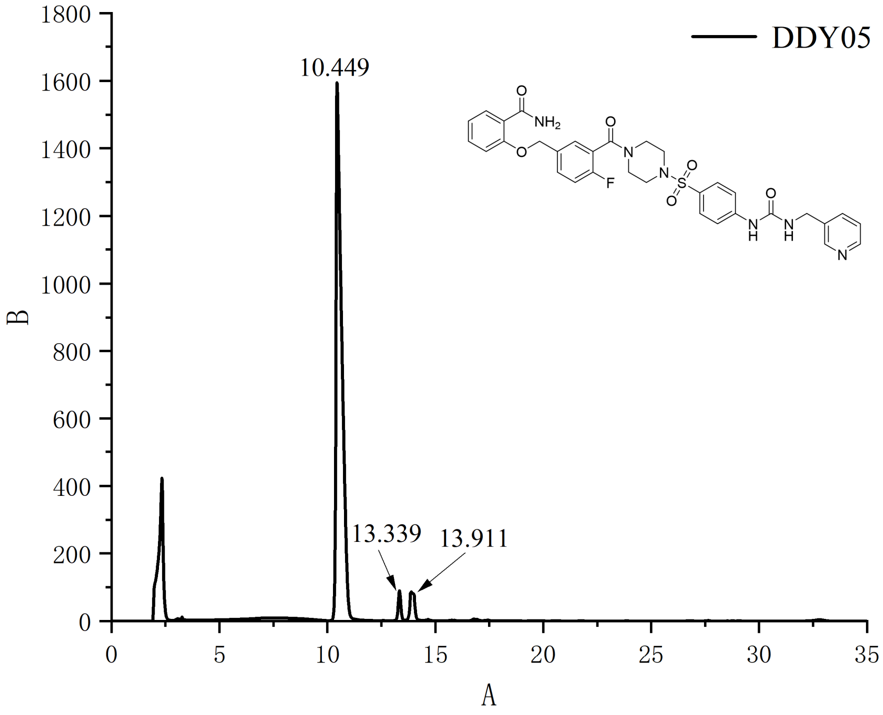

Peak	RT (min)	Peak Width (min)	Peak Height (mAU)	Peak Area (MAU*s)	Peak Area (%)
1	10.449	0.2548	1594.2	29071.1	95.4641
2	13.339	0.1278	87.3	632.1	2.0757
3	13.911	0.1477	86.9	749.2	2.4602

DDY06

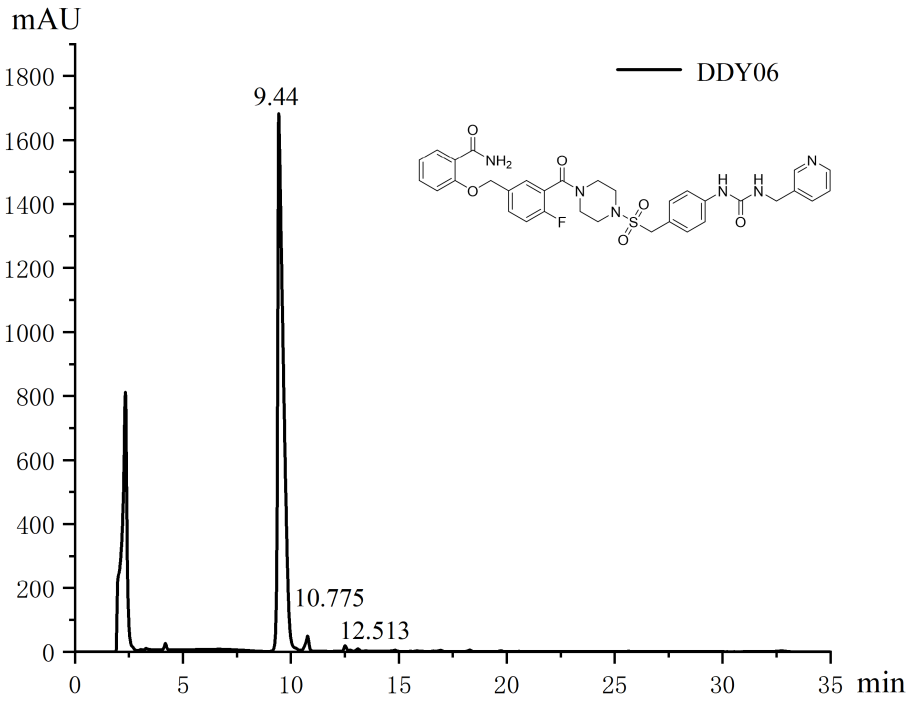

Peak	RT (min)	Peak Width (min)	Peak Height (mAU)	Peak Area (MAU*s)	Peak Area (%)
1	9.44	0.2624	1680	30588.8	98.0071
2	10.775	0.1515	45.9	493.8	1.5821
3	12.513	0.1271	16.1	128.2	0.4092

DDY07

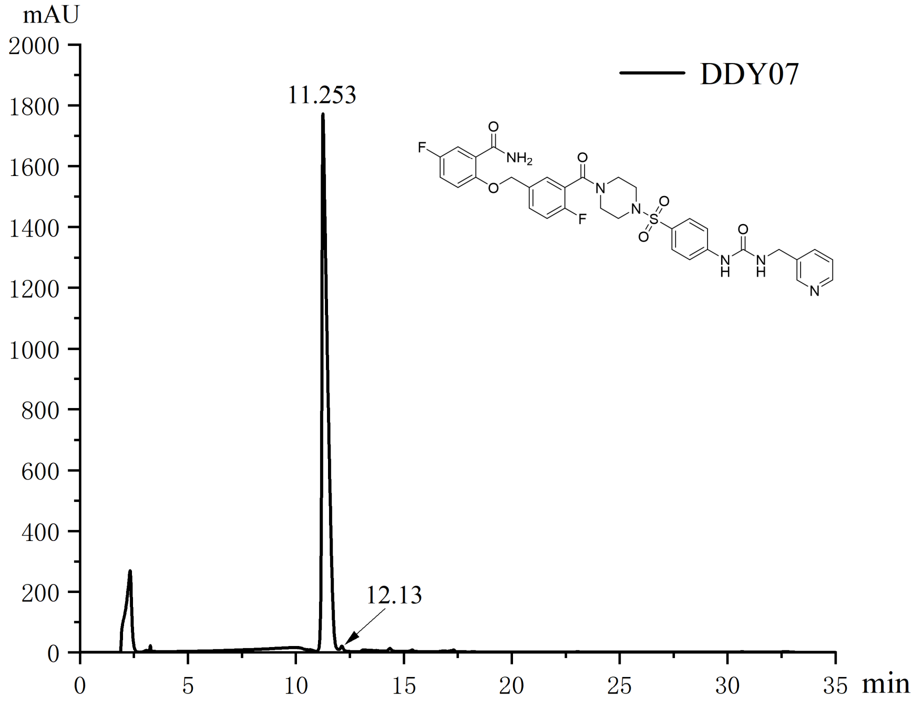

Peak	RT<br>（min）	Peak<br>Width<br>（min）	Peak<br>Height<br>（mAU）	Peak Area<br>（MAU*s）	Peak Area<br>（%）
1	11.253	0.256	1767.4	31757.9	99.9575
2	12.13	0.1177	13.5	13.5	0.0425

DDY08

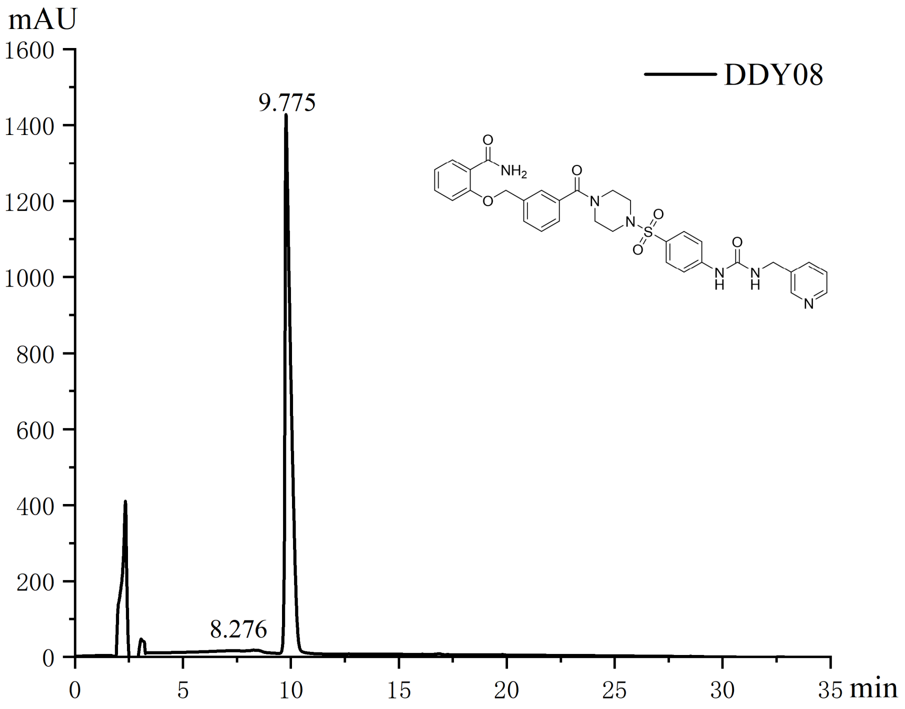

Peak	RT (min)	Peak Width (min)	Peak Height (mAU)	Peak Area (MAU*s)	Peak Area (%)
1	8.276	0.4894	4.1	155.8	0.5878
2	9.775	0.2504	1420.5	26351.6	99.4122

DDY09

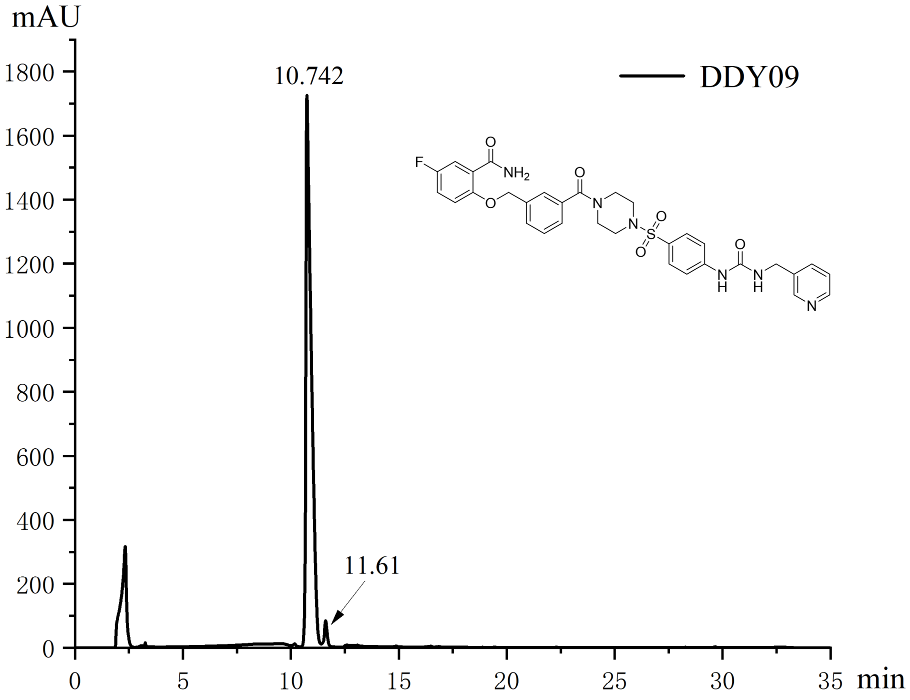

Peak	RT (min)	Peak Width (min)	Peak Height (mAU)	Peak Area (MAU*s)	Peak Area (%)
1	10.742	0.2361	1723.3	29848.6	97.3977
2	11.61	0.1427	82.5	797.5	2.6023

DDY10

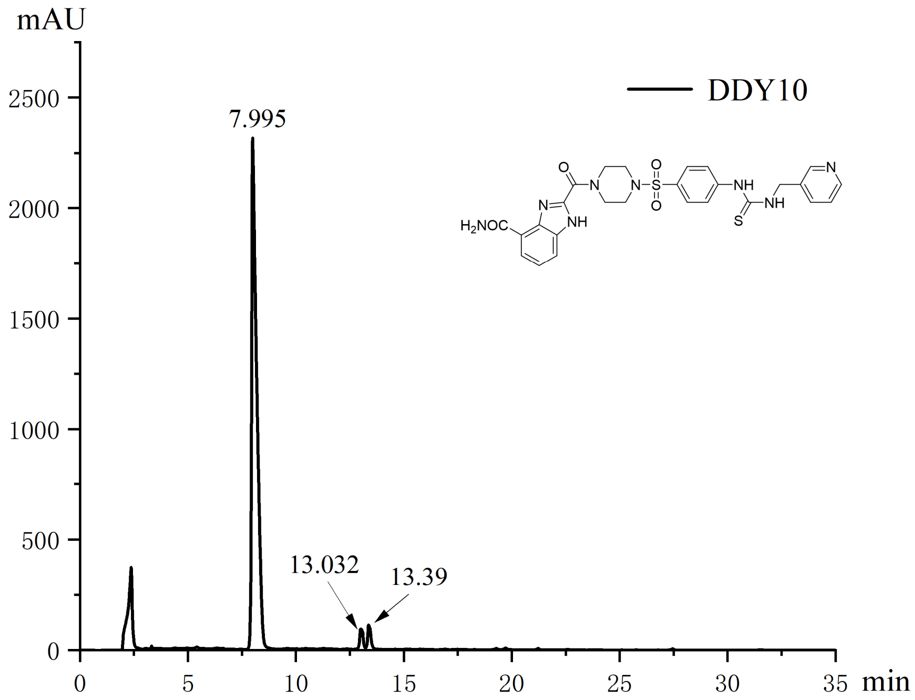

Peak	RT (min)	Peak Width (min)	Peak Height (mAU)	Peak Area (MAU*s)	Peak Area (%)
1	7.995	0.2209	2314.8	37869.1	95.9268
2	13.032	0.1354	108.6	818.2	2.0720
3	13.39	0.1446	114.5	789.8	2.0012

DDY11

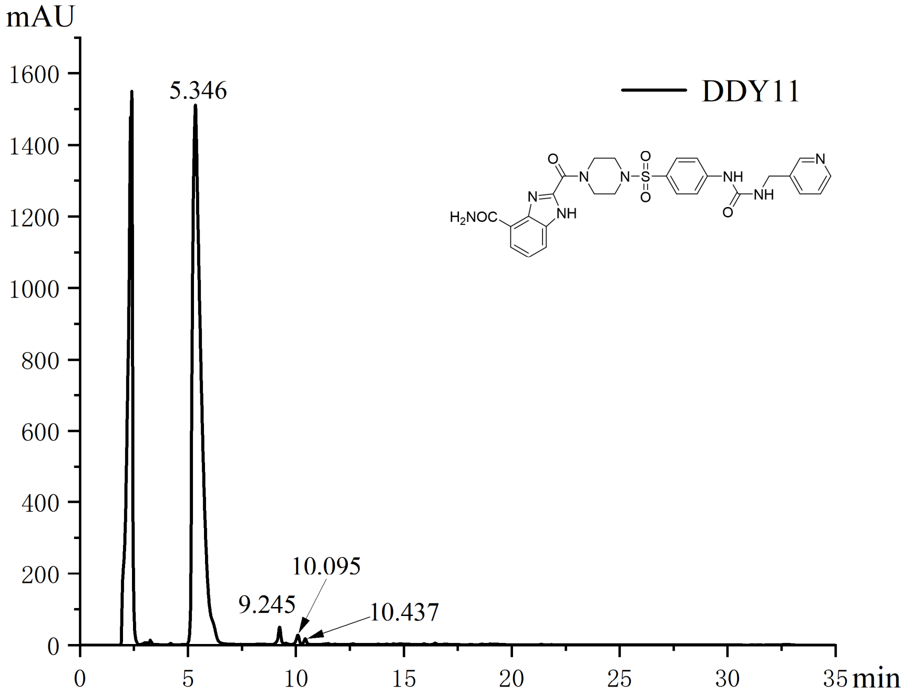

Peak	RT (min)	Peak Width (min)	Peak Height (mAU)	Peak Area (MAU*s)	Peak Area (%)
1	5.346	0.4017	1808.7	50185.7	98.7659
2	9.245	0.1229	21.8	181	0.3562
3	10.095	0.1432	31.2	292.2	0.5751
4	10.437	0.1208	18.9	153.9	0.3028
